# Fisher-Information-Based Cooperative Sensor Node Pre-Selection for UWB-Aided GNSS-Denied UAV Swarm Localization Under Heterogeneous Ranging Noise

**DOI:** 10.3390/s26165164

**Published:** 2026-08-14

**Authors:** Yanming Sun, Xiaoyan Du, Pihong Gong

**Affiliations:** 1 School of Transportation, Shandong University of Science and Technology, Qingdao 266590, China; 202482160015@sdust.edu.cn; 2 International Cooperation Center, National Development and Reform Commission, Beijing 100038, China; gphongwork@163.com

**Keywords:** UWB cooperative localization, cooperative sensor node pre-selection, Fisher information matrix, generalized GDOP, heterogeneous ranging noise, resource-constrained sensing, GNSS-denied navigation

## Abstract

**Highlights:**

**What are the main findings?**

- A Fisher-information-based generalized GDOP metric is developed to evaluate the local observability of UWB-aided cooperative ranging sensor networks under topology-coupled and heteroscedastic link-noise conditions.
- A resource-constrained greedy cooperative sensor node pre-selection heuristic is developed to jointly control the number of selected nodes, induced active ranging links, ranging-slot occupation, and general sensing-resource cost.

**What are the implications of the main findings?**

- The proposed method provides an interpretable information-theoretic criterion for cooperative sensor node pre-selection before cooperative localization or factor-graph optimization in GNSS-denied environments.
- The framework explicitly distinguishes the selected-node budget from the induced-link and ranging-slot budgets, while all mobile nodes remain in the cooperative localization state.

**Abstract:**

Ultra-wideband (UWB) inter-node ranging provides relative-distance constraints for cooperative localization in GNSS-denied UAV swarms, but dense candidate networks can exceed the available ranging slots, communication bandwidth, computation, and energy. This paper proposes a Fisher-information-based cooperative sensor node pre-selection method under heterogeneous ranging noise. All mobile nodes remain in the localization state, while the selected nodes induce the active ranging-link set. Selected-node, induced-link, ranging-slot, and normalized general-resource budgets are represented separately. Using predicted geometry and estimated link-quality weights, a gauge-free normalized Fisher information matrix combines link geometry, link-quality-dependent weights, and topology-induced coupling. A trace-based generalized GDOP (G-GDOP) criterion is optimized by a two-stage greedy heuristic with recursive matrix updates. The experiments show that G-GDOP is a local observability and information-quality metric rather than a direct predictor of topology-level nonlinear recovery error. Within the same topology, normalized local RMSE increased from 0.698 [0.673, 0.752] in the Low G-GDOP group to 1.014 [0.999, 1.068] and 1.980 [1.806, 2.180] in the Medium and High groups. Increasing the selected-node budget from *K* = 6 to *K* = 20 reduced median RMSE from 0.550 to 0.148 m while increasing the median induced-link number from 109 to 214. Additional tests covered Gaussian and heterogeneous ranging noise, deterministic NLOS bias, online link-weight errors, and predicted-position uncertainty. Direct Inversion and Woodbury Updating were numerically equivalent within a predefined tolerance in all 24 size–regime combinations, and a Woodbury runtime advantage was supported in 20 conditions. The proposed framework therefore provides an interpretable resource-aware pre-selection module without implying an unconditional real-time guarantee.

## 1. Introduction

Reliable positioning and navigation are fundamental requirements for unmanned aerial vehicles (UAVs), mobile robots, and cooperative sensing systems operating in GNSS-denied or GNSS-degraded environments. In urban canyons, indoor–outdoor transition areas, cluttered low-altitude spaces, and post-disaster scenarios, GNSS signals are often affected by blockage, attenuation, multipath propagation, and non-line-of-sight (NLOS) effects, which may reduce positioning availability and degrade localization accuracy. To overcome the limitations of standalone GNSS positioning, various onboard and inter-node sensing sources, such as ultra-wideband (UWB) ranging, inertial measurement units (IMUs), visual-inertial odometry (VIO), simultaneous localization and mapping (SLAM), and range-based cooperative measurements, have been introduced into UAV and robotic localization systems [1,2,3,4,5,6,7]. Among these sensing technologies, UWB-based inter-node ranging is particularly attractive for cooperative localization because it can provide direct relative distance constraints with relatively low cost, low power consumption, and high temporal resolution. UWB waveform design and admissible emission spectra are constrained by regional regulations and pulse-shaping requirements, while the ranging error observed by a practical receiver depends strongly on propagation conditions, reflections, interference, and fading [8,9]. These physical effects motivate the heterogeneous random-error and residual bias stress tests used later in this paper, but the present work does not claim to reproduce a complete radio-frequency channel or transceiver model.

In a UAV swarm or multi-robot system equipped with UWB ranging sensors, the inter-node ranging links naturally form a cooperative ranging sensor network. Each UAV-borne sensor node can provide not only its own motion information, but also relative measurements to neighboring nodes. These relative measurements can improve the local observability of the network and reduce the accumulated positioning drift caused by GNSS outages or onboard sensor errors. Recent studies on UAV swarms and multi-robot systems have demonstrated the potential of formation-constrained cooperative localization, cluster-based localization, and multi-condition cooperative positioning in GNSS-denied environments [10,11,12]. However, in dense cooperative sensing scenarios, the number of potential ranging links is usually much larger than the number that can be activated simultaneously. Communication bandwidth, ranging time slots, onboard computation, and residual energy all restrict the scale of cooperative participation.

Therefore, a key issue in resource-constrained cooperative localization is not only how to estimate the positions of all mobile nodes from available measurements, but also which mobile nodes should be included in the selected node subset under limited resources. In the framework considered in this paper, individual ranging links are not optimized as independent decision variables. Instead, the active ranging-link set is induced by the selected node subset and the candidate ranging graph.

Geometric dilution of precision (GDOP) is a classical and interpretable metric for evaluating the influence of observation geometry on localization accuracy. It has been widely used in satellite navigation, wireless positioning, and geometric configuration analysis because of its clear physical meaning and low computational cost. Nevertheless, conventional GDOP is mainly developed for single-target localization under homoscedastic observation assumptions. This setting is different from cooperative ranging sensor networks, where multiple unknown nodes are estimated jointly and inter-node measurements introduce block-coupled information structures. Moreover, the quality of different UWB ranging links may vary significantly due to inter-node distance, antenna orientation, occlusion, multipath propagation, NLOS conditions, and environmental interference. As a result, the localization contribution of each link is jointly affected by link geometry, heterogeneous measurement noise, and cooperative network topology. A direct application of conventional GDOP is therefore insufficient to characterize the system-level information quality of topology-coupled cooperative localization networks [13,14,15,16,17].

The Fisher information matrix (FIM) and the Cramér–Rao lower bound (CRLB) provide a rigorous statistical framework for analyzing localization accuracy and theoretical error lower bounds. FIM- and equivalent-FIM-based methods can incorporate measurement geometry, noise statistics, and network topology into a unified information representation, and have been widely used in wireless sensor network localization, cooperative navigation, factor-graph-based localization, sensor selection, and link scheduling [14,15,18,19,20,21,22,23]. However, directly applying information-theoretic criteria to sensor node or link selection may introduce considerable computational burden, especially when the number of candidate nodes or links increases. Existing studies have investigated position-error-bound-based selection, game-theoretic resource allocation, topology optimization, and cooperative link selection strategies [19,20,21,22,23]. Although these methods have improved cooperative localization performance from different perspectives, an efficient and interpretable node selection criterion is still needed for UWB-aided cooperative localization, where link geometry, heterogeneous ranging noise, and multi-node topological coupling should be evaluated jointly while maintaining acceptable computational complexity.

The trace-inverse A-optimal objective, marginal-gain greedy selection, and Woodbury identity are established mathematical tools and are not claimed here as new primitives. The contribution of this study is their problem-specific integration into an anchor-free UWB cooperative sensor node pre-selection framework. The decision variable is the selected node subset; usable links are induced by that subset, heterogeneous online link-quality estimates enter the projected information matrix, and node, link, slot, and general resource limits are treated explicitly. Multiplication of the physical FIM by the positive reference factor $\sigma_{0}^{2}$ only changes the numerical scale. Likewise, division by the common observable dimension $d_{o}$ does not change the minimizing subset when the total state and predicted geometry are fixed; it is introduced only to make reported objective values interpretable across network sizes. To provide a clearer comparison of existing studies on cooperative localization and node selection, representative works are summarized in Table 1.

As summarized in Table 1, existing studies have made important progress in GNSS-denied positioning, multi-sensor fusion, cooperative localization, topology-aware estimation, and sensor node or measurement-selection techniques. However, dense UWB cooperative ranging networks still lack an interpretable and computationally efficient pre-selection framework that can jointly evaluate link geometry, heterogeneous ranging reliability, and topology-induced multi-node coupling under limited ranging and communication resources. This motivates the development of a Fisher-information-based cooperative sensor node pre-selection framework for resource-constrained UWB cooperative localization.

The main contributions of this paper are summarized as follows:

1. A UWB cooperative ranging network model is established for resource-constrained cooperative sensor node pre-selection under heterogeneous ranging noise conditions. The proposed formulation distinguishes between the complete localization state and the selected node subset. All *N* mobile sensor nodes remain in the localization state, whereas only the selected nodes are scheduled as active UWB ranging initiators. The corresponding active ranging-link set is induced by the selected node subset and is not optimized independently.
2. A trace-based A-optimal criterion is specialized to the topology-coupled UWB pre-selection problem and reported through the G-GDOP notation. It combines UWB link-direction geometry, estimated heterogeneous ranging noise weights, and multi-node coupling in a gauge-free local information-quality measure; its normalization does not change the selected node subset for a fixed predicted network state.
3. A resource-constrained cooperative sensor node pre-selection model is formulated by separately representing the selected-node budget, induced active-link number, ranging-slot occupation, and general sensing-resource cost. A marginal-gain-based greedy heuristic with recursive information matrix updates is then developed to construct a feasible selected node subset without repeatedly inverting the complete candidate information matrix.
4. A comprehensive nine-experiment validation framework is established to clarify the applicability boundary and same-topology ranking behavior of G-GDOP, quantify node-, induced-link-, ranging-slot-, and general-resource trade-offs, and compare the proposed heuristic with the small-scale exhaustive optimum. The framework also evaluates Gaussian noise, heterogeneous link quality, deterministic NLOS bias, online weight-estimation errors, predicted-position uncertainty, and the tolerance-qualified numerical equivalence and runtime scaling of Direct Inversion and Woodbury Updating. Shared non-success and common-unobservable cases are retained in the formal status accounting rather than being silently discarded.

The remainder of this paper is organized as follows. Section 2 presents the UWB-aided cooperative sensor network model and constructs the generalized GDOP metric. Section 3 formulates the resource-constrained cooperative sensor node pre-selection problem and introduces the proposed greedy algorithm with recursive matrix updates. Section 4 reports the simulation settings and experimental results. Section 5 concludes the paper and outlines future research directions.

## 2. UWB-Aided Cooperative Sensor Network Modeling and Generalized GDOP Metric

### 2.1. Problem Definition of Cooperative Sensor Node Pre-Selection

Consider a GNSS-denied UAV swarm consisting of *N* mobile sensor nodes equipped with onboard motion sensors and UWB ranging modules. The candidate mobile sensor node set is defined as

C={1,2,…,N}.

An optional set of pre-surveyed reference nodes is denoted by A. The candidate UWB ranging network is represented by the undirected graph

$$G_{c}=\left(C\cup A,E_{c}\right),$$

where $E_{c}$ contains all candidate UWB ranging links that satisfy the communication-range and ranging-availability conditions. In dense cooperative ranging networks, activating all candidate sensor nodes and all available UWB ranging links may exceed the available ranging time slots, communication bandwidth, onboard computational capacity, or energy budget. Therefore, at each ranging-scheduling epoch, only a subset of cooperative sensor nodes is selected for active ranging. The selected node subset is denoted by

S⊆C,|S|=K,

where *K* is the prescribed selected-node budget. This study optimizes the selected node subset S rather than independently selecting individual UWB ranging links. The selected nodes are scheduled as active UWB ranging initiators, whereas the remaining mobile nodes can still serve as ranging responders and remain part of the localization state. Once S is determined, the corresponding active ranging-link set is induced as

(1) $$\begin{array}{c} E(S)=\left\{\left(i,j\right)\in E_{c}|i,j\in C,\;i\in S\;\mathrm{or}\;j\in S\right\}\\ \;\cup \left\{\left(i,a\right)\in E_{c}|i\in S,\;a\in A\right\}. \end{array}$$

Therefore, cooperative sensor node pre-selection controls the selected node subset and its induced ranging operations, rather than independently optimizing each individual UWB link. The main scenario considered in this study is anchor-free cooperative ranging, for which

A=⌀.

In this case, the active ranging-link set reduces to

$$E\left(S\right)=\left\{\left(i,j\right)\in E_{c}|i,j\in C,\;i\in S\;\mathrm{or}\;j\in S\right\}.$$

The formal problem and all reported experiments in this paper use the anchor-free case. The anchor-assisted expression is retained only to show how the same induced-link notation could be extended when independently surveyed anchors are available; no known-position anchor is introduced into the reported GNSS-denied scenario.

For each candidate mobile-node ranging link $e=\left(u,v\right)\in E_{c}$, define the selection-dependent link activation indicator as

(2) $$\chi_{e}\left(S\right)=\left\{\begin{array}{cc} 1,\; & u\in S\;\mathrm{or}\;v\in S,\\ 0,\; & u\notin S\;\mathrm{and}\;v\notin S. \end{array}\right.$$

The induced active ranging-link set can equivalently be written as

$$E\left(S\right)=\left\{e\in E_{c}\;|\;\chi_{e}\left(S\right)=1\right\}.$$

Accordingly, an inter-node ranging link becomes active when at least one of its two endpoints is selected as an active UWB ranging initiator. If both endpoints are unselected, the corresponding link remains inactive. Each candidate ranging link is included at most once in E(S), even when both of its endpoints belong to S.

For any mobile sensor node i∈C, its three-dimensional position is expressed as

$$p_{i}={\left[x_{i},y_{i},z_{i}\right]}^{T}\in R^{3}.$$

The localization state contains the positions of all *N* mobile sensor nodes and is written as

$$x={\left[p_{1}^{T},p_{2}^{T},\ldots ,p_{N}^{T}\right]}^{T}\in R^{3N}.$$

The selected node subset S determines the active ranging measurements but does not change the dimension of the localization state. Consequently, the information matrices corresponding to different candidate subsets have the same dimension, which enables consistent comparison across node budgets and recursive low-rank matrix updates.

A mobile node belonging to the selected node subset S is termed a selected nodeand is scheduled as an active UWB ranging initiator. Here, active UWB ranging initiator describes the ranging role of a selected node rather than a separate decision variable. Unselected mobile nodes remain unknown state variables and may serve as passive ranging responders through candidate UWB links. Thus, the selection decision controls the active ranging schedule and the induced measurement topology without changing the complete mobile-node state. Unless otherwise stated, the proposed method performs cooperative sensor node pre-selection only; individual UWB ranging links are not treated as independent binary decision variables.

In practical GNSS-denied operation, the information matrix used for cooperative sensor node pre-selection is constructed from predicted or estimated node positions, such as those propagated from inertial sensing, visual odometry, or the localization result obtained at the previous epoch. For mobile node *i*, the predicted position is denoted by

$${\hat{p}}_{i}={\left[{\hat{x}}_{i},{\hat{y}}_{i},{\hat{z}}_{i}\right]}^{T}.$$

The predicted state of the complete mobile network is written as

$$\hat{x}={\left[{\hat{p}}_{1}^{T},{\hat{p}}_{2}^{T},\ldots ,{\hat{p}}_{N}^{T}\right]}^{T}.$$

The online cooperative sensor node pre-selection algorithm evaluates the ranging geometry at $\hat{x}$ rather than at the unknown true state x. The true node positions are used only as an oracle reference and for performance evaluation in the simulation experiments. This distinction removes the unrealistic assumption that the online selector has access to ground-truth geometry. Section 4.1.3 therefore evaluates sensitivity to snapshot-wise Gaussian position perturbations while holding the candidate graph, link-quality information, measurement realization, and resource constraints fixed. The broader limitations of this controlled uncertainty model are discussed in Section 4.3.

Since an anchor-free range-only network is invariant to global translation and rotation, its Fisher information matrix is singular in the full 3N-dimensional state space. The rigid-body gauge directions are therefore removed by projection onto their orthogonal complement. It should be emphasized that removing the global rigid-body gauge directions does not automatically guarantee local observability. Disconnected or non-rigid active-ranging topologies may introduce additional unobservable directions. Consequently, the projected information matrix must additionally satisfy a full-rank condition in the gauge-free subspace. The corresponding construction and numerical rank test are introduced in Section 2.3.

### 2.2. UWB Ranging Sensor Model and Link-Quality Weighting

Different UWB ranging links may exhibit heterogeneous random errors and systematic positive biases. For an active link e=(i,j), the bias-aware ranging model is

(3) $$z_{e}=h_{e}\left(x\right)+b_{e}+n_{e},$$

where $b_{e}$ is the systematic bias and $n_{e}∼N\left(0,\sigma_{e}^{2}\right)$ is the zero-mean random error. For a mobile-node–mobile-node link, $h_{e}\left(x\right)={∥p_{i}-p_{j}∥}_{2}$, whereas for a mobile-node–anchor-node link, $h_{e}\left(x\right)={∥p_{i}-q_{a}∥}_{2}$.

The propagation state is modeled by $c_{e}\in L=\left\{\mathrm{LOS},\mathrm{wLOS},\mathrm{NLOS}\right\}$, with $\sigma_{e}=\sigma_{c_{e}}$ and $b_{e}=\mu_{c_{e}}$. The class parameters satisfy $\sigma_{\mathrm{LOS}}<\sigma_{\mathrm{wLOS}}<\sigma_{\mathrm{NLOS}}$, $\mu_{\mathrm{LOS}}=\mu_{\mathrm{wLOS}}=0$, and $\mu_{\mathrm{NLOS}}\geq 0$. The heteroscedastic random-noise experiment uses $\mu_{\mathrm{NLOS}}=0$, whereas the NLOS-bias experiment uses $\mu_{\mathrm{NLOS}}=b_{\mathrm{NLOS}}>0$.

In practical operation, $c_{e}$, $\sigma_{e}^{2}$, and $b_{e}$ are not assumed known exactly. Link-quality features $f_{e}$ are used to obtain ${\hat{\pi }}_{e,c}=\mathrm{Pr}\left(c_{e}=c|f_{e}\right)$ with $\sum_{c\in L}{\hat{\pi }}_{e,c}=1$ and posterior-mean bias ${\hat{b}}_{e}=\sum_{c\in L}{\hat{\pi }}_{e,c}\mu_{c}$. After subtracting this mean, moment matching gives

(4) $${\hat{\sigma }}_{e,\mathrm{res}}^{2}=\sum_{c\in L}{\hat{\pi }}_{e,c}\left[\sigma_{c}^{2}+{\left(\mu_{c}-{\hat{b}}_{e}\right)}^{2}\right].$$

A hard classification simply makes $\left\{{\hat{\pi }}_{e,c}\right\}$ one-hot at ${\hat{c}}_{e}$.

For a selected node subset S, let $M_{S}=\left|E\left(S\right)\right|$. The actual measurement, estimated-bias, corrected-measurement, and residual-bias vectors are collected as

$$\begin{array}{cc} z_{S}=h_{S}\left(x\right)+b_{S}+n_{S},\; & {\hat{b}}_{S}={\left[{\hat{b}}_{e_{1}},\ldots ,{\hat{b}}_{e_{M_{S}}}\right]}^{T},\\ {\tilde{z}}_{S}=z_{S}-{\hat{b}}_{S}=h_{S}\left(x\right)+{\tilde{b}}_{S}+n_{S},\; & {\tilde{b}}_{S}=b_{S}-{\hat{b}}_{S}. \end{array}$$

The online covariance is ${\hat{R}}_{S}=\mathrm{diag}\left({\hat{\sigma }}_{e_{1},\mathrm{res}}^{2},\ldots ,{\hat{\sigma }}_{e_{M_{S}},\mathrm{res}}^{2}\right)$. Linearization at $\hat{x}$ gives

(5) $$\delta {\tilde{z}}_{S}\approx H_{S}\delta x+{\tilde{b}}_{S}+n_{S}.$$

Accurate posterior-mean correction makes ${\tilde{b}}_{S}$ small; without correction, ${\hat{b}}_{S}=0$ and the residual bias equals the injected physical bias.

For an unknown-node–unknown-node link, define $u_{ij}=\left({\hat{p}}_{i}-{\hat{p}}_{j}\right)/{∥{\hat{p}}_{i}-{\hat{p}}_{j}∥}_{2}$; the corresponding Jacobian row is $h_{ij}=\left[0,\ldots ,u_{ij}^{T},\ldots ,-u_{ij}^{T},\ldots ,0\right]$. For an unknown-node–anchor-node link, $u_{ia}=\left({\hat{p}}_{i}-q_{a}\right)/{∥{\hat{p}}_{i}-q_{a}∥}_{2}$, and only the position block of node *i* is nonzero.

Collecting all active rows gives $H_{S}\in R^{M_{S}\times 3N}$. With $R_{S}=\mathrm{diag}\left(\sigma_{e_{1}}^{2},\ldots ,\sigma_{e_{M_{S}}}^{2}\right)$, the physical Fisher information matrix is

(6) $$J_{\mathrm{phys}}\left(S\right)=H_{S}^{T}R_{S}^{-1}H_{S}+J_{\mathrm{prior}}.$$

Here $J_{\mathrm{prior}}⪰0$ contains only physically available prior information. In the anchor-free range-only analysis $J_{\mathrm{prior}}=0$, and $\hat{x}$ is only the linearization point. A reference variance $\sigma_{0}^{2}$ defines ${\bar{J}}_{\mathrm{prior}}=\sigma_{0}^{2}J_{\mathrm{prior}}$.

For oracle benchmarking, $w_{e}^{⋆}=\sigma_{0}^{2}/\sigma_{e}^{2}$ and $W_{S}^{⋆}=\sigma_{0}^{2}R_{S}^{-1}$, yielding

$${\bar{J}}_{\mathrm{phys}}^{⋆}\left(S\right)=H_{S}^{T}W_{S}^{⋆}H_{S}+{\bar{J}}_{\mathrm{prior}}=\sum_{e\in E(S)}w_{e}^{⋆}h_{e}^{T}h_{e}+{\bar{J}}_{\mathrm{prior}}.$$

These oracle quantities are used only as ideal simulation references.

In practical online weighting, Equation  (4) gives

(7) $${\hat{w}}_{e}=\frac{\sigma_{0}^{2}}{{\hat{\sigma }}_{e,\mathrm{res}}^{2}},$$

with ${\hat{W}}_{S}=\sigma_{0}^{2}{\hat{R}}_{S}^{-1}$ and

(8) $${\hat{\bar{J}}}_{\mathrm{phys}}\left(S\right)=H_{S}^{T}{\hat{W}}_{S}H_{S}+{\bar{J}}_{\mathrm{prior}}.$$

Equivalently, ${\hat{\bar{J}}}_{\mathrm{phys}}\left(S\right)=\sum_{e\in E(S)}{\hat{w}}_{e}h_{e}^{T}h_{e}+{\bar{J}}_{\mathrm{prior}}$.

Subsequent theoretical and algorithmic sections use $w_{e}\equiv {\hat{w}}_{e}$, $W_{S}\equiv {\hat{W}}_{S}$, and ${\bar{J}}_{\mathrm{phys}}\left(S\right)\equiv {\hat{\bar{J}}}_{\mathrm{phys}}\left(S\right)$. Thus ${\bar{J}}_{\mathrm{phys}}\left(S\right)=\sum_{e\in E(S)}w_{e}h_{e}^{T}h_{e}+{\bar{J}}_{\mathrm{prior}}$. Using Equation (2), the same model is $J_{\mathrm{phys}}\left(S\right)=J_{\mathrm{prior}}+\sum_{e\in E_{c}}\chi_{e}\left(S\right){\hat{w}}_{e}h_{e}^{T}h_{e}$. This separates fixed per-link information from subset-dependent link activation.

Unless ${\left(\cdot \right)}^{⋆}$ is shown, all weights and normalized information matrices are estimated online quantities. The covariance-based Fisher matrix does not explicitly represent deterministic residual NLOS bias, and $R_{S}$ and ${\hat{R}}_{S}$ serve different roles: the former describes physical random errors and oracle benchmarks, whereas the latter supplies practical online weights. Therefore, when ${\hat{R}}_{S}\neq R_{S}$, the inverse estimated information matrix is not generally the actual covariance of the mismatched WLS estimator; the corresponding first-order covariance and bias propagation are analyzed in Section First-Order Error Propagation Under Covariance and Bias Mismatch.

### 2.3. Gauge-Free Subspace Treatment for Anchor-Free Ranging

In an anchor-free range-only network, common translation and rotation leave all inter-node distances unchanged, so the full 3N-dimensional Jacobian and Fisher matrix contain rigid-body gauge directions and are singular. Center the predicted positions using ${\tilde{p}}_{i}={\hat{p}}_{i}-N^{-1}\sum_{j=1}^{N}{\hat{p}}_{j}$ and, for $p={\left[p_{x},p_{y},p_{z}\right]}^{T}$, define

$${\left[p\right]}_{\times }=\left[\begin{array}{ccc} 0 & -p_{z} & p_{y}\\ p_{z} & 0 & -p_{x}\\ -p_{y} & p_{x} & 0 \end{array}\right].$$

The translational and rotational gauge directions are then collected in

(9) $$G=\left[1_{N}⊗I_{3}\;\left[\begin{array}{c} -{\left[{\tilde{p}}_{1}\right]}_{\times }\\ -{\left[{\tilde{p}}_{2}\right]}_{\times }\\ \vdots \\ -{\left[{\tilde{p}}_{N}\right]}_{\times } \end{array}\right]\right]\in R^{3N\times 6}.$$

Let $G=U_{g}\sum_{g}V_{g}^{T}$ have singular values $\gamma_{1}\geq \gamma_{2}\geq \cdots \geq 0$. The numerical gauge rank and gauge-free dimension are

(10) $$d_{g}=\#\left\{\ell :\gamma_{\ell }>\tau_{g}\gamma_{1}\right\},\;\tau_{g}=10^{-10},$$

and

(11) $$d_{o}=3N-d_{g}.$$

Thus $d_{g}=6$ and $d_{o}=3N-6$ for a nondegenerate three-dimensional configuration, while degenerate configurations use the numerical rank directly. The gauge-free basis $Q_{o}=\mathrm{null}\left(G^{T}\right)\in R^{3N\times d_{o}}$ satisfies $Q_{o}^{T}Q_{o}=I_{d_{o}}$ and $Q_{o}^{T}G=0$. This basis removes only the rigid gauge; it does not itself guarantee complete local observability.

For the anchor-free range-only model, $H_{S}G=0$ and, when $J_{\mathrm{prior}}=0$, $J_{\mathrm{phys}}\left(S\right)G=0$. Projection onto the gauge-free coordinates gives

(12) $$J_{o}\left(S\right)=Q_{o}^{T}J_{\mathrm{phys}}\left(S\right)Q_{o}\in R^{d_{o}\times d_{o}}.$$

Disconnected, insufficiently constrained, or non-rigid topologies can still be rank deficient after this projection, so

$$r_{o}\left(S\right)=\#\left\{k:\lambda_{k}\left(J_{o}\left(S\right)\right)>\tau_{J}\lambda_{\max}\left(J_{o}\left(S\right)\right)\right\},\;\tau_{J}=10^{-8},$$

with $r_{o}\left(S\right)=0$ when $\lambda_{\max}\left(J_{o}\left(S\right)\right)=0$. Local observability modulo the rigid-body gauge therefore requires

(13) $$r_{o}\left(S\right)=d_{o}.$$

Equivalently, the symmetrized projected information matrix must be positive definite under the adopted relative eigenvalue threshold. Infeasible subsets are rejected rather than made invertible by artificial regularization. For fixed predicted geometry, G, $Q_{o}$, $d_{g}$, and $d_{o}$ are computed once and kept unchanged across candidate subsets.

#### First-Order Error Propagation Under Covariance and Bias Mismatch

The covariance-based Fisher matrix describes local random-error information, whereas deterministic residual NLOS bias must be propagated separately. Under covariance mismatch, the inverse of the estimated information matrix is also not generally the actual estimation-error covariance. Define

$$\begin{array}{cccc} H_{o,S} & =H_{S}Q_{o}, & R_{S} & =\mathrm{diag}(\sigma_{e_{1}}^{2},\ldots ,\sigma_{e_{M_{S}}}^{2}),\\ {\hat{R}}_{S} & =\mathrm{diag}({\hat{\sigma }}_{e_{1},\mathrm{res}}^{2},\ldots ,{\hat{\sigma }}_{e_{M_{S}},\mathrm{res}}^{2}), & J_{o,\mathrm{prior}} & =Q_{o}^{T}J_{\mathrm{prior}}Q_{o}. \end{array}$$

The oracle and practical projected matrices and their normalized forms are

$$\begin{array}{ccc} J_{o,\mathrm{true}}^{⋆}\left(S\right) & =H_{o,S}^{T}R_{S}^{-1}H_{o,S}+J_{o,\mathrm{prior}}, & \\ {\hat{J}}_{o,\mathrm{WLS}}\left(S\right) & =H_{o,S}^{T}{\hat{R}}_{S}^{-1}H_{o,S}+J_{o,\mathrm{prior}}, & \\ {\bar{J}}_{o}^{⋆}\left(S\right) & =\sigma_{0}^{2}J_{o,\mathrm{true}}^{⋆}\left(S\right),\; & {\hat{\bar{J}}}_{o}\left(S\right)=\sigma_{0}^{2}{\hat{J}}_{o,\mathrm{WLS}}\left(S\right). \end{array}$$

Unless ${\left(\cdot \right)}^{⋆}$ is shown, the subsequent pre-selection algorithm uses the normalized estimated quantity ${\hat{\bar{J}}}_{o}\left(S\right)$.

For a feasible topology, define ${\hat{A}}_{S}={\hat{J}}_{o,\mathrm{WLS}}{\left(S\right)}^{-1}H_{o,S}^{T}{\hat{R}}_{S}^{-1}$ and ${\tilde{b}}_{S}=b_{S}-{\hat{b}}_{S}$. For the anchor-free case with $J_{\mathrm{prior}}=0$, the first-order error and bias are

$$\delta {\hat{y}}_{S}\approx {\hat{A}}_{S}\left(n_{S}+{\tilde{b}}_{S}\right),\;\delta {\hat{y}}_{b}\left(S\right)={\hat{A}}_{S}{\tilde{b}}_{S}.$$

Since the physical random error has covariance $R_{S}$, the actual first-order random covariance is the sandwich expression

(14) $$C_{\mathrm{rand}}\left(S\right)={\hat{A}}_{S}R_{S}{\hat{A}}_{S}^{T}.$$

The dimension-normalized local MSE surrogate combines random and bias contributions,

$$\Psi_{\mathrm{local}}\left(S\right)=\frac{1}{d_{o}}\left\{\mathrm{tr}\left[C_{\mathrm{rand}}\left(S\right)\right]+{∥\delta {\hat{y}}_{b}\left(S\right)∥}_{2}^{2}\right\},$$

with RMSE surrogate

(15) $${\mathrm{RMSE}}_{\mathrm{local}}^{\mathrm{sur}}\left(S\right)=\sqrt{\Psi_{\mathrm{local}}\left(S\right)}.$$

The oracle gain is $A_{S}^{⋆}=J_{o,\mathrm{true}}^{⋆}{\left(S\right)}^{-1}H_{o,S}^{T}R_{S}^{-1}$. When ${\hat{R}}_{S}=R_{S}$, ${\tilde{b}}_{S}=0$, and $J_{\mathrm{prior}}=0$, $C_{\mathrm{rand}}\left(S\right)=J_{o,\mathrm{true}}^{⋆}{\left(S\right)}^{-1}$; otherwise $C_{\mathrm{rand}}\left(S\right)\neq {\hat{J}}_{o,\mathrm{WLS}}{\left(S\right)}^{-1}$ in general. Thus the inverse online information matrix must not be interpreted as the actual covariance or an oracle CRLB under model mismatch. The surrogate in Equation (15) remains a secondary diagnostic, whereas the primary NLOS-bias metric is the rigid-aligned nonlinear localization RMSE obtained directly from the biased UWB measurements.

### 2.4. Conventional GDOP Metric and Its Limitations

The geometric dilution of precision (GDOP) is a commonly used geometric performance metric in single-target localization. Under the assumption of independent and identically distributed measurement noise, the observation model can be expressed as

$$z=h\left(x\right)+n,\;n∼N\left(0,\sigma_{0}^{2}I\right).$$

Accordingly, the conventional GDOP can be written as

$$\mathrm{GDOP}=\sqrt{\mathrm{tr}\left[{\left(H^{T}H\right)}^{-1}\right]}.$$

In this case, the root mean square error (RMSE) of the estimator can be approximately expressed as

$$\mathrm{RMSE}\approx \sigma_{0}\cdot \mathrm{GDOP}.$$

The essence of conventional GDOP is to measure the amplification effect of observation geometry on measurement errors. It is a dimensionless metric with clear physical interpretation and low computational complexity. Although conventional GDOP has been widely used in single-target localization, satellite navigation, wireless positioning, and base-station layout problems, it has the following limitations when directly applied to cooperative ranging sensor networks.

First, conventional GDOP is based on the single-target localization assumption and is insufficient to characterize the error propagation mechanism caused by multi-node state coupling. In cooperative sensor network localization, the variables to be estimated consist of the positions of multiple mobile sensor nodes, and the state dimension is usually $3N_{u}$. UWB inter-node ranging observations introduce significant block-coupled structures into the error covariance matrix. Therefore, single-target GDOP is unable to reflect the system-level information quality of multi-node joint estimation.

Second, conventional GDOP cannot effectively handle heteroscedastic measurement noise. The conventional GDOP adopts the form of $H^{T}H$, which implicitly assumes that all observations follow the same noise variance model. In practical UAV-borne cooperative sensing systems, the accuracy of UWB ranging is affected by inter-node distance, occlusion, line-of-sight conditions, non-line-of-sight effects, and link quality. As a result, different ranging observations usually have different noise levels, and conventional GDOP cannot accurately reflect the differences in information contributions among different UWB links.

Third, conventional GDOP cannot fully characterize the influence of cooperative ranging sensor network topology on system-level localization performance. In cooperative localization systems, the information matrix is affected not only by local geometric relationships, but also by graph connectivity, information propagation paths, and the selection of cooperative sensor nodes or ranging links. Conventional GDOP mainly describes local geometric quality and is therefore insufficient to characterize the local observability and error-bound level of topology-coupled cooperative localization networks.

**Figure 1:**
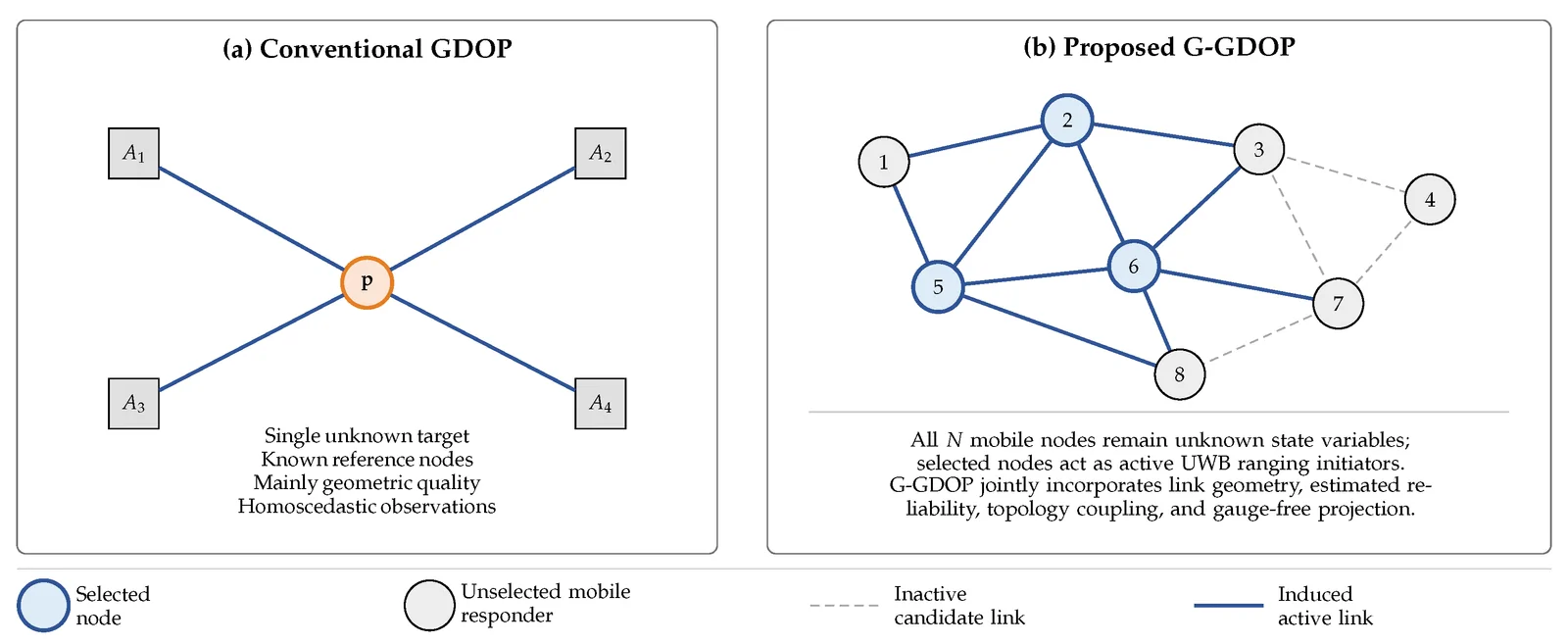
Conceptual comparison between conventional single-target GDOP and the proposed G-GDOP for anchor-free, topology-coupled multi-node UWB cooperative localization.

Figure 1 contrasts the conventional single-target GDOP setting with the proposed G-GDOP formulation. Conventional GDOP mainly describes the geometric quality of known reference-to-target observations. In the proposed anchor-free cooperative model, all mobile nodes remain unknown state variables, whereas only nodes in the selected node subset are scheduled as active UWB ranging initiators. The selected node subset induces the ranging topology, and G-GDOP evaluates its estimated local information quality after link-quality weighting and removal of the rigid-body gauge directions.

Generalized GDOP can be regarded as an extension of conventional GDOP from single-target homoscedastic localization to multi-node and heteroscedastic cooperative ranging sensor networks. At the application level, it extends the use of GDOP from geometric quality evaluation to system-level information assessment, cooperative sensor node pre-selection, and topology-aware cooperative localization. Therefore, it is necessary to construct a system-level generalized GDOP metric under the Fisher information matrix framework, so as to uniformly reflect the combined effects of link geometry, network topology, and ranging noise weights on the local information quality of cooperative localization systems.

### 2.5. Definition and Property Analysis of the Generalized GDOP Metric

In this paper, G-GDOP is defined within the FIM/CRLB framework for UWB-aided cooperative ranging sensor networks. It retains the error-amplification interpretation of conventional GDOP while incorporating link-quality-dependent noise weighting and multi-node topological coupling, thereby allowing local observability differences among cooperative ranging topologies to be compared.

#### 2.5.1. A-Optimal G-GDOP in the Gauge-Free Subspace

The trace-based A-optimal criterion is evaluated from the normalized Fisher information matrix projected onto the gauge-free subspace. Topologies that remain unobservable after removal of the rigid-body gauge are excluded by defining

(16) $$\Phi_{A}\left(S\right)=\left\{\begin{array}{cc} \mathrm{tr}\left[J_{o}{\left(S\right)}^{-1}\right],\; & r_{o}\left(S\right)=d_{o},\\ +\infty ,\; & r_{o}\left(S\right)<d_{o}, \end{array}\right.$$

with the corresponding generalized GDOP

(17) $$\mathrm{G-GDOP}\left(S\right)=\sqrt{\Phi_{A}\left(S\right)}.$$

For the practical pre-selection method, $J_{o}\left(S\right)$ is constructed from predicted geometry and estimated link-error covariance, so $\Phi_{A}\left(S\right)$ is an online information surrogate for ranking feasible subsets. Its inverse-trace value coincides with the normalized oracle CRLB only when the estimated and true covariances are identical; under covariance mismatch, the actual first-order estimator covariance is instead given by Equation (14).

For comparisons across network sizes, define

(18) $$\Phi_{A,\mathrm{norm}}\left(S\right)=\frac{1}{d_{o}}\Phi_{A}\left(S\right),$$

and

(19) $${\mathrm{G-GDOP}}_{\mathrm{norm}}\left(S\right)=\sqrt{\Phi_{A,\mathrm{norm}}\left(S\right)}.$$

For fixed network size and predicted geometry, $d_{o}$ is common to all feasible selected node subsets, and therefore

(20) $$\underset{S}{\arg\min}\;\Phi_{A}\left(S\right)=\underset{S}{\arg\min}\;\Phi_{A,\mathrm{norm}}\left(S\right).$$

The normalized form is used for cross-size reporting, whereas the unnormalized form is retained when discussing the connection with conventional GDOP.

#### 2.5.2. Physical Interpretation of the Generalized GDOP Metric

For a feasible topology induced by the selected node subset with $r_{o}\left(S\right)=d_{o}$, ${\bar{J}}_{o}^{⋆}{\left(S\right)}^{-1}$ is the normalized local oracle CRLB under the true random-error covariance. By contrast, the practical criterion uses ${\hat{\bar{J}}}_{o}\left(S\right)$, constructed from estimated link-error statistics, and its inverse trace is therefore an estimated local information-quality surrogate. A smaller practical G-GDOP indicates a more favorable estimated information structure under the adopted online geometry and weighting model, but does not by itself guarantee smaller nonlinear localization error under covariance mismatch, residual NLOS bias, or poor initialization.

The practical and oracle projected information models differ only in their link weights:

$$\begin{array}{cc} \;J_{o}\left(S\right)=Q_{o}^{T}\left[\sum_{e\in E(S)}{\hat{w}}_{e}h_{e}^{T}h_{e}+{\bar{J}}_{\mathrm{prior}}\right]Q_{o},\; & \;{\hat{w}}_{e}=\frac{\sigma_{0}^{2}}{{\hat{\sigma }}_{e,\mathrm{res}}^{2}},\\ J_{o}^{⋆}\left(S\right)=Q_{o}^{T}\left[\sum_{e\in E(S)}w_{e}^{⋆}h_{e}^{T}h_{e}+{\bar{J}}_{\mathrm{prior}}\right]Q_{o},\; & w_{e}^{⋆}=\frac{\sigma_{0}^{2}}{\sigma_{e}^{2}}. \end{array}$$

Thus G-GDOP jointly reflects link geometry, heterogeneous measurement reliability, and the position at which each link couples into the multi-node state. The link direction determines the spatial information direction, the variance determines its reliability, and the induced topology determines how that information couples the network state. This unified information representation is the main advantage of G-GDOP for heteroscedastic multi-node cooperative ranging.

#### 2.5.3. Property Analysis of the Generalized GDOP Criterion

The suitability of the proposed criterion for cooperative sensor node pre-selection is examined through monotonicity, spectral bounds, and sensitivity to ranging topology and link reliability in the gauge-free subspace.

##### Monotonicity

For fixed predicted geometry, estimated link weights, and gauge-free basis $Q_{o}$, let $S_{1}\subseteq S_{2}$. The endpoint-activation rule in Equation (2) implies the following complete ordering chain:

$$\begin{array}{cccc} \chi_{e}\left(S_{1}\right) & \leq \chi_{e}\left(S_{2}\right),\;\forall e\in E_{c}, & E(S_{1}) & \subseteq E(S_{2}),\\ \Delta J_{\mathrm{phys}} & =\sum_{e\in E\left(S_{2}\right)\setminus E\left(S_{1}\right)}{\hat{w}}_{e}h_{e}^{T}h_{e}⪰0,\; & \Delta J_{o} & =Q_{o}^{T}\Delta J_{\mathrm{phys}}Q_{o}⪰0,\\ {\bar{J}}_{o,2} & ={\bar{J}}_{o,1}+\Delta {\bar{J}}_{o}⪰{\bar{J}}_{o,1}, & {\bar{J}}_{o,2}^{-1} & ⪯{\bar{J}}_{o,1}^{-1},\\ \mathrm{tr}({\bar{J}}_{o,2}^{-1}) & \leq \mathrm{tr}({\bar{J}}_{o,1}^{-1}), & {\mathrm{G-GDOP}}_{2} & \leq {\mathrm{G-GDOP}}_{1},\\ & & {\mathrm{G-GDOP}}_{\mathrm{norm},2} & \leq {\mathrm{G-GDOP}}_{\mathrm{norm},1}. \end{array}$$

The inverse inequalities require both projected information matrices to be positive definite. Because $d_{o}=3N-d_{g}$ is fixed for the same network size and linearization geometry, adding valid ranging information cannot increase either A-optimal G-GDOP measure under these fixed conditions. The result does not compare different predicted geometries, because changing geometry requires recomputation of G, $d_{g}$, $d_{o}$, $Q_{o}$, and the ranging Jacobians.

##### Spectral Bounds

Let $0<\lambda_{\min}\left({\bar{J}}_{o}\right)\leq \lambda_{\max}\left({\bar{J}}_{o}\right)$ and $d_{o}=3N-d_{g}$; for the nondegenerate three-dimensional configurations used in the main experiments, $d_{g}=6$ and $d_{o}=3N-6$. The trace and both G-GDOP forms satisfy the compact set of bounds

$$\begin{array}{cc} \mathrm{tr}({\bar{J}}_{o}^{-1}) & =\sum_{k=1}^{d_{o}}\frac{1}{\lambda_{k}\left({\bar{J}}_{o}\right)},\\ \frac{d_{o}}{\lambda_{\max}\left({\bar{J}}_{o}\right)} & \leq \mathrm{tr}\left({\bar{J}}_{o}^{-1}\right)\leq \frac{d_{o}}{\lambda_{\min}\left({\bar{J}}_{o}\right)},\\ \sqrt{\frac{d_{o}}{\lambda_{\max}\left({\bar{J}}_{o}\right)}} & \leq \mathrm{G-GDOP}\leq \sqrt{\frac{d_{o}}{\lambda_{\min}\left({\bar{J}}_{o}\right)}},\\ \frac{1}{\sqrt{\lambda_{\max}\left({\bar{J}}_{o}\right)}} & \leq {\mathrm{G-GDOP}}_{\mathrm{norm}}\leq \frac{1}{\sqrt{\lambda_{\min}\left({\bar{J}}_{o}\right)}}. \end{array}$$

Hence all gauge-free eigen-directions influence G-GDOP, with particular sensitivity to weakly observed directions. As $\lambda_{\min}\left({\bar{J}}_{o}\right)\to 0$, conditioning deteriorates and the upper local error-amplification bound increases.

##### Sensitivity to Topology, Geometry, and Link Reliability

For a selected node subset S,

(21) $${\bar{J}}_{o}\left(S\right)=Q_{o}^{T}\left[\sum_{e\in E(S)}w_{e}h_{e}^{T}h_{e}+{\bar{J}}_{\mathrm{prior}}\right]Q_{o}.$$

In the anchor-free range-only experiments ${\bar{J}}_{\mathrm{prior}}=0$. Equation  (21) therefore exposes three coupled factors directly: E(S) determines the active topology, $h_{e}$ determines geometric direction and state coupling, and $w_{e}$ represents estimated UWB measurement reliability. Changing the selected node subset, an active link, its reliability weight, or the predicted geometry consequently changes ${\bar{J}}_{o}\left(S\right)$ and therefore G-GDOP.

In summary, G-GDOP is a trace-based A-optimal information measure evaluated in the gauge-free subspace and is finite only when the induced ranging topology is locally observable modulo the global rigid-body gauge. It extends conventional GDOP to topology-coupled, heteroscedastic cooperative ranging networks, while its validity remains local to the adopted predicted geometry and linearized measurement model. Dense networks may still exceed communication, sensing, and computational budgets; the next section therefore formulates the resource-constrained pre-selection problem and develops a marginal-gain-based greedy heuristic.

## 3. G-GDOP-Based Cooperative Sensor Node Pre-Selection

### 3.1. G-GDOP-Based Cooperative Sensor Node Pre-Selection Problem

In dense UWB-aided cooperative ranging sensor networks, bandwidth, ranging slots, energy, and onboard computation may prevent all mobile nodes from actively initiating ranging exchanges within one scheduling epoch. Let C={1,2,…,N} denote the complete mobile-node set and S⊆C the selected node subset. Under the fixed selected-node-budget case, |S|=K, while all *N* nodes remain in the localization state and unselected nodes may still serve as ranging responders.

As discussed in Section 2.5.1, the selection criterion is $\mathrm{G-GDOP}\left(S\right)=\sqrt{\Phi_{A}\left(S\right)}$, where for a feasible topology $r_{o}\left(S\right)=d_{o}$, $\Phi_{A}\left(S\right)=\mathrm{tr}\left[J_{o}{\left(S\right)}^{-1}\right]$. The basic fixed-budget problem is therefore

(22) $$\begin{array}{cc} \underset{S\subseteq C}{\mathrm{minimize}} & \mathrm{tr}\left[J_{o}{\left(S\right)}^{-1}\right]\\ \mathrm{subject}\mathrm{to} & |S|=K,\\ & r_{o}\left(S\right)=d_{o}. \end{array}$$

Because the selected node subset induces the active ranging-link set, a candidate node does not have a subset-independent information increment. Define the fixed projected prior and per-link contributions as

$$J_{o,\mathrm{base}}=Q_{o}^{T}J_{\mathrm{prior}}Q_{o},\;B_{e}={\hat{w}}_{e}Q_{o}^{T}h_{e}^{T}h_{e}Q_{o}⪰0.$$

All practical per-link matrices use estimated online weights; oracle weights are reserved for explicitly labeled benchmark and diagnostic evaluations. Using the activation indicator $\chi_{e}\left(S\right)$ gives

(23) $$J_{o}\left(S\right)=J_{o,\mathrm{base}}+\sum_{e\in E_{c}}\chi_{e}\left(S\right)B_{e}.$$

Equivalently, $J_{o}\left(S\right)=J_{o,\mathrm{base}}+\sum_{e\in E(S)}B_{e}$. For a current subset $S_{t}$ and candidate $i\notin S_{t}$, define

$$\Delta E_{i}\left(S_{t}\right)=E\left(S_{t}\cup \left\{i\right\}\right)\setminus E\left(S_{t}\right),\;\Delta J_{o,i}\left(S_{t}\right)=\sum_{e\in \Delta E_{i}\left(S_{t}\right)}B_{e}.$$

The candidate update is consequently $J_{o}\left(S_{t}\cup \left\{i\right\}\right)=J_{o}\left(S_{t}\right)+\Delta J_{o,i}\left(S_{t}\right)$, which makes its selection dependence explicit.

*Induced active-link number.* The selected-node budget controls selected nodes, not the number of ranging links generated by their induced topology. Define
  (24) $$M\left(S\right)=\left|E(S)\right|=\sum_{e\in E_{c}}\chi_{e}\left(S\right).$$
  For the complete candidate set, $M\left(C\right)=|E_{c}|$, whereas the number of links newly activated by candidate *i* is $\Delta M_{i}\left(S_{t}\right)=M\left(S_{t}\cup \left\{i\right\}\right)-M\left(S_{t}\right)=\left|\Delta E_{i}\left(S_{t}\right)\right|$. Thus the incremental link number equals the iteration-dependent update width before possible linear dependence among projected link vectors is considered.
*Ranging-slot occupation.* Let $s_{e}\in N_{+}$ denote the scheduling slots required by candidate link *e*. The total slot occupation is
  (25) $$T_{\mathrm{slot}}\left(S\right)=\sum_{e\in E_{c}}\chi_{e}\left(S\right)s_{e}=\sum_{e\in E(S)}s_{e}.$$
  Adding candidate *i* requires $\Delta T_{\mathrm{slot},i}\left(S_{t}\right)=\sum_{e\in \Delta E_{i}\left(S_{t}\right)}s_{e}$. When $s_{e}=1$ for every candidate link, $T_{\mathrm{slot}}\left(S\right)=M\left(S\right)$; the general definition also accommodates repeated ranging, handshakes, or multi-slot protocols.

#### 3.1.1. General Sensing-Resource Cost

Let $c_{i}^{\mathrm{node}}\geq 0$ denote the normalized cost of selecting node *i* as an active UWB ranging initiator and $c_{e}^{\mathrm{link}}\geq 0$ the normalized communication, ranging, energy, or processing cost associated with active link *e*. The total cost is

(26) $$C_{\mathrm{res}}\left(S\right)=\sum_{i\in S}c_{i}^{\mathrm{node}}+\sum_{e\in E_{c}}\chi_{e}\left(S\right)c_{e}^{\mathrm{link}}.$$

Equivalently, $C_{\mathrm{res}}\left(S\right)=\sum_{i\in S}c_{i}^{\mathrm{node}}+\sum_{e\in E(S)}c_{e}^{\mathrm{link}}$, and the incremental cost of candidate *i* is $\Delta C_{\mathrm{res},i}\left(S_{t}\right)=c_{i}^{\mathrm{node}}+\sum_{e\in \Delta E_{i}\left(S_{t}\right)}c_{e}^{\mathrm{link}}$. The node and link coefficients must use a common normalized resource unit. If hardware-specific energy or communication measurements are unavailable, |S|, M(S), and $T_{\mathrm{slot}}\left(S\right)$ are reported as the primary physical resource indicators rather than interpreting $C_{\mathrm{res}}$ as exact energy.

For the anchor-free range-only observability analysis, $J_{\mathrm{prior}}=0$ and $J_{o,\mathrm{base}}=0$; inverse-based A-optimal candidate evaluation is therefore performed only after a full-rank observable seed has been constructed. Beyond the selected-node budget and observability requirement, communication availability and residual energy can impose additional feasibility conditions.

#### 3.1.2. Communication-Availability Constraint

A candidate link belongs to $E_{c}$ only when its inter-node distance satisfies

(27) $$d_{ij}\leq d_{\max},\;\forall \left(i,j\right)\in E_{c}.$$

Hence $E\left(S\right)\subseteq E_{c}$ automatically, and this range condition is enforced during candidate-graph construction rather than repeated as a selection-dependent constraint in Equation (29).

#### 3.1.3. Energy-Feasibility Constraint

A selected node must satisfy

(28) $$E_{i}\geq E_{\min},\;\forall i\in S,$$

where $E_{i}$ is the residual energy of node *i* and $E_{\min}$ the minimum threshold for initiating ranging. This is an eligibility condition on an individual node, whereas $C_{\mathrm{res}}\left(S\right)$ is an aggregate per-epoch cost of the selected node subset and its induced topology.

#### 3.1.4. Local-Observability Constraint

The projected information matrix must reach the complete gauge-free dimension, $r_{o}\left(S\right)=d_{o}$, which is equivalent under the adopted numerical threshold to requiring the symmetrized $J_{o}\left(S\right)$ to be positive definite. Combining the resource and observability requirements gives

(29) $$\begin{array}{cc} \underset{S\subseteq C}{\mathrm{minimize}} & \Phi_{A}\left(S\right)=\mathrm{tr}\left[J_{o}{\left(S\right)}^{-1}\right]\\ \mathrm{subject}\mathrm{to} & |S|\leq K,\\ & M\left(S\right)\leq B_{\mathrm{link}},\\ & T_{\mathrm{slot}}\left(S\right)\leq B_{\mathrm{slot}},\\ & C_{\mathrm{res}}\left(S\right)\leq B_{\mathrm{res}},\\ & E_{i}\geq E_{\min},\;\forall i\in S,\\ & r_{o}\left(S\right)=d_{o}. \end{array}$$

Here *K*, $B_{\mathrm{link}}$, $B_{\mathrm{slot}}$, and $B_{\mathrm{res}}$ are respectively the node, induced-link, ranging-slot, and general-resource budgets. The candidate graph has already been formed under

$$G_{c}=\left(C,E_{c}\right),\;E\left(S\right)\subseteq E_{c},\;d_{ij}\leq d_{\max}\;\forall \left(i,j\right)\in E_{c},$$

so these are graph properties rather than additional binary selection constraints. Fixed-*K* experiments use sufficiently loose link, slot, and general-resource budgets and impose |S|=K; the complete resource-constrained formulation uses |S|≤K because another budget may become active first.

#### 3.1.5. Information–Resource Trade-Off Indicators

Let $S_{\mathrm{all}}=C$ denote the All-Node Reference. If its induced topology is locally observable, define the quality-retention, resource-usage, and saving ratios by

$$\begin{array}{cc} \;Q_{A}\left(S\right)=\frac{\Phi_{A}\left(S_{\mathrm{all}}\right)}{\Phi_{A}\left(S\right)},\; & 0<Q_{A}\left(S\right)\leq 1,\\ \rho_{\mathrm{link}}\left(S\right)=\frac{M(S)}{|E_{c}|},\; & \;\rho_{\mathrm{slot}}\left(S\right)=\frac{T_{\mathrm{slot}}\left(S\right)}{T_{\mathrm{slot}}\left(S_{\mathrm{all}}\right)},\\ \;\rho_{\mathrm{res}}\left(S\right)=\frac{C_{\mathrm{res}}\left(S\right)}{C_{\mathrm{res}}\left(S_{\mathrm{all}}\right)},\; & \;s_{q}\left(S\right)=1-\rho_{q}\left(S\right),\;q\in \left\{\mathrm{link},\mathrm{slot},\mathrm{res}\right\}. \end{array}$$

A value of $Q_{A}$ closer to one indicates greater retention of All-Node A-optimal information quality. A feasible solution $S_{a}$ dominates $S_{b}$ in the information–resource plane when

$$\Phi_{A}\left(S_{a}\right)\leq \Phi_{A}\left(S_{b}\right),\;C_{\mathrm{res}}\left(S_{a}\right)\leq C_{\mathrm{res}}\left(S_{b}\right),$$

with at least one strict inequality; the nondominated solutions form an empirical information–resource Pareto frontier.

The resulting optimization remains combinatorial: for fixed cardinality there are NK possible subsets. This growth makes exhaustive search suitable only for small-scale optimality verification and impractical for online dense-network pre-selection, motivating the marginal-gain greedy heuristic introduced next.

**Figure 2:**
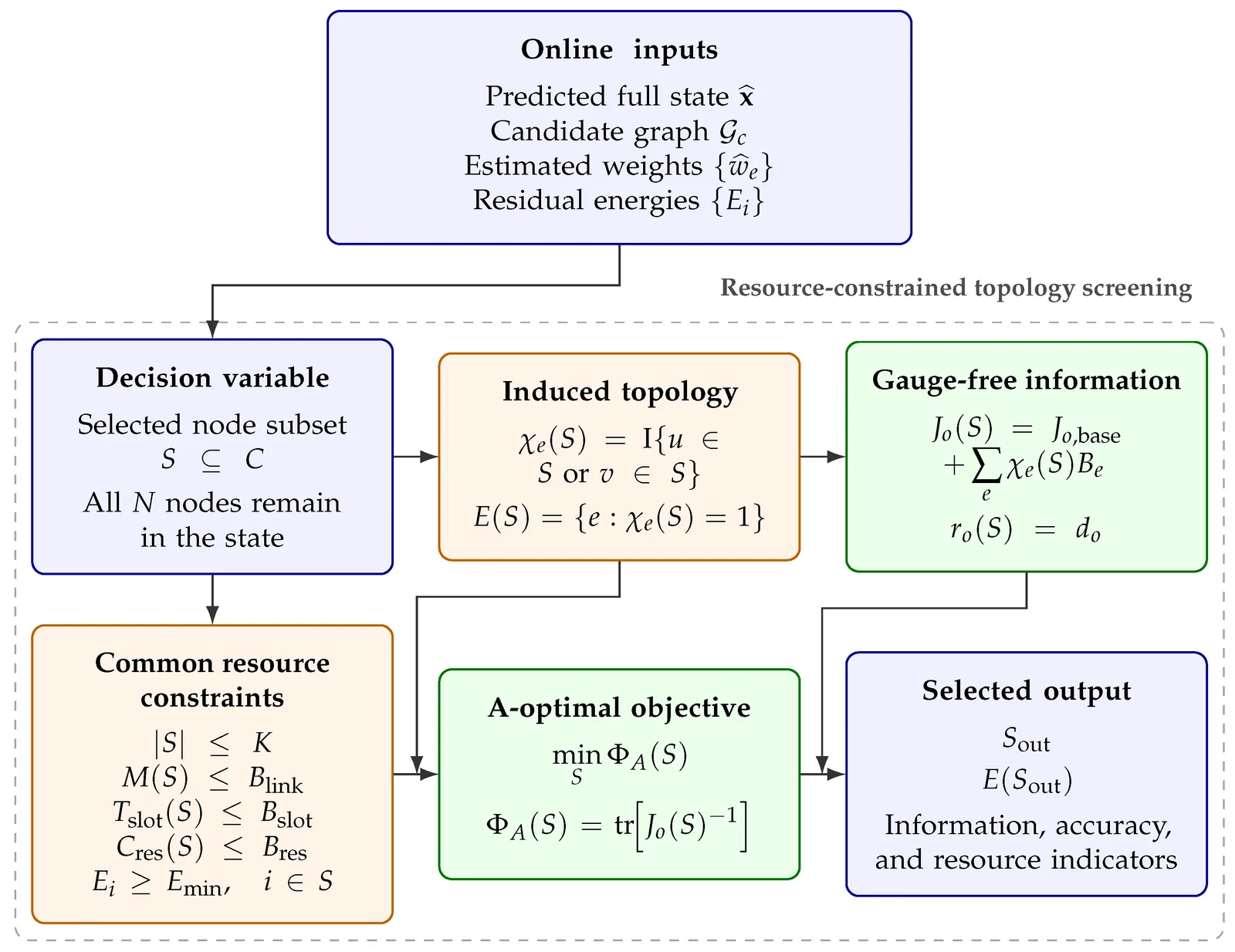
Resource-constrained cooperative sensor node pre-selection problem with induced ranging links and gauge-free information evaluation. Arrows indicate the dependency flow from the online inputs and selection decision to the induced topology, information matrix, feasibility constraints, objective, and final selected-node output.

Figure 2 summarizes the resource-constrained formulation. The candidate graph, predicted geometry, and estimated link-quality statistics form the inputs, and the selected node subset is the decision variable. Active ranging links are induced by the endpoint-activation rule. Feasible selected node subsets are evaluated through the gauge-free projected information matrix under the common node, link, slot, energy, resource-cost, and local-observability constraints.

### 3.2. Greedy Cooperative Sensor Node Pre-Selection Heuristic Based on G-GDOP Marginal Gain

The method consists of resource-feasible observable-seed construction followed by post-seed A-optimal selection. Let $S_{t}\subseteq C$ denote the selected node subset after iteration *t*. Both stages use

$$\begin{array}{cc} F_{t}=\{i\in C\setminus S_{t}\;|\; & E_{i}\geq E_{\min},\\ & M\left(S_{t}\right)+\Delta M_{i}\left(S_{t}\right)\leq B_{\mathrm{link}},\\ & T_{\mathrm{slot}}\left(S_{t}\right)+\Delta T_{\mathrm{slot},i}\left(S_{t}\right)\leq B_{\mathrm{slot}},\\ & C_{\mathrm{res}}\left(S_{t}\right)+\Delta C_{\mathrm{res},i}\left(S_{t}\right)\leq B_{\mathrm{res}}\}. \end{array}$$

Algorithm 1 gives the stage-specific evaluation, stopping, and failure logic.
**Algorithm 1:** Greedy Cooperative Sensor Node Pre-Selection Heuristic Based on G-GDOP    **Input:** *C*, $G_{C}=\left(C,E_{C}\right)$, *K*, $B_{\mathrm{link}}$, $B_{\mathrm{slot}}$, $B_{\mathrm{res}}$, $E_{\min}$, $\left\{E_{i}\right\}$, $\left\{s_{e}\right\}$, $\left\{c_{i}^{\mathrm{node}}\right\}$, $\left\{c_{e}^{\mathrm{link}}\right\}$, $\hat{x}$, $\left\{{\hat{w}}_{e}\right\}$, $\tau_{J}$, and $\epsilon_{\mathrm{tie}}$; use +∞ to disable an upper budget.     **Output:** status, $S_{\mathrm{out}}$, $E(S_{\mathrm{out}})$, *M*, $T_{\mathrm{slot}}$, and $C_{\mathrm{res}}$.
  1: Compute $Q_{o}$, $d_{g}$, $d_{o}$, and the projected weighted link-information vectors ${\left\{a_{e}\right\}}_{e\in E_{C}}$.   2:  **if** preprocessing fails **then return** SELECTION_NUMERICAL_FAILURE.   3:  S←⌀; E←⌀; M←0; $T_{\mathrm{slot}}\leftarrow 0$; $C_{\mathrm{res}}\leftarrow 0$; $J_{o}\leftarrow 0$.   4:  **function** $S_{\mathrm{TATS}}(i,S)$.   5:    $\Delta E_{i}\leftarrow E\left(S\cup \left\{i\right\}\right)\setminus E\left(S\right)$.   6:    $\Delta M_{i}\leftarrow \left|\Delta E_{i}\right|$; $\Delta T_{i}\leftarrow \sum_{e\in \Delta E_{i}}s_{e}$.   7:    $\Delta C_{i}\leftarrow c_{i}^{\mathrm{node}}+\sum_{e\in \Delta E_{i}}c_{e}^{\mathrm{link}}$.   8:    $\Delta J_{o,i}\leftarrow \sum_{e\in \Delta E_{i}}a_{e}a_{e}^{T}$.   9:    **return** $\left(\Delta E_{i},\Delta M_{i},\Delta T_{i},\Delta C_{i},\Delta J_{o,i}\right)$. 10:  **end function**. 11:  **function** $F_{\mathrm{EASIBLE}}(i,\Delta M_{i},\Delta T_{i},\Delta C_{i})$. 12:    **return** $\left(E_{i}\geq E_{\min}\right)\wedge \left(|S|+1\leq K\right)\wedge \left(M+\Delta M_{i}\leq B_{\mathrm{link}}\right)\wedge \left(T_{\mathrm{slot}}+\Delta T_{i}\leq B_{\mathrm{slot}}\right)\wedge \left(C_{\mathrm{res}}+\Delta C_{i}\leq B_{\mathrm{res}}\right)$. 13:  **end function**.        *Stage I: observable-seed construction*
14:  **while** ${\mathrm{rank}}_{\tau_{J}}\left(J_{o}\right)<d_{o}$ **do**. 15:    **if** |S|≥K **then return** UNOBSERVABLE. 16:    $C_{\mathrm{rem}}\leftarrow C\setminus S$. 17:    **if** $C_{\mathrm{rem}}=⌀$ **then return** UNOBSERVABLE. 18:    $F_{\mathrm{seed}}\leftarrow ⌀$; $i^{⋆}\leftarrow ⌀$; $\kappa^{⋆}\leftarrow ⌀$; $r_{\mathrm{cur}}\leftarrow {\mathrm{rank}}_{\tau_{J}}\left(J_{o}\right)$. 19:    **for each** $i\in C_{\mathrm{rem}}$ in increasing node-ID order **do**. 20:         $\left(\Delta E_{i},\Delta M_{i},\Delta T_{i},\Delta C_{i},\Delta J_{o,i}\right)\leftarrow S_{\mathrm{TATS}}\left(i,S\right)$. 21:         **if** $\neg F_{\mathrm{EASIBLE}}(i,\Delta M_{i},\Delta T_{i},\Delta C_{i})$ **then continue**. 22:         $F_{\mathrm{seed}}\leftarrow F_{\mathrm{seed}}\cup \left\{i\right\}$; $J_{o}^{+i}\leftarrow J_{o}+\Delta J_{o,i}$. 23:         $r_{i}\leftarrow {\mathrm{rank}}_{\tau_{J}}\left(J_{o}^{+i}\right)$; compute the retained $\lambda_{\min}^{+i}$ of $J_{o}^{+i}$. 24:         $\kappa_{i}\leftarrow \left(r_{i}-r_{\mathrm{cur}},\lambda_{\min}^{+i},-\Delta T_{i},-\Delta C_{i},-i\right)$. 25:         **if** $i^{⋆}=⌀$ **or** $\kappa_{i}{≻}_{\mathrm{lex}}\kappa^{⋆}$ **then** $i^{⋆}\leftarrow i$ and $\kappa^{⋆}\leftarrow \kappa_{i}$. 26:    **end for**. 27:    **if** $F_{\mathrm{seed}}=⌀$ **then return** RESOURCE_INFEASIBLE. 28:    $\left(\Delta E_{i^{⋆}},\Delta M_{i^{⋆}},\Delta T_{i^{⋆}},\Delta C_{i^{⋆}},\Delta J_{o,i^{⋆}}\right)\leftarrow S_{\mathrm{TATS}}\left(i^{⋆},S\right)$; $S\leftarrow S\cup \{i^{⋆}\}$; $E\leftarrow E\cup \Delta E_{i^{⋆}}$. 29:    $M\leftarrow M+\Delta M_{i^{⋆}}$; $T_{\mathrm{slot}}\leftarrow T_{\mathrm{slot}}+\Delta T_{i^{⋆}}$; $C_{\mathrm{res}}\leftarrow C_{\mathrm{res}}+\Delta C_{i^{⋆}}$; $J_{o}\leftarrow J_{o}+\Delta J_{o,i^{⋆}}$. 30:  **end while**. 31:  $S_{\mathrm{seed}}\leftarrow S$; $k_{\mathrm{seed}}\leftarrow \left|S_{\mathrm{seed}}\right|$. 32:  **if** $J_{o}^{-1}$ cannot be evaluated **then return** SELECTION_NUMERICAL_FAILURE. 33:  $P\leftarrow J_{o}^{-1}$.        *Stage II: post-seed A-optimal refinement*
34:  **while** |S|<K **do**. 35:    $i^{⋆}\leftarrow ⌀$; $g^{⋆}\leftarrow -\infty$; $\Delta T^{⋆}\leftarrow +\infty$; $\Delta C^{⋆}\leftarrow +\infty$; $P^{⋆}\leftarrow P$. 36:    **for each** i∈C∖S in increasing node-ID order **do**. 37:         $\left(\Delta E_{i},\Delta M_{i},\Delta T_{i},\Delta C_{i},\Delta J_{o,i}\right)\leftarrow S_{\mathrm{TATS}}\left(i,S\right)$. 38:         **if** $\neg F_{\mathrm{EASIBLE}}(i,\Delta M_{i},\Delta T_{i},\Delta C_{i})$ **then continue**. 39:         **if** $\Delta E_{i}=⌀$ **then** $P^{\left(i\right)}\leftarrow P$; **else** compute $P^{\left(i\right)}$ using Equation (37). 40:         $g_{i}\leftarrow \mathrm{tr}\left(P\right)-\mathrm{tr}\left(P^{\left(i\right)}\right)$. 41:         **if** $i^{⋆}=⌀$ **or** $g_{i}>g^{⋆}+\epsilon_{\mathrm{tie}}$ **then** store $\left(i,g_{i},\Delta T_{i},\Delta C_{i},P^{\left(i\right)}\right)$ as the current candidate. 42:         **else if** $|g_{i}-g^{⋆}|\leq \epsilon_{\mathrm{tie}}$ **and** $\Delta T_{i}<\Delta T^{⋆}-\epsilon_{\mathrm{tie}}$ **then** store $\left(i,g_{i},\Delta T_{i},\Delta C_{i},P^{\left(i\right)}\right)$. 43:         **else if** $|g_{i}-g^{⋆}|\leq \epsilon_{\mathrm{tie}}$, $|\Delta T_{i}-\Delta T^{⋆}|\leq \epsilon_{\mathrm{tie}}$, and $\Delta C_{i}<\Delta C^{⋆}-\epsilon_{\mathrm{tie}}$ **then** store $\left(i,g_{i},\Delta T_{i},\Delta C_{i},P^{\left(i\right)}\right)$. 44:         **else if** $|g_{i}-g^{⋆}|\leq \epsilon_{\mathrm{tie}}$, $|\Delta T_{i}-\Delta T^{⋆}|\leq \epsilon_{\mathrm{tie}}$, $|\Delta C_{i}-\Delta C^{⋆}|\leq \epsilon_{\mathrm{tie}}$, and $i<i^{⋆}$ **then** store $\left(i,g_{i},\Delta T_{i},\Delta C_{i},P^{\left(i\right)}\right)$. 45:    **end for**. 46:    **if** $i^{⋆}=⌀$ **then break**. 47:    $\left(\Delta E_{i^{⋆}},\Delta M_{i^{⋆}},\Delta T_{i^{⋆}},\Delta C_{i^{⋆}},\Delta J_{o,i^{⋆}}\right)\leftarrow S_{\mathrm{TATS}}\left(i^{⋆},S\right)$. 48:    $S\leftarrow S\cup \{i^{⋆}\}$; $E\leftarrow E\cup \Delta E_{i^{⋆}}$. 49:    $M\leftarrow M+\Delta M_{i^{⋆}}$; $T_{\mathrm{slot}}\leftarrow T_{\mathrm{slot}}+\Delta T_{i^{⋆}}$; $C_{\mathrm{res}}\leftarrow C_{\mathrm{res}}+\Delta C_{i^{⋆}}$. 50:    $J_{o}\leftarrow J_{o}+\Delta J_{o,i^{⋆}}$; $P\leftarrow P^{⋆}$. 51:  **end while**. 52:  **if** exact-*K* selection is required **and** |S|<K **then return** RESOURCE_INFEASIBLE. 53:  **if** ${\mathrm{rank}}_{\tau_{J}}\left(J_{o}\right)<d_{o}$ **then return** UNOBSERVABLE. 54:  Evaluate the final A-optimal metric and resource statistics. 55:  **if** the final metric evaluation fails **then return** SELECTION_NUMERICAL_FAILURE. 56:  $S_{\mathrm{out}}\leftarrow S$. 57:  **return** $\mathrm{SUCCESS},S_{\mathrm{out}},E\left(S_{\mathrm{out}}\right),M,T_{\mathrm{slot}},C_{\mathrm{res}}$.


After a full-rank seed exists, the marginal gain for $i\in F_{t}$ is

(30) $$\Delta \left(i|S_{t}\right)=\Phi_{A}\left(S_{t}\right)-\Phi_{A}\left(S_{t}\cup \left\{i\right\}\right).$$

Its dependence on $S_{t}$ reflects the fact that some links incident to *i* may already have been activated. The greedy rule is

(31) $$i^{⋆}=\underset{i\in F_{t}}{\arg\max}\;\Delta \left(i|S_{t}\right),$$

with numerically tied gains resolved first by smaller $\Delta T_{\mathrm{slot},i}\left(S_{t}\right)$ and then by smaller $\Delta C_{\mathrm{res},i}\left(S_{t}\right)$. The subset update is simply $S_{t+1}=S_{t}\cup \left\{i^{⋆}\right\}$.

Because an empty anchor-free network is rank deficient, Stage I first constructs $S_{\mathrm{seed}}$ satisfying

(32) $$r_{o}\left(S_{\mathrm{seed}}\right)=d_{o}.$$

Writing $k_{\mathrm{seed}}=\left|S_{\mathrm{seed}}\right|$, if $k_{\mathrm{seed}}=K$ the node budget is already exhausted and the returned selected node subset is determined entirely by the Stage I lexicographic observable-seed ordering. Otherwise, Stage II starts from $S_{k_{\mathrm{seed}}}=S_{\mathrm{seed}}$ and $P_{k_{\mathrm{seed}}}={\bar{J}}_{o}{\left(S_{\mathrm{seed}}\right)}^{-1}$. The detailed ordering, resource checks, and failure returns remain those in Algorithm 1.

#### 3.2.1. Set-Dependent Induced-Link Information Increment

For $i\in F_{t}$, the newly activated link set is

(33) $$\Delta E_{i}\left(S_{t}\right)=E\left(S_{t}\cup \left\{i\right\}\right)\setminus E\left(S_{t}\right).$$

This set difference prevents double counting under endpoint activation. The associated projected increment is

(34) $$\Delta J_{o,i}\left(S_{t}\right)=\sum_{e\in \Delta E_{i}\left(S_{t}\right)}B_{e},$$

equivalently $\Delta J_{o,i}\left(S_{t}\right)=\sum_{e\in \Delta E_{i}\left(S_{t}\right)}{\hat{w}}_{e}Q_{o}^{T}h_{e}^{T}h_{e}Q_{o}$. Hence $J_{o}\left(S_{t}\cup \left\{i\right\}\right)=J_{o}\left(S_{t}\right)+\Delta J_{o,i}\left(S_{t}\right)$. Both the increment and marginal gain must therefore be reconstructed at every iteration. If $\Delta E_{i}\left(S_{t}\right)=⌀$, then $\Delta J_{o,i}\left(S_{t}\right)=0$ and $\Delta (i|S_{t})=0$. After selecting $i^{⋆}$, $E_{t+1}=E_{t}\cup \Delta E_{i^{⋆}}\left(S_{t}\right)$ with $E_{t}=E\left(S_{t}\right)$.

#### 3.2.2. Low-Rank Recursive Inverse Update

Candidate evaluation uses the estimated weight ${\hat{w}}_{e}$ from Equation (7). For each newly activated link define $a_{e}=\sqrt{w_{e}}Q_{o}^{T}h_{e}^{T}\in R^{d_{o}}$, and collect the $r_{i,t}=\left|\Delta E_{i}\left(S_{t}\right)\right|$ vectors in $U_{i}\left(S_{t}\right)=\left[a_{e_{1}},\ldots ,a_{e_{r_{i,t}}}\right]\in R^{d_{o}\times r_{i,t}}$. The low-rank information increment is

(35) $$\Delta {\bar{J}}_{o,i}\left(S_{t}\right)=U_{i}\left(S_{t}\right)U_{i}{\left(S_{t}\right)}^{T}.$$

With current inverse

(36) $$P_{t}={\bar{J}}_{o}{\left(S_{t}\right)}^{-1},$$

the candidate inverse is obtained from

(37) $$\begin{array}{cc} P_{t}^{\left(i\right)}= & P_{t}-P_{t}U_{i}\left(S_{t}\right)\\ & \times {\left[I_{r_{i,t}}+U_{i}{\left(S_{t}\right)}^{T}P_{t}U_{i}\left(S_{t}\right)\right]}^{-1}\\ & \times U_{i}{\left(S_{t}\right)}^{T}P_{t}. \end{array}$$

After $i^{⋆}$ is selected, ${\bar{J}}_{o}\left(S_{t+1}\right)={\bar{J}}_{o}\left(S_{t}\right)+\Delta {\bar{J}}_{o,i^{⋆}}\left(S_{t}\right)$ and

(38) $$P_{t+1}=P_{t}^{\left(i^{⋆}\right)}.$$

All candidate matrices remain in the same $d_{o}=3N-d_{g}$ gauge-free subspace for fixed predicted geometry, so the Woodbury update is dimensionally consistent after observable-seed construction. For the nondegenerate three-dimensional experiments, $d_{g}=6$ and $d_{o}=3N-6$.

**Figure 3:**
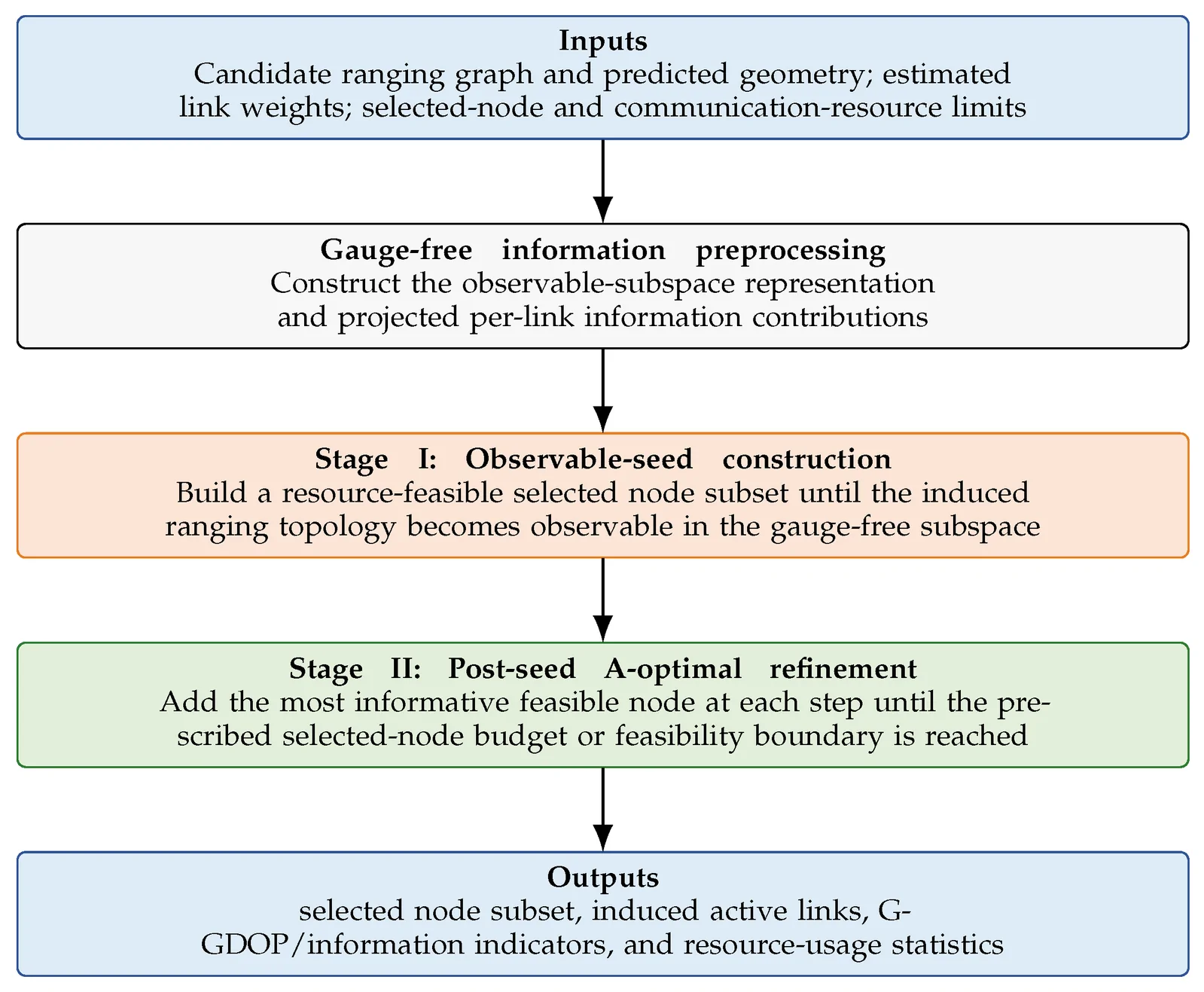
High-level architecture of the proposed two-stage cooperative sensor node pre-selection framework. Algorithm 1 provides the executable candidate-screening, update, tie-breaking, and failure logic, whereas Figure 4 illustrates the corresponding induced-topology evolution.

**Figure 4:**
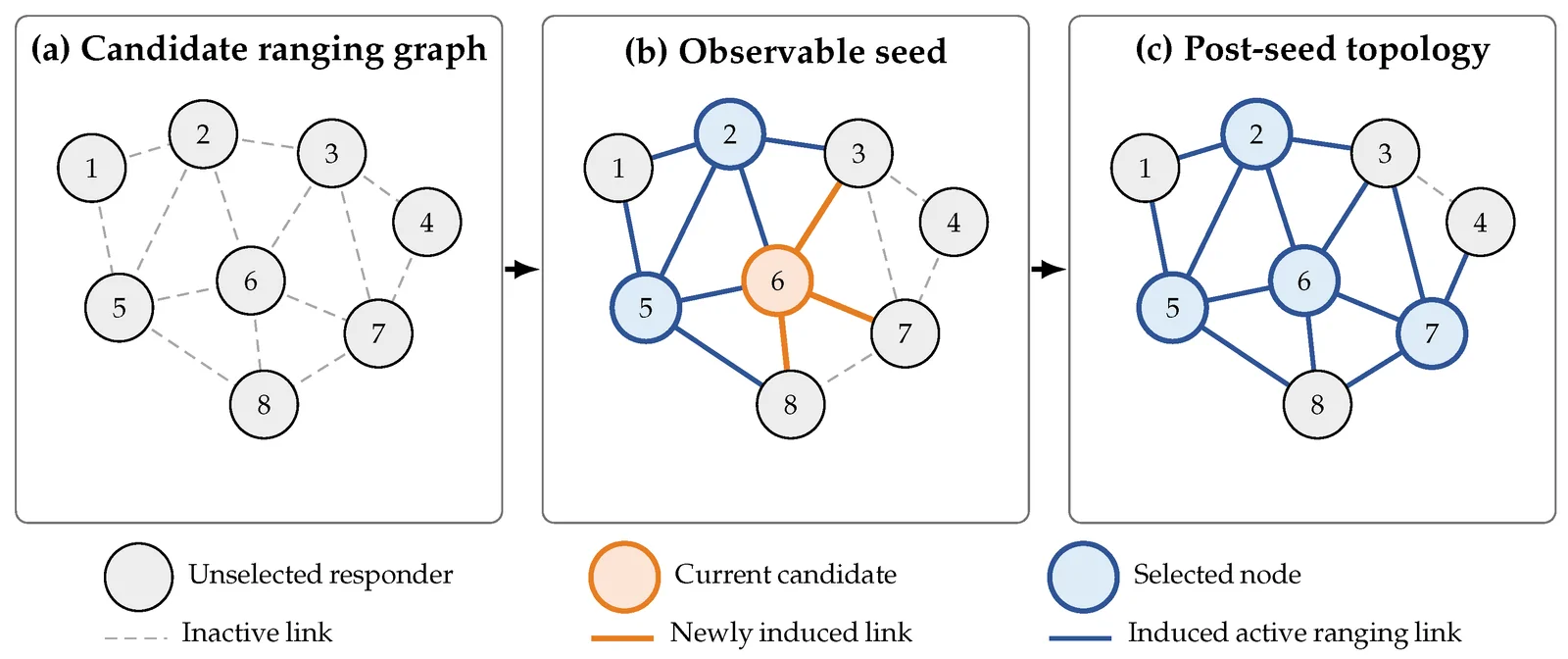
Illustration of induced-topology evolution from the candidate ranging graph to an observable seed and subsequent post-seed refinement. Colors distinguish selected nodes, unselected mobile nodes, candidate links, induced active ranging links, and the links newly induced by the highlighted candidate; the detailed decision logic is given in Algorithm 1. The numbers denote mobile-node IDs.

Algorithm 1 provides the executable selection procedure, Figure 3 summarizes the high-level two-stage workflow, and Figure 4 gives a concise structural illustration of the corresponding induced-topology evolution.

The proposed procedure is treated as a computationally efficient greedy heuristic for engineering implementation. For the general optimization problem

$$\min_{\begin{array}{c} S\subseteq C\\ |S|=K \end{array}}\mathrm{tr}\left[{\bar{J}}_{o}{\left(S\right)}^{-1}\right].$$

the inverse-trace A-optimal objective is not generally submodular without additional structural assumptions. In particular, the information contribution of a candidate node may depend on the currently selected node subset and the corresponding induced ranging topology. Therefore, no general constant-factor approximation guarantee is claimed for the proposed method. Instead, the solution quality of the proposed greedy heuristic is evaluated empirically through repeated exhaustive-search comparisons in small-scale networks and paired Monte Carlo experiments under different operating conditions.

For the following complexity analysis, the candidate graph $G_{c}=\left(C,E_{c}\right)$ is represented using adjacency lists together with a Boolean active-link mask. The projected weighted information vector $a_{e}\in R^{d_{o}}$ is precomputed for each candidate ranging link $e\in E_{c}$.

Candidate nodes are evaluated sequentially in the complexity bounds reported below. Parallel candidate evaluation is possible within one greedy iteration, but its additional synchronization and memory costs are not included in the serial bounds.

### 3.3. Algorithm Complexity and Scalability Analysis

The complexity depends on the candidate-node count, graph density, gauge-free dimension, update width, and resource-screening cost. The main quantities are N=|C|, $M_{c}=\left|E_{c}\right|$, $d_{o}=3N-d_{g}$, $k_{\mathrm{seed}}=\left|S_{\mathrm{seed}}\right|$, and $K_{\mathrm{out}}=\left|S_{\mathrm{out}}\right|\leq K$. Under a fixed-*K* experiment with loose resource budgets, $K_{\mathrm{out}}=K$; otherwise a resource constraint may cause $K_{\mathrm{out}}<K$. At iteration *t*, $F_{t}\subseteq C\setminus S_{t}$ is the resource-feasible candidate set, $n_{t}=\left|F_{t}\right|$ is the number requiring method-specific evaluation, and $r_{i,t}=\left|\Delta E_{i}\left(S_{t}\right)\right|$ is the number of newly activated links for candidate *i*. Define $r_{\max}=\max_{t,i}r_{i,t}$. Since $r_{i,t}$ is the number of columns of $U_{i}\left(S_{t}\right)$, the algebraic rank of an information increment is at most $r_{i,t}$.

#### 3.3.1. Preprocessing Complexity

Preprocessing consists of candidate-graph construction, gauge-free basis construction, and precomputation of projected per-link vectors. Direct pairwise graph construction costs $O(N^{2})$, although this term is replaced by the corresponding graph-construction cost when the graph is supplied externally or obtained through spatial indexing. With $G\in R^{3N\times d_{g}}$, $Q_{o}\in R^{3N\times d_{o}}$, and $d_{g}\leq 6$, constructing the explicit orthogonal complement requires approximately $O(Nd_{o}d_{g})$ operations. Each projected vector

$$a_{e}=\sqrt{{\hat{w}}_{e}}\;Q_{o}^{T}h_{e}^{T}\in R^{d_{o}}$$

can be formed in $O(d_{o})$ time by exploiting Jacobian block sparsity, so all $M_{c}$ vectors require $O(M_{c}d_{o})$. The total direct-graph preprocessing cost is therefore

(39) $$C_{\mathrm{pre}}=O\left(N^{2}+Nd_{o}d_{g}+M_{c}d_{o}\right).$$

When the candidate graph is already available, the $O(N^{2})$ term is omitted.

#### 3.3.2. Complexity of Exhaustive Search

For fixed cardinality |S|=K, exhaustive search evaluates Nexh=NK subsets; for the general |S|≤K case, up to Nexh(≤K)=∑k=0KNk subsets may require consideration before resource pruning. For one subset, induced-link and resource evaluation costs at most $O(M_{c})$, accumulation of rank-one projected information contributions costs $O(M\left(S\right)d_{o}^{2})$, and dense numerical-rank plus inverse-trace evaluation costs $O(d_{o}^{3})$. A fixed-cardinality upper-order expression is therefore

(40) Cexh=ONKMc+Mcdo2+do3.

Resource constraints reduce the feasible set in practice but do not remove worst-case combinatorial growth, so exhaustive search is used only for repeated small-scale optimality-gap verification.

#### 3.3.3. Resource-Feasibility Screening Cost

Let $\deg_{c}\left(i\right)$ denote the candidate-graph degree of node *i*. For an unselected candidate, newly activated links and the increments $\Delta M_{i}$, $\Delta T_{\mathrm{slot},i}$, and $\Delta C_{\mathrm{res},i}$ are obtained by scanning its incident candidate links, giving $O(1+\deg_{c}\left(i\right))$ screening cost. At iteration *t*,

$$\begin{array}{cc} C_{\mathrm{screen},t} & =O\left[\left(N-t\right)+\sum_{i\in C\setminus S_{t}}\deg_{c}\left(i\right)\right]\\ & =O(N+M_{c}), \end{array}$$

because the degree sum is at most $2M_{c}$. Over the full procedure,

$$C_{\mathrm{screen}}=O\left[K_{\mathrm{out}}\left(N+M_{c}\right)\right].$$

This dependence exposes the role of graph density: $M_{c}=O\left(N\right)$ for a sparse graph but can reach $O(N^{2})$ in a dense graph.

#### 3.3.4. Complexity of Observable-Seed Construction

Before the projected matrix reaches full gauge-free rank, the inverse-trace objective is unavailable. Stage I therefore compares the numerical rank increase and minimum retained positive eigenvalue produced by each resource-feasible candidate. For candidate *i* at seed iteration *t*,

$$J_{o}\left(S_{t}\cup \left\{i\right\}\right)=J_{o}\left(S_{t}\right)+U_{i}\left(S_{t}\right)U_{i}{\left(S_{t}\right)}^{T}.$$

Explicit formation costs $O(d_{o}^{2}r_{i,t})$, while a dense symmetric eigendecomposition for the rank and minimum positive eigenvalue costs $O(d_{o}^{3})$ per candidate. Hence

$$C_{\mathrm{seed}}=O\left[\sum_{t=0}^{k_{\mathrm{seed}}-1}\sum_{i\in F_{t}}\left(d_{o}^{2}r_{i,t}+d_{o}^{3}\right)\right],$$

and using $n_{t}\leq N-t$ and $r_{i,t}\leq r_{\max}$ gives

(41) $$C_{\mathrm{seed}}=O\left[k_{\mathrm{seed}}N\left(d_{o}^{2}r_{\max}+d_{o}^{3}\right)\right].$$

This seed cost is part of the complete method; the recursive A-optimal complexity below applies only after a full-rank seed exists. A rank-revealing incremental factorization could reduce the seed-stage cost but is not assumed in the reported bound.

#### 3.3.5. Post-Seed Greedy Evaluation with Direct Inversion

After seed construction, a direct-inversion implementation evaluates each feasible candidate by forming and inverting its complete projected information matrix. For candidate *i* at iteration *t*, forming the low-rank increment costs $O(d_{o}^{2}r_{i,t})$ and directly inverting the resulting $d_{o}\times d_{o}$ matrix costs $O(d_{o}^{3})$. Consequently,

$$C_{\mathrm{direct}}=O\left[\sum_{t=k_{\mathrm{seed}}}^{K_{\mathrm{out}}-1}\sum_{i\in F_{t}}\left(d_{o}^{2}r_{i,t}+d_{o}^{3}\right)\right],$$

with upper bound

(42) $$C_{\mathrm{direct}}=O\left[\left(K_{\mathrm{out}}-k_{\mathrm{seed}}\right)N\left(d_{o}^{2}r_{\max}+d_{o}^{3}\right)\right].$$

#### 3.3.6. Post-Seed Greedy Evaluation with Woodbury Updates

For candidate *i* at iteration *t*, $U_{i}\left(S_{t}\right)\in R^{d_{o}\times r_{i,t}}$ contains the projected vectors of newly activated links and $P_{t}=J_{o}{\left(S_{t}\right)}^{-1}$. One Woodbury candidate evaluation requires $O(d_{o}^{2}r_{i,t})$ for $P_{t}U_{i}$, $O(d_{o}r_{i,t}^{2})$ for the inner product, $O(r_{i,t}^{3})$ for inversion of the inner matrix, and the same leading-order multiplication terms for the trace correction and inverse update. Therefore,

(43) $$C_{i,t}^{\mathrm{WB}}=O\left(d_{o}^{2}r_{i,t}+d_{o}r_{i,t}^{2}+r_{i,t}^{3}\right).$$

The exact post-seed recursive cost is

$$C_{A}=O\left[\sum_{t=k_{\mathrm{seed}}}^{K_{\mathrm{out}}-1}\sum_{i\in F_{t}}\left(d_{o}^{2}r_{i,t}+d_{o}r_{i,t}^{2}+r_{i,t}^{3}\right)\right],$$

and the bounds $n_{t}\leq N-t$ and $r_{i,t}\leq r_{\max}$ yield

(44) $$\begin{array}{cc} C_{A}= & O[\left(K_{\mathrm{out}}-k_{\mathrm{seed}}\right)N\\ & \;\times \left(d_{o}^{2}r_{\max}+d_{o}r_{\max}^{2}+r_{\max}^{3}\right)]. \end{array}$$

For a sparse bounded-degree candidate graph, $r_{\max}$ is effectively constant and $M_{c}=O\left(N\right)$, reducing the post-seed stage approximately to $O[\left(K_{\mathrm{out}}-k_{\mathrm{seed}}\right)Nd_{o}^{2}]$. This simplification does not apply automatically in a dense network, where $r_{\max}$ can grow with *N*.

#### 3.3.7. Total Time Complexity of the Proposed Procedure

Combining preprocessing, resource screening, seed construction, and post-seed recursive evaluation gives $C_{\mathrm{total}}=C_{\mathrm{pre}}+C_{\mathrm{screen}}+C_{\mathrm{seed}}+C_{A}$, or explicitly

(45) $$\begin{array}{cc} C_{\mathrm{total}}= & O[N^{2}+Nd_{o}d_{g}+M_{c}d_{o}\\ & \;+K_{\mathrm{out}}\left(N+M_{c}\right)\\ & \;+\sum_{t=0}^{k_{\mathrm{seed}}-1}\sum_{i\in F_{t}}\left(d_{o}^{2}r_{i,t}+d_{o}^{3}\right)\\ & \;+\sum_{t=k_{\mathrm{seed}}}^{K_{\mathrm{out}}-1}\sum_{i\in F_{t}}\left(d_{o}^{2}r_{i,t}+d_{o}r_{i,t}^{2}+r_{i,t}^{3}\right)]. \end{array}$$

Equation (45) shows that the method avoids combinatorial subset enumeration but still depends on candidate-graph density, resource screening, observable-seed size, and iteration-dependent update widths. The compact $O(KNd_{o}^{2})$ expression is therefore only an approximate post-seed description under a sparse bounded-degree graph, not the unconditional complexity of the complete algorithm.

#### 3.3.8. Space Complexity

For serial evaluation, adjacency lists and active-link masks require $O(N+M_{c})$ storage, the explicit gauge-free basis requires $O(Nd_{o})$, projected per-link vectors require $O(M_{c}d_{o})$, and the current projected information matrix plus inverse require $O(d_{o}^{2})$. The largest temporary Woodbury matrices require $O(d_{o}r_{\max}+r_{\max}^{2})$. Hence the total serial space complexity is

(46) $$\begin{array}{c} C_{\mathrm{space}}=O(N+M_{c}+Nd_{o}+M_{c}d_{o}\\ +d_{o}^{2}+d_{o}r_{\max}+r_{\max}^{2}). \end{array}$$

A dense $B_{e}\in R^{d_{o}\times d_{o}}$ is not stored for every candidate link because that would require $O(M_{c}d_{o}^{2})$ memory; only $a_{e}$ is retained and its rank-one contribution is formed when needed. Likewise, no fixed candidate-node increment is stored because $\Delta E_{i}\left(S_{t}\right)$ and $U_{i}\left(S_{t}\right)$ depend on the current selected node subset. Parallel evaluation within an iteration would require additional memory proportional to the number of concurrent candidates; the bound in Equation (46) assumes sequential candidate evaluation.

As summarized in Table 2, exhaustive search retains combinatorial dependence on the candidate-node number. The proposed heuristic replaces subset enumeration with sequential candidate evaluation and uses low-rank recursive updates after a full-rank observable seed has been constructed.

The resulting procedure is polynomial in the number of candidate evaluations, but its practical runtime is not determined by *N* alone. Candidate-graph density, the observable-seed stage, resource screening, and the distribution of $r_{i,t}$ also affect the computational cost. Consequently, no unconditional real-time claim is made solely from the asymptotic expression. Empirical runtime and memory measurements are reported in the scalability experiment.

Candidate evaluations within the same iteration are conditionally independent given the current selected node subset and may be parallelized. However, the experiments in this paper use a serial implementation so that all reported running times correspond to the complexity model derived above.

## 4. Simulation Validation

### 4.1. Experimental Settings and Evaluation Metrics

To evaluate the proposed G-GDOP-based UWB cooperative sensor node pre-selection method, simulation experiments are conducted in a controllable three-dimensional environment. The experiments evaluate the applicability boundary of G-GDOP, information–accuracy–resource trade-offs under selected-node and ranging-slot budgets, repeated small-scale comparisons with exhaustive enumeration, robustness to Gaussian ranging noise, heterogeneous ranging noise, and NLOS bias, sensitivity to predicted positions and link-quality estimates, and computational scalability.

#### 4.1.1. Simulation Scenario and General Parameter Settings

A three-dimensional UWB cooperative localization simulation environment is constructed in this study. The simulation region is set as 1000m×1000m×300m. Simulated UAV-borne sensor nodes are uniformly and randomly distributed in the three-dimensional space. When the distance between two nodes is smaller than the communication/ranging radius $R_{c}$, an effective UWB ranging link is assumed to be established between them. Unless otherwise specified, the default total number of nodes is set to N=30, and the default number of selected nodes is set to K=10. Unless otherwise specified, the experiments use the anchor-free range-only formulation: all *N* mobile nodes remain in the localization state, *K* nodes are scheduled as active UWB ranging initiators, and the corresponding active links are induced by that selection. Each Monte Carlo trial represents one static network snapshot; closed-loop UAV motion and communication dynamics are outside the present simulation scope. The G-GDOP, A-optimal, and D-optimal criteria use the gauge-free projected Fisher information matrix and full-rank observability test defined in Section 2.3. For localization-error evaluation, the estimated configuration of all *N* mobile nodes is rigidly aligned with the corresponding ground-truth configuration before the RMSE is calculated. Hence, the reported RMSE measures the recovery accuracy of the complete mobile-node configuration under the ranging topology induced by the selected nodes, rather than the error of only the selected nodes. Table 3 lists the general simulation parameters used in this study.

The UWB ranging-noise parameters are chosen to represent decimeter-level UWB ranging accuracy and the degradation of ranging precision under LOS/NLOS conditions. The simulation region, node scale, and communication/ranging radius are used to construct a controllable three-dimensional cooperative ranging network. Their effects will be further analyzed in the subsequent node-budget experiment, noise robustness experiment, and scalability experiment.

#### 4.1.2. UWB Ranging Error and Estimated Link-Quality Settings

For each measurable UWB link e=(i,j), the simulated observation is generated according to

(47) $$z_{e}={∥p_{i}-p_{j}∥}_{2}+b_{e}+n_{e},\;n_{e}∼N\left(0,\sigma_{e}^{2}\right).$$

Three ranging-error regimes are considered. First, in the homoscedastic Gaussian-noise experiments, all active links use

$$\sigma_{e}=0.10\;m,\;b_{e}=0.$$

Second, in the heteroscedastic random-noise experiment, the links are divided into LOS, wLOS, and NLOS/degraded classes with proportions of 50%, 30%, and 20%, respectively. Their random-error standard deviations are set to 0.05m, 0.10m, and 0.30m, while all systematic bias terms are set to zero. Third, in the NLOS-bias experiment, the same link classes and random standard deviations are retained, and a positive bias is introduced only into the NLOS/degraded links:

$$b_{e}=\left\{\begin{array}{cc} 0,\; & c_{e}\in \left\{\mathrm{LOS},\mathrm{wLOS}\right\},\\ b_{\mathrm{NLOS}},\; & c_{e}=\mathrm{NLOS}, \end{array}\right.$$

where

$$b_{\mathrm{NLOS}}\in \left\{0.30,\;0.50,\;0.70,\;1.00\right\}\;m.$$

Two link-quality information settings are considered. In the oracle setting, the true class and true random-error variance are provided to the pre-selection method. This setting is used only as an ideal upper-reference benchmark for evaluating the weighting mechanism.

In the practical estimated-quality setting, the link class is imperfectly identified. Let η∈[0,1] denote the adjacent-class misclassification probability. The class-confusion matrix is defined as

(48) $$\Gamma \left(\eta \right)=\left[\begin{array}{ccc} 1-\eta & \eta & 0\\ \eta /2 & 1-\eta & \eta /2\\ 0 & \eta & 1-\eta \end{array}\right],$$

where the rows correspond to the true classes {LOS,wLOS,NLOS} and the columns correspond to the estimated classes.

In addition to class misclassification, a relative standard-deviation estimation error is introduced as

(49) $${\hat{\sigma }}_{e}=\max\left\{\sigma_{{\hat{c}}_{e}}\left(1+\epsilon_{e}\right),\sigma_{\min}\right\},\;\epsilon_{e}∼U\left(-\rho ,\rho \right),$$

where ρ controls the variance-estimation uncertainty and $\sigma_{\min}>0$ prevents a nonphysical nonpositive standard deviation. The confusion matrix and variance perturbation form a controlled sensitivity model rather than a trained LOS/NLOS classifier or a complete non-Gaussian UWB channel model. Thus, the experiment tests the effect of imperfect quality information within the prescribed model, while validation with classifier outputs and measured UWB error distributions remains outside the scope of the present study. The estimated weight supplied to the practical pre-selection method is

$${\hat{w}}_{e}=\frac{\sigma_{0}^{2}}{{\hat{\sigma }}_{e}^{2}}.$$

All competing methods in a paired Monte Carlo trial use the same node topology, true link classes, random ranging noise realization, NLOS bias realization, and estimated-quality realization. The physical UWB measurements are generated using the true random-error covariance $R_{S}$. The practical weighted pre-selection method and the practical weighted nonlinear localization solver use ${\hat{R}}_{S}$. The true covariance is supplied to a method only when that method is explicitly labeled as an oracle benchmark. In the unmodeled-NLOS-bias stress test, the injected positive bias magnitude is not included in the selection weights and is not supplied to the nonlinear localization solver. Thus, ${\hat{b}}_{S}=0$ and the experiment evaluates sensitivity to a residual systematic error rather than an oracle bias-aware estimator. Therefore, performance differences within each trial are caused by the pre-selection strategy rather than by independently generated random conditions.

#### 4.1.3. Controlled Predicted-Position Uncertainty Experiment

A controlled predicted-position uncertainty experiment is introduced to quantify how errors in the geometry available to the online pre-selection algorithm affect the information objective, selected node subset, observability assessment, and downstream nonlinear localization. For each of the $R_{\mathrm{MC}}=100$ paired Monte Carlo trials, the true node positions $p_{i}$ are generated once. A trial-specific standardized position-error realization $z_{i}∼N\left(0,I_{3}\right)$ is also generated once and is shared across all tested uncertainty levels. The predicted position used by the online selector is constructed as

(50) $${\hat{p}}_{i}\left(\sigma_{p}\right)=p_{i}+\sigma_{p}z_{i},$$

where $\sigma_{p}$ denotes the per-coordinate standard deviation of the prediction error. The tested levels are

$$\sigma_{p}\in \left\{0,\;0.5,\;1,\;2,\;5,\;10,\;20,\;40\right\}\;m.$$

The default prediction-error setting used in the preceding experiments corresponds to $\sigma_{p}=2\;m$ per coordinate.

To isolate predicted-geometry uncertainty from topology-discovery and link-quality-estimation errors, the true node configuration, candidate ranging graph, link-quality classes, ranging noise realization, resource parameters, localization initialization, and standardized position-error directions are held fixed across all $\sigma_{p}$ levels within the same trial. The candidate graph is constructed once from the true node configuration using $R_{c}=550\;m$ and is not regenerated from the perturbed predicted positions. The experiment uses N=30 nodes, a selected-node budget of K=10, the heterogeneous link classes in Table 4, zero link-quality estimation error (η=ρ=0), and no injected systematic NLOS bias. Thus, the controlled variable is the geometry supplied to the pre-selection stage.

At each uncertainty level, the practical Weighted G-GDOP method constructs the gauge-free basis, ranging Jacobian, observability test, and A-optimal objective using ${\hat{p}}_{i}\left(\sigma_{p}\right)$. A true-geometry reference is obtained by running the same Weighted G-GDOP greedy procedure with ${\hat{p}}_{i}=p_{i}$, while keeping the candidate graph, link weights, resource constraints, and all other trial-level quantities unchanged. This reference is therefore a geometry-knowledge reference for the same greedy method rather than a globally optimal subset.

For the selected node subset obtained using predicted geometry, $S_{\mathrm{pred}}$, the absolute relative mismatch between the online objective and the objective evaluated for the same subset under true geometry is

(51) $$\delta_{\Phi }=\frac{\left|{\hat{\Phi }}_{A}^{\mathrm{pred}}\left(S_{\mathrm{pred}}\right)-{\hat{\Phi }}_{A}^{\mathrm{true-geom}}\left(S_{\mathrm{pred}}\right)\right|}{{\hat{\Phi }}_{A}^{\mathrm{true-geom}}\left(S_{\mathrm{pred}}\right)}.$$

Both quantities in Equation (51) use the same estimated link weights; only the geometry used to construct the projected information matrix is changed. Subset agreement with the true-geometry reference selected node subset $S_{\mathrm{true}}$ is quantified using

(52) $$J_{\mathrm{sub}}=\frac{\left|S_{\mathrm{pred}}\cap S_{\mathrm{true}}\right|}{\left|S_{\mathrm{pred}}\cup S_{\mathrm{true}}\right|}.$$

The information-quality loss caused by geometry-dependent subset selection is additionally measured under true geometry as

(53) $$\Delta_{\Phi }^{\mathrm{sel}}=\frac{{\hat{\Phi }}_{A}^{\mathrm{true-geom}}\left(S_{\mathrm{pred}}\right)-{\hat{\Phi }}_{A}^{\mathrm{true-geom}}\left(S_{\mathrm{true}}\right)}{{\hat{\Phi }}_{A}^{\mathrm{true-geom}}\left(S_{\mathrm{true}}\right)}.$$

The same selected node subset obtained using predicted geometry is also checked for observability under both predicted and true geometry using the common projected-FIM rank threshold $\tau_{J}$. Nonlinear localization is then performed with the common downstream solver, and the paired RMSE difference is defined as

(54) $$\Delta \mathrm{RMSE}={\mathrm{RMSE}}_{\mathrm{pred}}-{\mathrm{RMSE}}_{\mathrm{true-geom}}.$$

Observability-status changes, additional selection or localization failures relative to the true-geometry-reference-success trials, and the contributing sample counts are retained explicitly. Continuous metrics are summarized by the median and pointwise 95% percentile bootstrap confidence interval, whereas binary rates use the common 95% Wilson interval defined in the paired Monte Carlo protocol.

#### 4.1.4. Comparison Methods and Ablation Versions

To comprehensively evaluate the performance of the proposed method, the comparison set includes Random Selection, Nearest Neighbor, Max–Min Distance, D-Optimal, Feasible Traditional GDOP, Unweighted G-GDOP, Weighted G-GDOP, and the All-Node Reference. Meanwhile, to verify the effects of topology-coupled modeling and heteroscedastic link-noise weighting in the proposed model, two ablation versions, namely Unweighted G-GDOP and Weighted G-GDOP, are introduced for comparison. Table 5 summarizes the comparison methods and G-GDOP variants used in the subsequent experiments.

For a fair comparison, all-node pre-selection methods except the All-Node Reference are evaluated under the same node budget *K*. In the heteroscedastic link-noise ablation experiment, the comparison between Feasible Traditional GDOP and Unweighted G-GDOP is used to analyze the effect of topology-induced multi-node coupling. The comparison between Unweighted G-GDOP and Weighted G-GDOP is used to analyze the contribution of heteroscedastic link-noise weighting.

In the resource-constrained experiments, all competing sequential selection methods use the same candidate graph, selected-node budget, induced-link budget, ranging-slot budget, residual-energy threshold, and general resource-cost budget. A candidate that would violate any common budget is removed from the feasible candidate set before the method-specific selection score is evaluated.

For exhaustive search, only subsets satisfying all common resource and local observability constraints are enumerated. The All-Node Reference is reported as a resource-unconstrained reference and is not included as a feasible competitor when its resource consumption exceeds the prescribed budget.

#### 4.1.5. Nonlinear Localization Backend Used for RMSE Evaluation

The nonlinear localization backend is used only to evaluate the ranging topology returned by a pre-selection method; it does not provide ground-truth geometry, an objective value, or an initialization to the selector. Within each paired trial, every competing method uses the same localization backend and the same trial-level initialization protocol.

A classical multidimensional-scaling (MDS) configuration is first constructed from the finite symmetric pairwise-range matrix supplied to the localization subproblem. Let D denote this range matrix and $H_{c}=I_{N}-1_{N}1_{N}^{T}/N$. The double-centered Gram matrix is

$$B=-\frac{1}{2}H_{c}\left(D⊙D\right)H_{c}.$$

After numerical symmetrization, the three largest nonnegative eigenvalues and their eigenvectors form the three-dimensional MDS initialization; a rank-deficient result is padded with zero coordinates. If this initialization is invalid, a centered random initialization with scale determined by the median positive range is used. Ground-truth coordinates are not used to initialize MDS or the iterative estimator. The diagnostic backend does not perform method-specific oracle completion of missing measurements: a localization subproblem that cannot provide the required finite input is retained as a localization failure.

The subsequent weighted Gauss–Newton stage minimizes

$$\min_{x}\;\sum_{e\in E(S)}\frac{{\left[z_{e}-{\hat{b}}_{e}-h_{e}\left(x\right)\right]}^{2}}{{\hat{\sigma }}_{e}^{2}},$$

where ${\hat{b}}_{e}=0$ in the unmodeled-bias stress test. At iteration *q*, the damped normal equations are

$$\left(H_{q}^{T}\hat{W}H_{q}+\lambda I\right)\delta_{q}=H_{q}^{T}\hat{W}r_{q},\;\lambda =10^{-4}.$$

The maximum iteration number is 50. Iteration terminates when $∥\delta_{q}{∥}_{2}<10^{-5}$ or when the change in residual norm is below $10^{-6}$. A normal system with reciprocal condition number below $10^{-12}$, an empty usable Jacobian, or a nonfinite update is treated as a solver failure rather than being repaired using true coordinates.

Because range-only localization is identifiable only up to a rigid transformation, the final estimate is aligned to the ground-truth configuration solely for RMSE evaluation by an SVD-based orthogonal Procrustes transform with translation and rotation but no scaling; reflections are rejected. A trial is classified as localization-success only when the backend returns a finite N×3 estimate and the rigid-aligned RMSE is finite. Failed trials are retained in status accounting and are not regenerated. This common backend is a diagnostic evaluation tool, not a claim that MDS–Gauss–Newton is the only or preferred downstream estimator; factor-graph and incremental-smoothing validation remains future work.

#### 4.1.6. Evaluation Metrics and Statistical Settings

To evaluate the performance of different cooperative sensor node pre-selection methods from multiple perspectives, this study adopts localization accuracy, the A-optimal metric, the G-GDOP metric, running time, and the proportion of NLOS/degraded links as evaluation metrics.

*Localization accuracy.*

Let ${\hat{p}}_{i}^{\;\mathrm{ali}}$ denote the estimated position of mobile node *i* after rigid alignment with the ground-truth configuration, and let $p_{i}$ denote its true position. The full-state localization RMSE is defined as

(55) $$\mathrm{RMSE}=\sqrt{\frac{1}{N}\sum_{i=1}^{N}{∥{\hat{p}}_{i}^{\;\mathrm{ali}}-p_{i}∥}_{2}^{2}}.$$

The RMSE is calculated over all *N* mobile sensor nodes, including both the selected nodes and the unselected ranging responders. Therefore, changing the selected-node budget *K* changes the measurement topology but does not change the set of states included in the RMSE calculation.

*Online A-optimal selection metric.*

For a feasible topology induced by the selected node subset, the practical online A-optimal selection metric is

(56) $${\hat{\Phi }}_{A}\left(S\right)=\frac{1}{d_{o}}\mathrm{tr}\left[{\hat{\bar{J}}}_{o}{\left(S\right)}^{-1}\right].$$

This metric is constructed from predicted coordinates and estimated link-error variances and is used by the practical pre-selection algorithm.

*Oracle true-covariance A-optimal metric.*

For diagnostic evaluation, the selected node subset is also assessed under the true random-error covariance:

(57) $$\Phi_{A,\mathrm{true}}\left(S\right)=\frac{1}{d_{o}}\mathrm{tr}\left[{\bar{J}}_{o}^{⋆}{\left(S\right)}^{-1}\right].$$

This metric is unavailable to the practical online algorithm and is reported only to quantify the effect of covariance-estimation errors.

##### First-Order Local RMSE Surrogate Under Model Mismatch

The first-order local RMSE surrogate is calculated using Equation (15). It incorporates the true physical random-error covariance, the covariance used by the practical estimator, and the residual bias vector. This metric is used only as a secondary diagnostic and is not substituted for the actual nonlinear localization RMSE.

*Generalized GDOP metric.*

For a feasible topology induced by the selected node subset, the generalized GDOP metric is defined as

(58) $$\mathrm{G-GDOP}\left(S\right)=\sqrt{\mathrm{tr}\left[J_{o}{\left(S\right)}^{-1}\right]}.$$

This metric describes the local error amplification level in the gauge-free coordinates under the joint effects of link geometry, network topology, and estimated link-quality weighting.

*Induced active-link number.*

The number of induced active ranging links is evaluated as

M(S)=|E(S)|.

This metric measures the number of UWB ranging operations activated by the selected node subset.

*Ranging-slot occupation.*

The total ranging-slot occupation is evaluated as

$$T_{\mathrm{slot}}\left(S\right)=\sum_{e\in E(S)}s_{e}.$$

In the default unit-slot experiment, $s_{e}=1$, so that $T_{\mathrm{slot}}\left(S\right)=M\left(S\right)$. A nonuniform-slot sensitivity experiment may assign different $s_{e}$ values to different ranging protocols or link-quality classes.

*General resource cost.*

The normalized general resource cost is evaluated using Equation (26). Because this quantity is an engineering cost proxy rather than a hardware-calibrated energy measurement, the raw selected-node number, induced-link number, and ranging-slot occupation are reported together with it.

##### A-Optimal Quality Retention and Resource Saving

The dimensionless A-optimal quality-retention ratio $Q_{A}\left(S\right)$ and the resource-usage ratios $\rho_{\mathrm{link}}$, $\rho_{\mathrm{slot}}$, and $\rho_{\mathrm{res}}$ are used to characterize the information–resource trade-off relative to the All-Node Reference.

##### Selection-Stage Running Time

The reported running time includes only the cooperative sensor node pre-selection procedure. UWB measurement generation, nonlinear localization, rigid alignment, result serialization, and figure generation are excluded.

For the proposed method, the following runtime components are recorded separately:

1. candidate-graph and per-link-vector preprocessing time;
2. resource-feasibility screening time;
3. observable-seed construction time;
4. post-seed A-optimal candidate-evaluation time; and
5. total pre-selection time.

Each timing instance is executed once for numerical-library warm-up and is then repeated ten times using exactly the same input data. The median of the ten repeated measurements is retained as the trial-level running time. Across paired Monte Carlo trials, the reported point estimate is the median of the trial-level running times, and the uncertainty band is the corresponding pointwise 95% percentile bootstrap confidence interval.

##### Graph and Update-Structure Indicators

To interpret the observed runtime, the following structural quantities are recorded for every trial:

$$M_{c}=\left|E_{c}\right|,$$

$$\rho_{c}=\frac{2M_{c}}{N(N-1)},$$

$$N_{\mathrm{eval}}=\sum_{t=0}^{K_{\mathrm{out}}-1}\left|F_{t}\right|,$$

$$\bar{r}=\frac{\sum_{t}\sum_{i\in F_{t}}r_{i,t}}{N_{\mathrm{eval}}},$$

and

$$r_{\max}=\max_{t,i}r_{i,t}.$$

The observable-seed size $k_{\mathrm{seed}}$ and the returned selected-node count $K_{\mathrm{out}}$ are also recorded. These quantities allow the empirical running time to be interpreted together with the theoretical complexity terms.

##### Peak Memory Consumption

Peak memory consumption is recorded during the pre-selection stage. The measurement excludes the memory used by nonlinear localization, plotting, and result files. The reported value is used to evaluate the practical influence of explicitly storing $Q_{o}$, the per-link projected vectors, and the current inverse information matrix.

##### Proportion of NLOS/Degraded Links

The proportion of NLOS/degraded links is defined as

(59) $$\rho_{\mathrm{NLOS}}=\frac{N_{\mathrm{NLOS}}^{\mathrm{sel}}}{N_{\mathrm{link}}^{\mathrm{sel}}},$$

where $N_{\mathrm{NLOS}}^{\mathrm{sel}}$ denotes the number of NLOS/degraded links in the subnetwork induced by the selected node subset, and $N_{\mathrm{link}}^{\mathrm{sel}}$ denotes the total number of effective ranging links in the subnetwork induced by the selected node subset. This metric is used to evaluate whether a method can reduce the inclusion of low-quality ranging links in the cooperative localization subnetwork.

#### 4.1.7. Paired Monte Carlo Protocol, Failure Handling, and Statistical Reporting

Unless otherwise stated, each operating condition is evaluated using $R_{\mathrm{MC}}=100$ paired Monte Carlo trials. For the *r*-th trial, the true node configuration, predicted node configuration, candidate ranging graph, true link-quality classes, estimated link-quality realization, standardized ranging noise realization, NLOS-bias realization, resource parameters, and nonlinear localization initialization are generated only once and are shared by all competing methods.

The true node coordinates are used only to generate the physical UWB measurements and to calculate the rigid-aligned full-state localization RMSE. All practical pre-selection methods construct their ranging Jacobians and online information matrices using the same predicted node coordinates. Similarly, the true link-error covariance is used to generate the physical ranging errors, whereas a practical weighted method uses the same estimated link-error covariance available in that trial. The true covariance is supplied only to methods explicitly labeled as oracle benchmarks.

Common random numbers are also used across parameter levels whenever the corresponding physical interpretation permits. In the Gaussian noise sweep, a standardized realization $\xi_{e}^{\left(r\right)}$ is generated once and scaled by the specified noise standard deviation. In the NLOS-bias sweep, the node topology, link classes, standardized random errors, estimated link-quality realization, and localization initialization are held fixed while only the injected bias magnitude is changed. In the node-, link-, and ranging-slot-budget sweeps, the candidate topology and measurement realization are kept unchanged within each trial.

No method-specific trial regeneration is permitted. If a method cannot construct a selected node subset satisfying the common resource constraints, the result is recorded as resource-infeasible. If the returned projected information matrix does not satisfy

$$r_{o}\left(S\right)=d_{o},$$

the result is recorded as unobservable. An unobservable matrix is not made artificially invertible through numerical regularization. If the nonlinear localization solver does not converge to a finite solution, or if the rigid-aligned RMSE is non-finite, the result is recorded as a localization failure. Failed trials are not replaced by newly generated topologies, noise realizations, or initial values.

The resource-feasibility rate, observability rate, selection-success rate, and nonlinear localization success rate are calculated using all $R_{\mathrm{MC}}$ common trials as the denominator. These binary rates are reported together with 95% Wilson confidence intervals.

For a continuous metric *y*, the reported point estimate for method *m* is the median over the trials in which that metric is defined:

$${\tilde{y}}_{m}={\mathrm{median}}_{r\in V_{m}}y_{m}^{\left(r\right)}.$$

Here, $V_{m}$ is the set of trials in which the metric is defined. Information matrix metrics are summarized over observable trials, whereas nonlinear localization RMSE is summarized over localization-success trials. The number of contributing trials is reported with every continuous summary.

Uncertainty intervals for continuous metrics are calculated using $B_{\mathrm{boot}}=10,000$ percentile-bootstrap repetitions. Monte Carlo trial indices are resampled with replacement. Within one bootstrap repetition, the same resampled trial indices are applied jointly to all competing methods so that the within-trial pairing is preserved. The 2.5th and 97.5th percentiles of the bootstrap distribution form the pointwise 95% confidence interval.

For a pairwise comparison between method *m* and reference method *b*, only trials in which both methods provide a valid value are used. The paired median difference is defined as

$${\tilde{\Delta }}_{m,b}={\mathrm{median}}_{r\in V_{m}\cap V_{b}}\left(y_{m}^{\left(r\right)}-y_{b}^{\left(r\right)}\right).$$

Its 95% confidence interval is obtained by jointly resampling the paired trial indices. The number of common valid trials $n_{\mathrm{pair}}$ is reported for every pairwise comparison.

Unless otherwise stated, curves and point estimates show medians, and shaded regions or error bars show pointwise 95% bootstrap confidence intervals. Interquartile ranges may be shown only as supplementary distributional summaries and are not used as the primary uncertainty intervals.

#### 4.1.8. Computational Environment

Since the experiments in this study include running-time comparisons, all formal experiments are conducted under a unified computational environment to ensure the interpretability of the computational-efficiency evaluation. The Exp01–Exp09 selection, evaluation, and reporting pipelines use the validated ggdop_core_step9 backend under Python 3.13.12 on a 64-bit Windows 11 computer equipped with an AMD Ryzen 7 4800U processor (Advanced Micro Devices, Inc., Santa Clara, CA, USA) and 16 GB DDR4-3200 memory. The reported running time is the median pre-selection time obtained from ten repeated executions for each network topology after one numerical-library warm-up run. All methods are executed serially under the same process and numerical-thread configuration. Measurement generation, nonlinear localization, rigid alignment, plotting, and file serialization are excluded from the reported pre-selection time.

### 4.2. Simulation Results

#### 4.2.1. Applicability Boundary and Local Diagnostic Consistency

**Figure 5:**
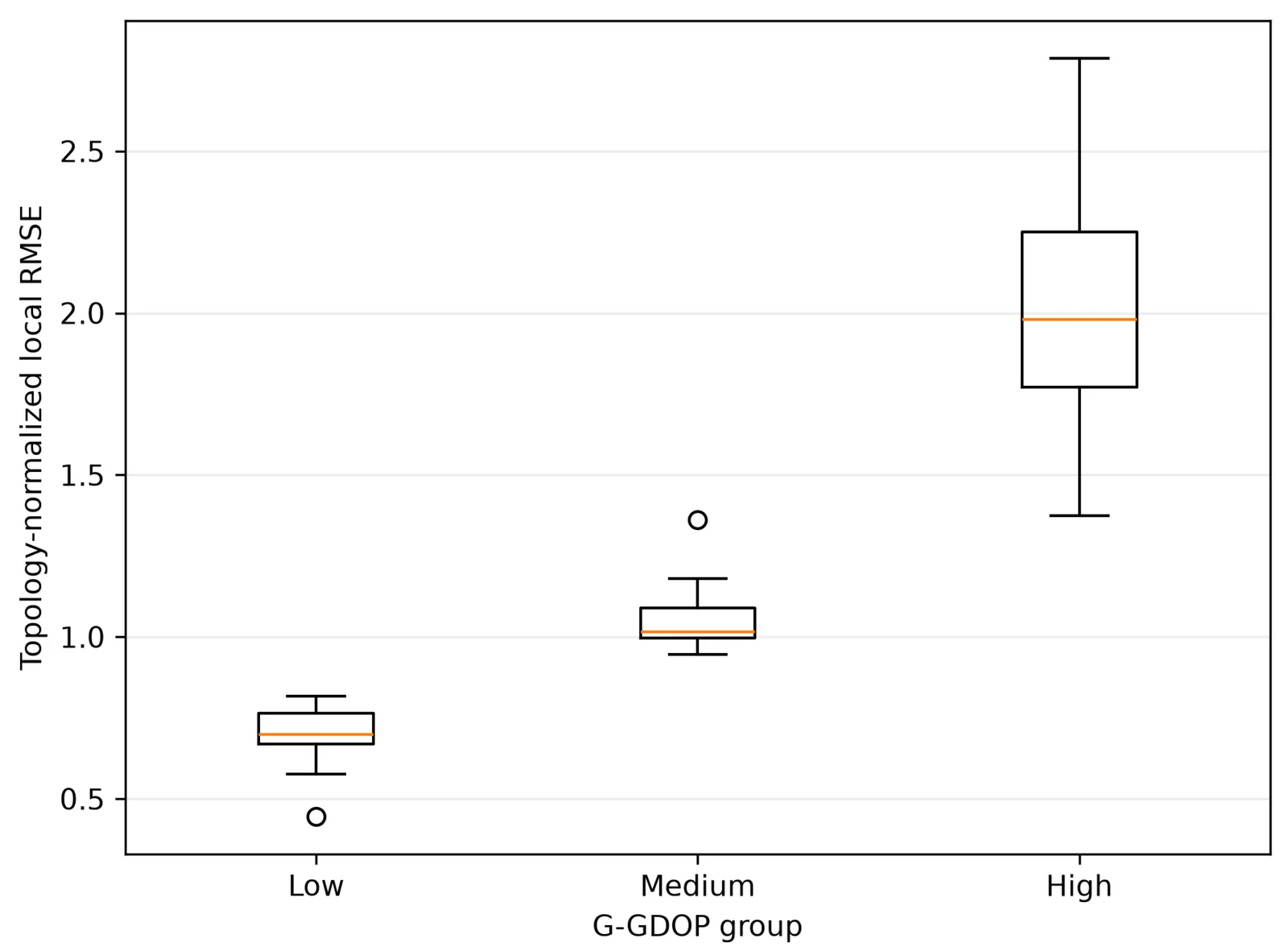
Same-topology ranking validation of G-GDOP. Topology-normalized local RMSE is shown for the Low, Medium, and High G-GDOP groups.

Table 6 summarizes the applicability boundary and the local diagnostic behavior of G-GDOP. In the global diagnostic set, the median G-GDOP was 24.95 [19.97, 28.34], and the topology-level median RMSE was 0.419 [0.326, 0.482] m. The local analysis yielded median values of 0.1316 [0.0896, 0.1737] for the CRLB proxy and 0.2723 [0.1976, 0.3419] for the empirical local MSE. The clearest ordering appeared in the normalized local RMSE, which increased from 0.698 [0.673, 0.752] in the Low group to 1.014 [0.999, 1.068] in the Medium group and 1.980 [1.806, 2.180] in the High group. This ordered separation supports the use of G-GDOP as a ranking indicator within the tested domain, without implying a coefficient-based global association. The same-topology ordering is visualized in Figure 5.

#### 4.2.2. Information–Accuracy–Resource Trade-Offs

**Figure 6:**
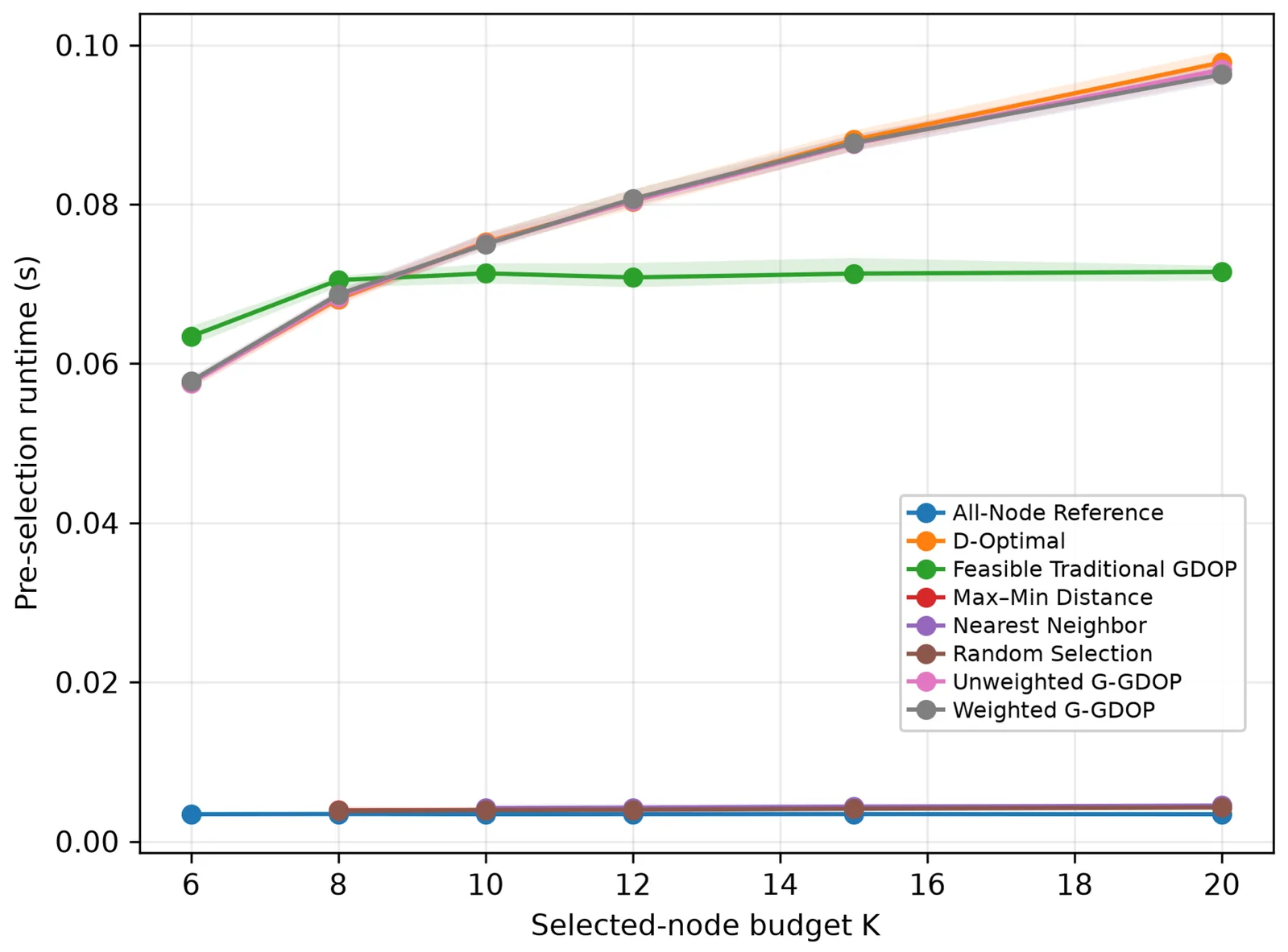
Pre-selection runtime versus the selected-node budget *K* in the node-budget experiment for the tested selection methods and the All-Node Reference.

**Figure 7:**
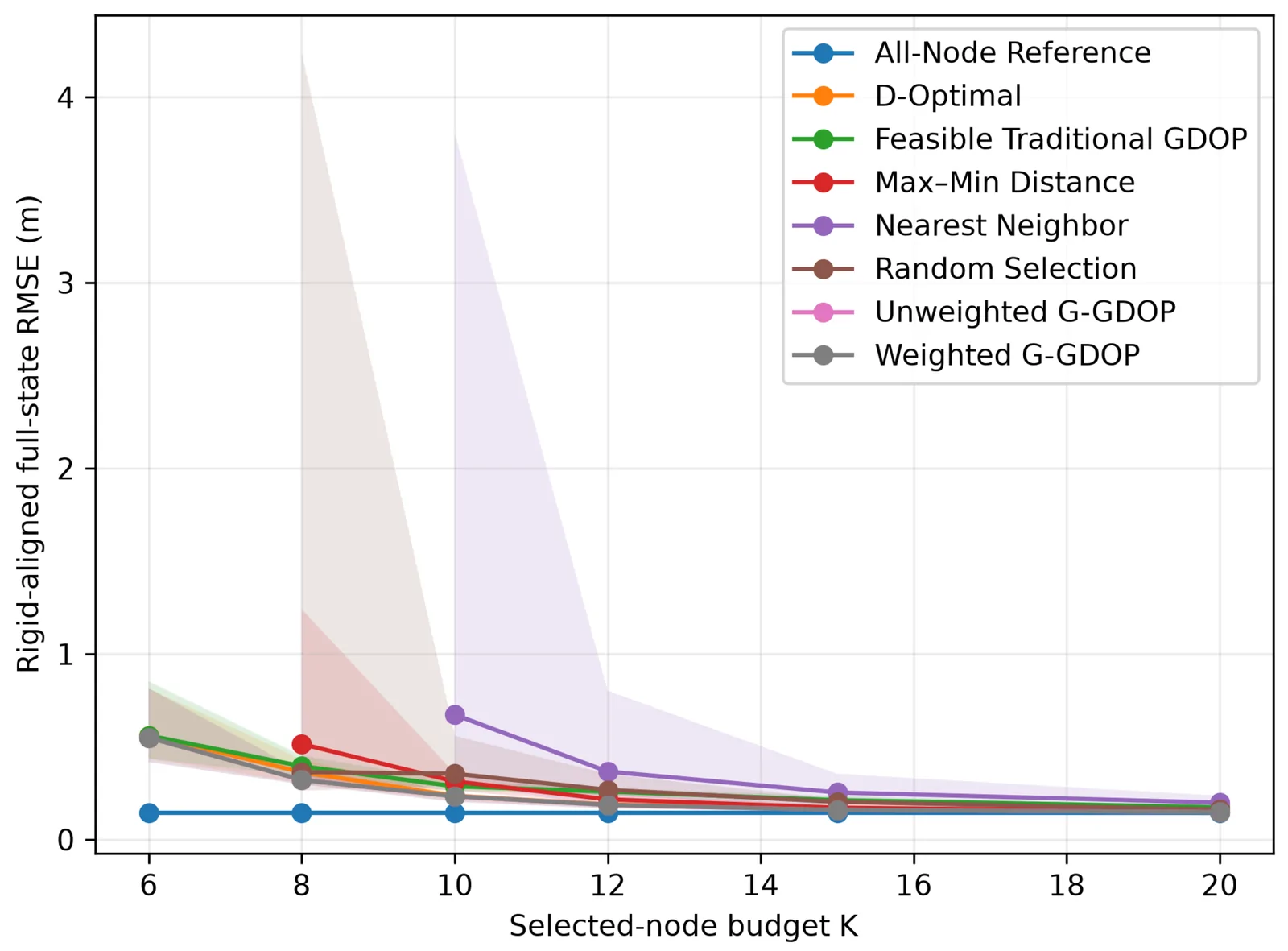
Rigid-aligned full-state RMSE versus the selected-node budget *K* in the node-budget experiment.

The node- and ranging-slot-budget results in Table 7 reveal the expected information–accuracy–resource trade-off. For Weighted G-GDOP, increasing *K* from 6 to 20 raised the median number of active links from 109 to 214 and the median runtime from 0.0578 to 0.0963 s, while reducing the median normalized objective from 7.994 to 0.777 and the median RMSE from 0.550 to 0.148 m. Relaxing the ranging-slot budget from $\beta_{\mathrm{slot}}=0.45$ to 1.0 produced a corresponding change: the active-link count increased from 112 to 214, whereas the objective and RMSE decreased from 6.694 to 0.777 and from 0.435 to 0.148 m, respectively. At K=20, Weighted G-GDOP activated 214 links, compared with 229 for the All-Node Reference, and achieved median objective and RMSE values of 0.777 and 0.148 m; the corresponding all-node values were 0.745 and 0.143 m. Shared non-success cases remain included in the formal status accounting and are not attributed to individual methods. Figure 6 and Figure 7 show the corresponding runtime growth and RMSE reduction as *K* increases.

The monotonic statement above applies to the reported medians of Weighted G-GDOP, not to every comparison method or every trial. In Figure 7, the Nearest Neighbor curve can increase over part of the budget range. Adding selected nodes according to distance alone changes the induced topology but does not guarantee improved geometric diversity or link reliability, and the final nonlinear RMSE also depends on initialization and convergence. Accordingly, the manuscript does not claim that RMSE must decrease monotonically with *K* for all selection rules, nor that Weighted G-GDOP is the lowest-RMSE method under every tested budget.

#### 4.2.3. Small-Scale Comparison with Exhaustive Enumeration

**Figure 8:**
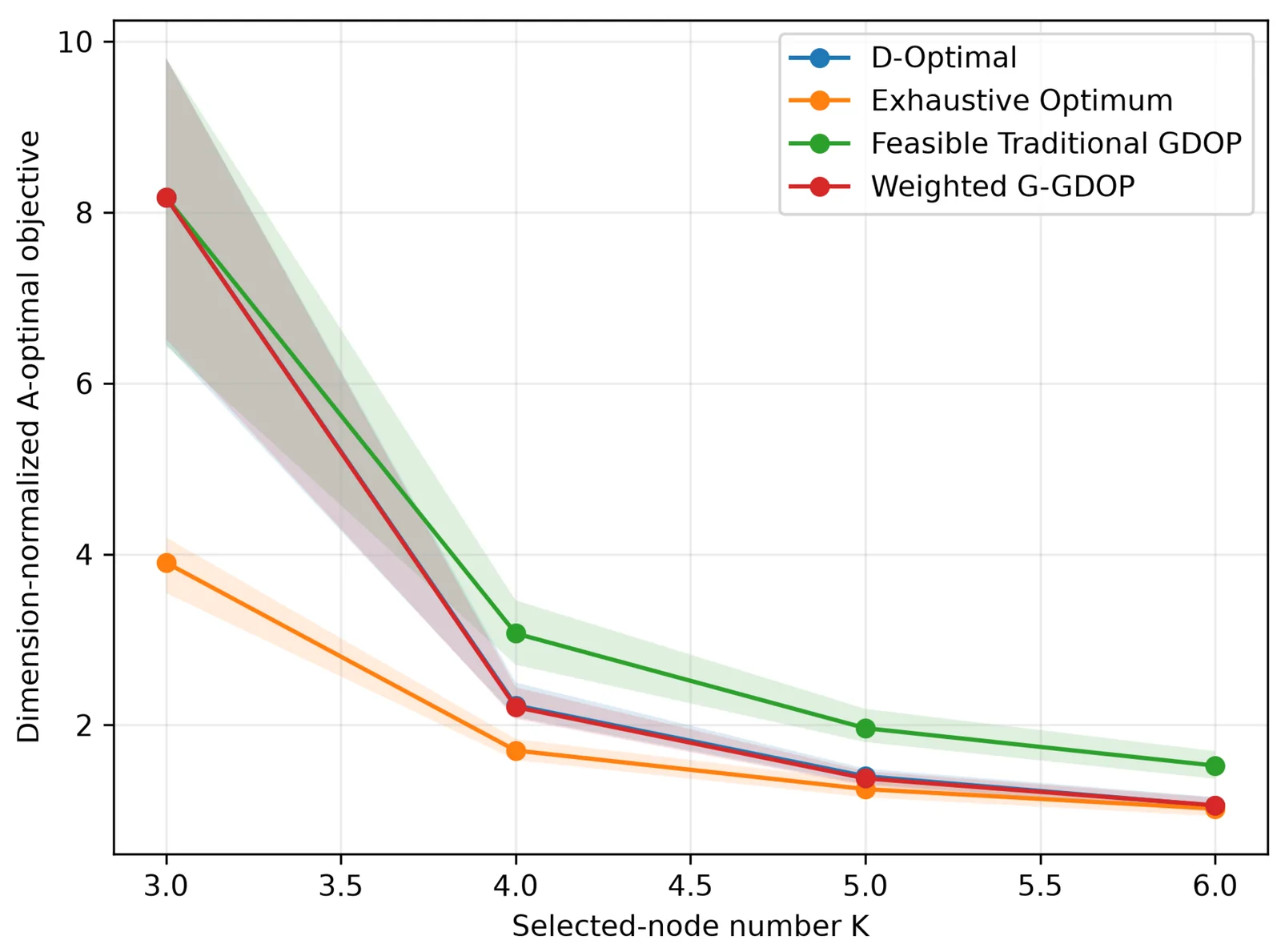
Dimension-normalized A-optimal objective versus the selected node number *K* in the small-scale exhaustive-enumeration benchmark.

Table 8 provides an absolute small-scale comparison between Weighted G-GDOP and the Exhaustive Optimum. For K=3, 4, 5, and 6, the respective median normalized objective pairs (Weighted G-GDOP versus Exhaustive Optimum) were 8.179 versus 3.901, 2.210 versus 1.702, 1.375 versus 1.250, and 1.060 versus 1.021. The absolute objective values therefore became progressively closer over the tested range of *K*. Meanwhile, the runtime of Weighted G-GDOP increased only from 0.0050 to 0.0074 s, whereas exhaustive enumeration increased from 0.0308 to 0.1279 s. These results are used solely as an absolute benchmark and do not establish a general approximation guarantee. Figure 8 visualizes the convergence of the tested objective values over the small-scale range of *K*.

The comparatively large difference at K=3 was examined separately by tracing the same 100 paired Monte Carlo trials used in the formal comparison. In all 100 trials, the observable-seed size was $k_{\mathrm{seed}}=3$ and the final selected-node count was also $k_{\mathrm{out}}=3$; hence, the post-seed A-optimal Stage II performed zero node-addition iterations. The Stage I seed was identical to the exhaustive-optimal selected node subset in only 7 of 100 trials, and the median paired true-objective gap relative to exhaustive enumeration was 79.0%.

The first divergence from the exhaustive-optimal selected node subset was attributed to the retained minimum-eigenvalue criterion in 83 trials and to the deterministic node-ID tie-break in 10 trials; the remaining seven trials did not diverge. Across the 300 Stage I seed decisions, numerical rank increase reduced the feasible candidate set in 12 decisions, retained minimum eigenvalue further reduced the surviving candidate set in all 300 decisions, and node ID was finally decisive in 31. The incremental induced-link number, ranging-slot occupation, and general resource cost did not discriminate among feasible candidates in any of these 300 decisions. In this N=12, K=3 setting, every final selected node subset activated 30 links, occupied 30 ranging slots, and had a general resource cost of 33. As a diagnostic check, when the observable seed was selected directly by the final A-optimal objective instead of the Stage I lexicographic ordering, the exhaustive optimum was recovered in all 100 trials.

#### 4.2.4. Robustness, Ablation, and Sensitivity Analyses

**Figure 9:**
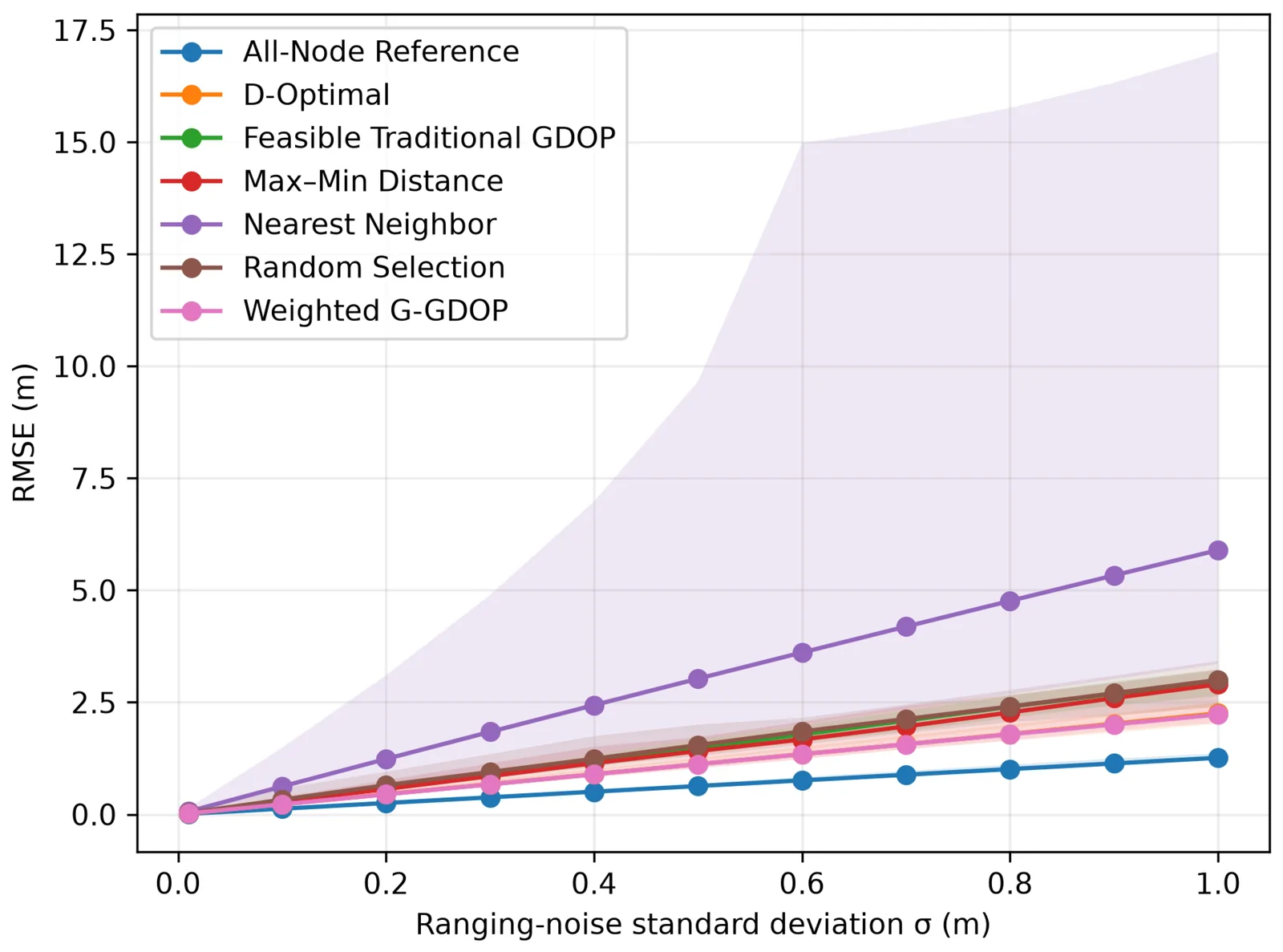
Rigid-aligned RMSE under increasing Gaussian ranging noise standard deviation σ for the tested methods.

Table 9, Table 10, Table 11 and Table 12 consolidate the Gaussian-noise, heterogeneous-noise, NLOS-bias, and online weight-estimation analyses. **Gaussian ranging noise (Panel A).** As σ increased from 0.01 to 1.0, the median RMSE of Weighted G-GDOP increased from 0.0223 to 2.228 m. Its median objective remained 0.01754, and its median runtime remained approximately 0.0756 s, because the candidate geometry and selection rule were unchanged across the injected Gaussian-noise levels. At σ=1.0, Weighted G-GDOP achieved a median RMSE of 2.228 m, compared with 2.256 m for D-Optimal, 2.976 m for Feasible Traditional GDOP, and 5.896 m for Nearest Neighbor. The resource-unconstrained All-Node Reference recorded a median RMSE of 1.262 m at this noise level; it is reported as a reference and is not treated as a universal accuracy bound. The complete RMSE trend is shown in Figure 9.

**Figure 10:**
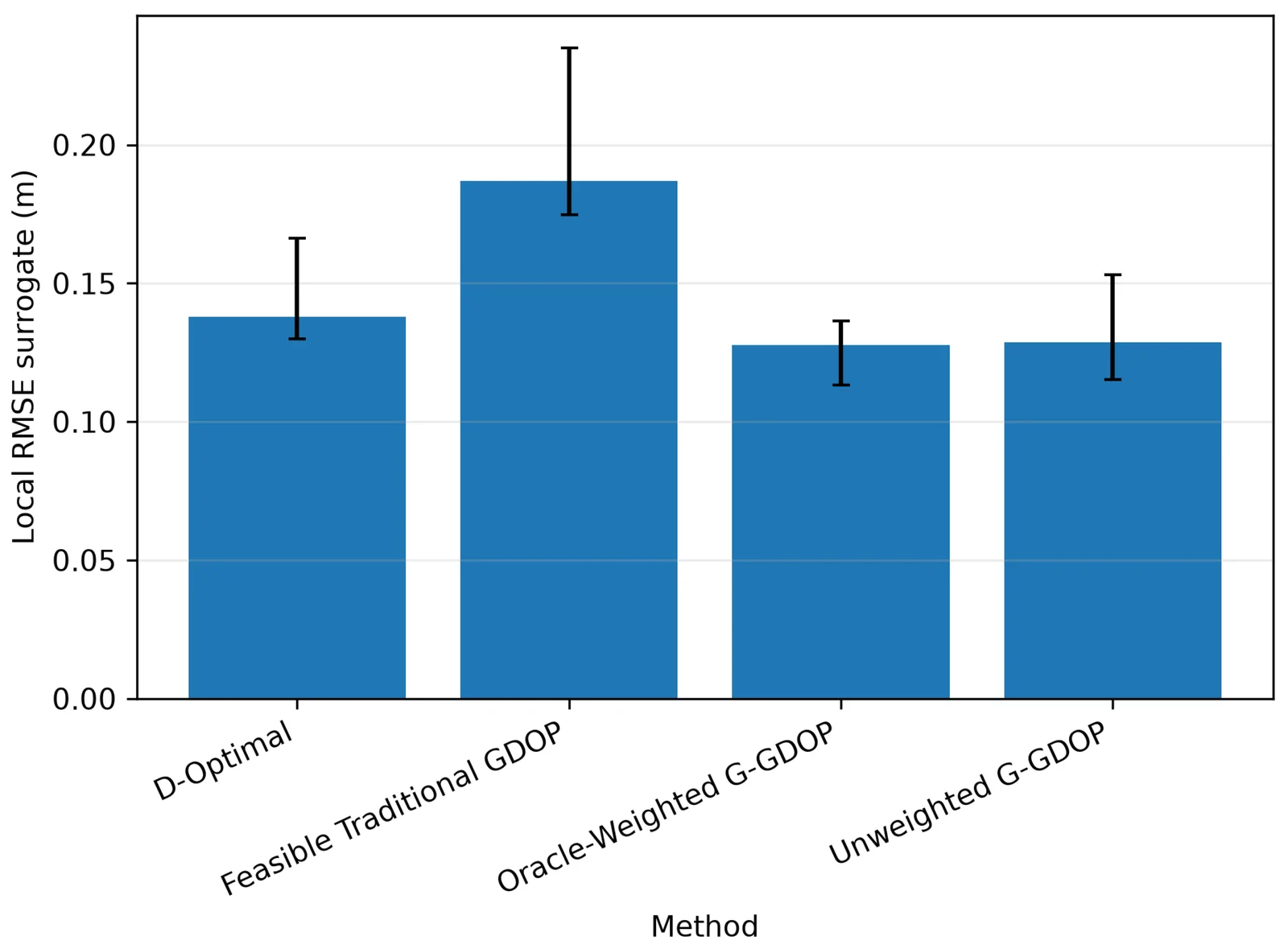
Local RMSE surrogate in the heterogeneous-noise ablation for D-Optimal, Feasible Traditional GDOP, Oracle-Weighted G-GDOP, and Unweighted G-GDOP.

**Heterogeneous-noise comparison (Panel B).** Oracle-Weighted G-GDOP attained the lowest median G-GDOP (11.710), local RMSE surrogate (0.1278), normalized objective (1.632), and empirical RMSE (0.1999 m). Unweighted G-GDOP was the closest comparator, with median objective and RMSE values of 1.696 and 0.2047 m, whereas Feasible Traditional GDOP yielded 3.264 and 0.2848 m. Median runtimes varied only from 0.1481 to 0.1536 s across the panel, indicating that the observed differences primarily reflect selection quality. Figure 10 compares the corresponding local RMSE surrogates.

**Figure 11:**
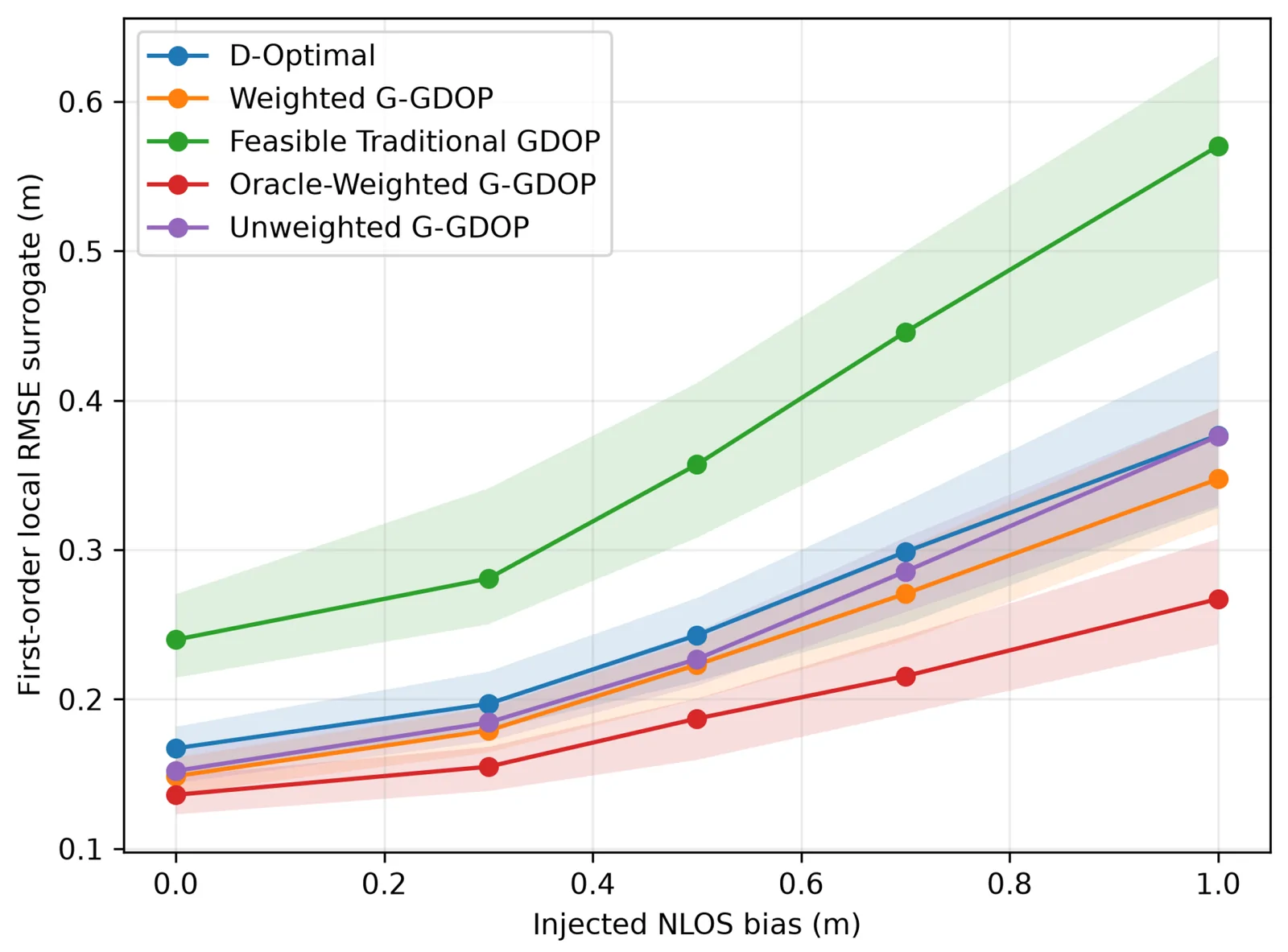
First-order local RMSE surrogate versus the injected NLOS bias for the tested methods.

**NLOS-bias sensitivity (Panel C).** Both the local surrogate and empirical RMSE deteriorated as the positive NLOS bias increased. The median RMSE of Oracle-Weighted G-GDOP rose from 0.2078 to 0.4033 m between bias levels of 0 and 1.0 m, while Weighted G-GDOP increased from 0.2197 to 0.5946 m. At the largest bias, Weighted G-GDOP retained a local surrogate of 0.3475, compared with 0.3767 for D-Optimal, 0.3761 for Unweighted G-GDOP, and 0.5703 for Feasible Traditional GDOP. Because the NLOS-link ratios and runtimes changed little with bias, the degradation is associated with bias exposure rather than a change in the nominal selection budget. Figure 11 shows the first-order local-surrogate degradation with increasing bias.

**Figure 12:**
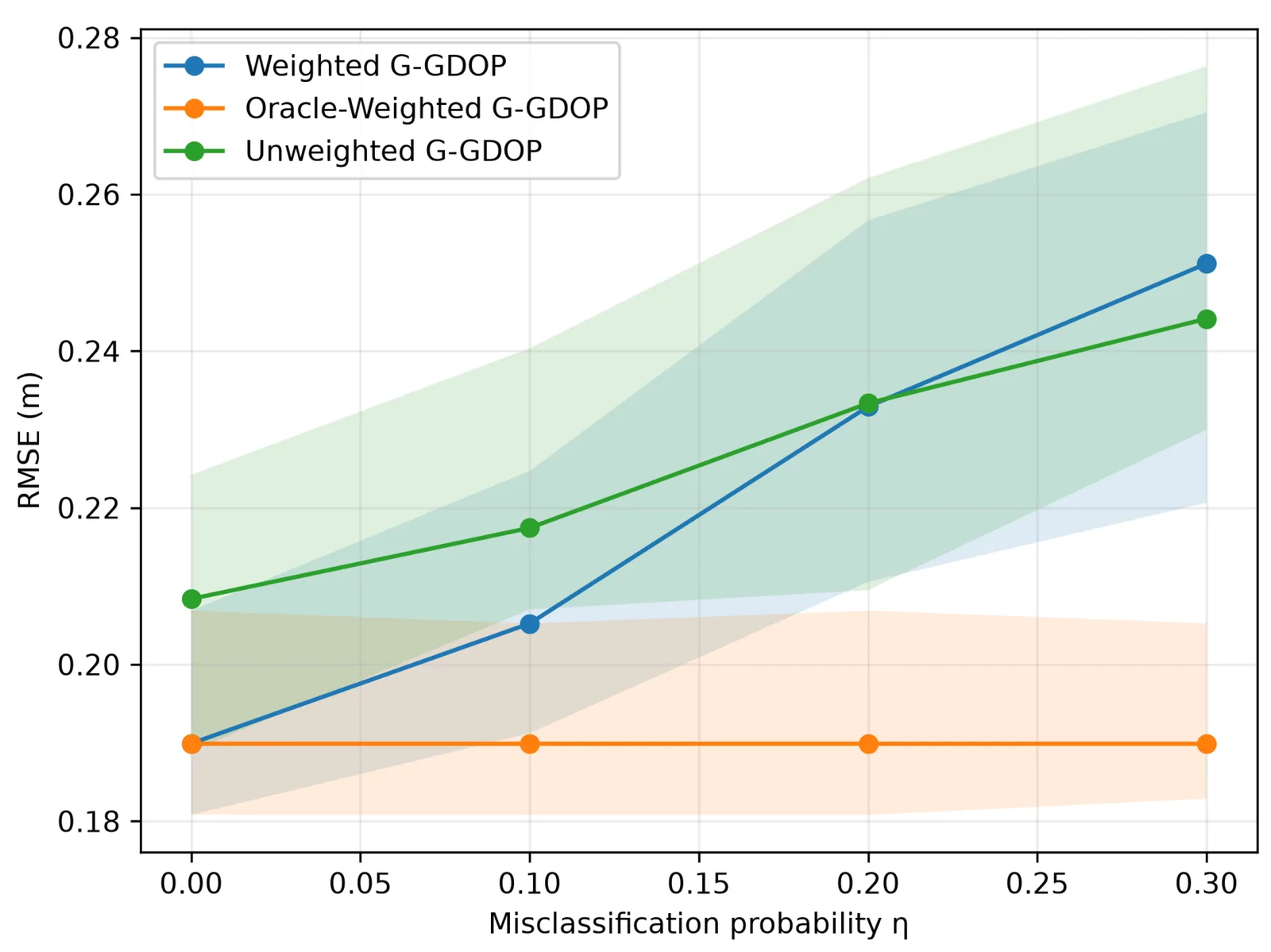
RMSE sensitivity to the link-quality misclassification probability η at ρ=0 for Weighted G-GDOP, Oracle-Weighted G-GDOP, and Unweighted G-GDOP.

**Online weight-estimation errors (Panel D).** Each η–ρ condition included 97 paired trials. At η=ρ=0, the median relative true-objective difference, true-objective difference, and subset Jaccard difference between Weighted G-GDOP and the oracle were all zero. At the largest tested perturbation, η=ρ=0.3, the corresponding median differences were 0.0621, 0.0735, and −0.3333. Across the complete η–ρ grid, Unweighted G-GDOP retained a median relative true-objective difference of 0.1400. Weighted G-GDOP therefore remained closer to the oracle objective throughout the tested perturbation grid, although subset agreement decreased as the weight errors increased. Figure 12 shows the RMSE response along the ρ=0 slice.

#### 4.2.5. Numerical Equivalence and Computational Scalability

Across all 24 tested network-size–graph-regime combinations, Direct Inversion and Woodbury Updating were numerically equivalent under the stated np.isclose tolerance (rtol = $1\;\times \;10^{-8}$ and atol = $1\;\times \;10^{-8}$). Across the 589 common-success paired groups, the maximum absolute objective difference was $1.376\times 10^{-8}$, confirming that the Woodbury update preserves the objective values to numerical precision. For runtime, Woodbury Updating was faster in 20 of the 24 tested size–regime combinations; the largest median speedup was 1.947 at N=100 in the fixed-radius regime. Complete condition-by-condition numerical-equivalence and runtime results are provided in Supplementary Tables S1 and S2, respectively.

**Figure 13:**
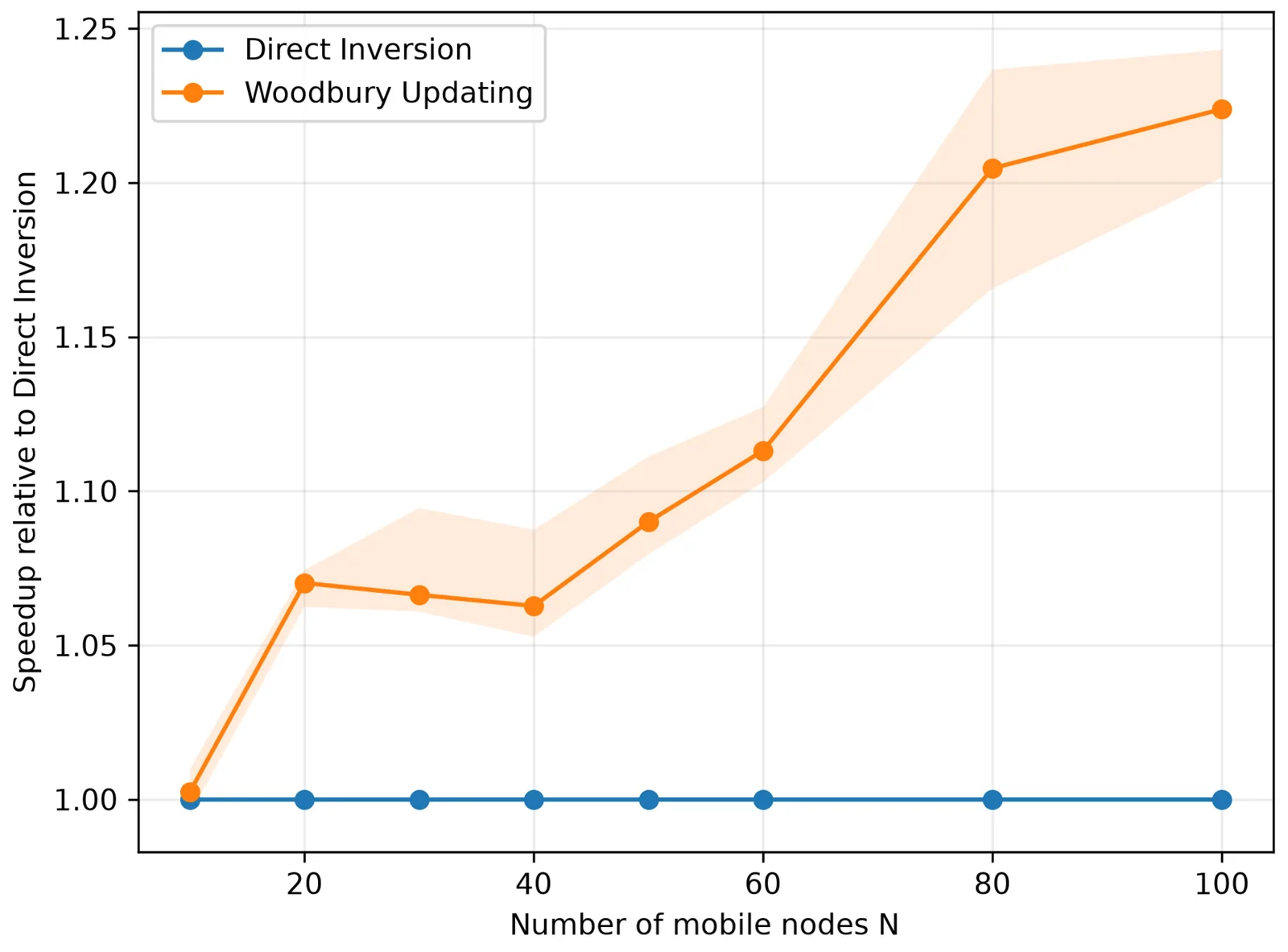
Runtime speedup relative to Direct Inversion versus network size *N* in the dense graph regime. A speedup above one favors Woodbury Updating.

Figure 13 visualizes the runtime speedup relative to Direct Inversion as network size increases in the dense-graph regime.

#### 4.2.6. Sensitivity to Predicted-Position Uncertainty

The controlled position uncertainty experiment shows that errors in the predicted geometry first affect the online information objective and subset choice, whereas the downstream nonlinear localization error changes more gradually. At the default setting of $\sigma_{p}=2\;m$ per coordinate, the median realized 3-D position RMSE was 3.446m. The median absolute predicted-versus-true geometry objective mismatch $\delta_{\Phi }$ was 0.840% with a 95% bootstrap confidence interval of [0.626%,1.123%], while the median subset Jaccard index was 1.000 [1.000,1.000]. The median true-geometry selection gap $\Delta_{\Phi }^{\mathrm{sel}}$ and the paired nonlinear localization-RMSE difference were both zero at this default uncertainty level.

**Figure 14:**
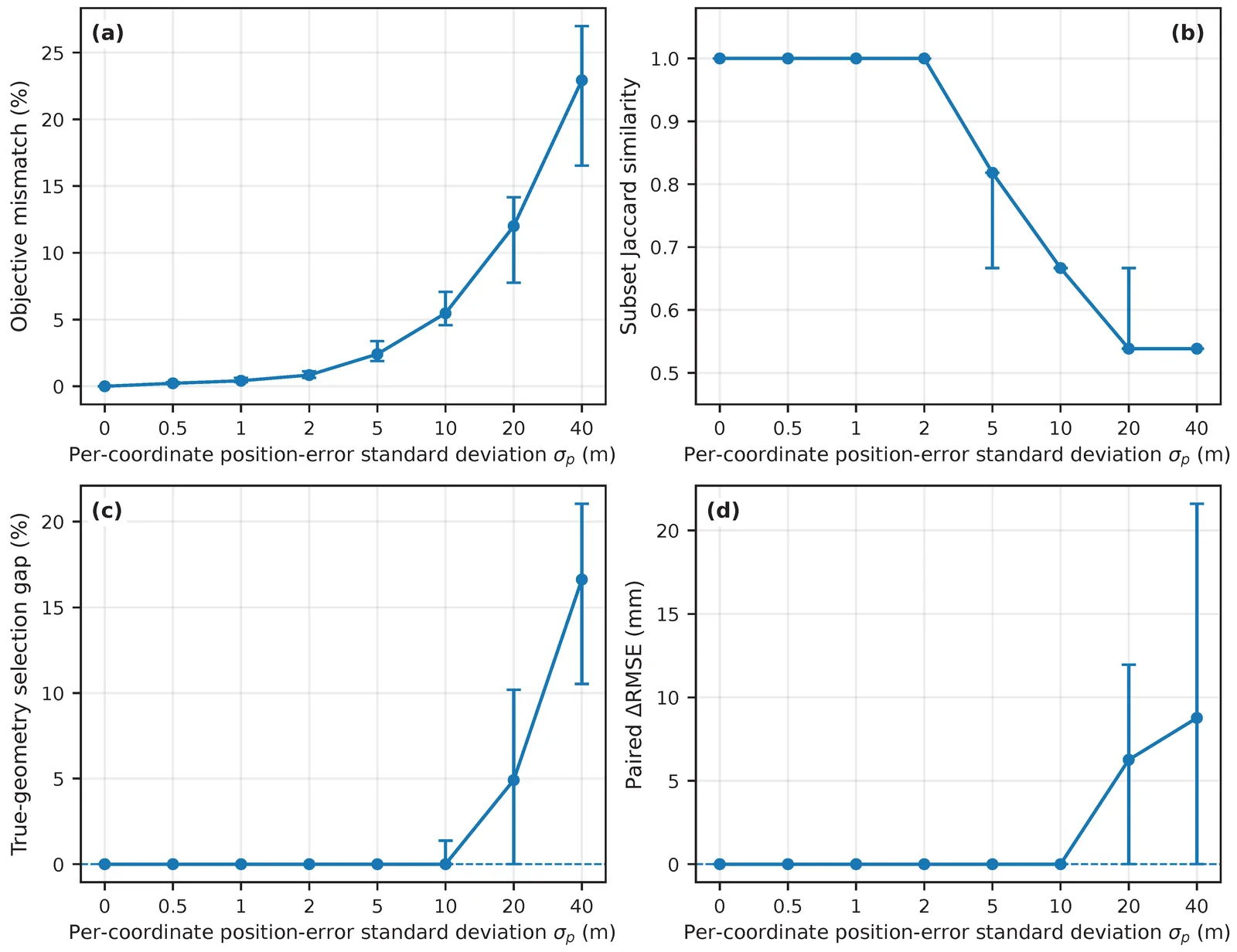
Sensitivity of cooperative sensor node pre-selection to predicted-position uncertainty. The per-coordinate prediction-error standard deviation $\sigma_{p}$ is varied using paired Gaussian error realizations while other trial-level quantities are held fixed. (**a**) Absolute relative mismatch of the A-optimal objective between predicted and true geometry for the selected node subset obtained using predicted geometry. (**b**) Jaccard similarity between selected node subsets obtained under predicted and true geometry. (**c**) Signed true-geometry objective gap relative to the true-geometry greedy reference. (**d**) Paired difference in rigid-aligned nonlinear localization RMSE. Markers show medians and error bars show pointwise 95% percentile bootstrap confidence intervals over the 97 true-geometry-reference-success trials.

As $\sigma_{p}$ increased, the median objective mismatch rose to 2.41%, 5.47%, 12.00%, and 22.93% at $\sigma_{p}=5$, 10, 20, and 40m, respectively, while the corresponding median subset Jaccard indices decreased to 0.818, 0.667, 0.538, and 0.538. The median true-geometry selection gap remained zero through $\sigma_{p}=10\;m$, then increased to 4.91% [0,9.49%] at 20m and 16.63% [10.53%,21.04%] at 40m. The paired nonlinear-RMSE difference remained comparatively small, with medians of 6.26mm [0,11.96mm] and 8.77mm [0,20.48mm] at the two largest uncertainty levels. Figure 14 summarizes these paired effects.

Three of the 100 trial groups were unobservable under the true-geometry reference and remained common-unobservable cases across all tested uncertainty levels. Among the 97 true-geometry-reference-success trials, the additional geometry-induced selection-failure rate, localization-failure rate, and predicted-versus-true observability-status-mismatch rate were each 0/97 = 0% at every tested $\sigma_{p}$ (95% Wilson CI: 0–3.81%).

### 4.3. Discussion and Practical Implications

The validation results define a clear applicability boundary for the proposed criterion. Across different topology realizations, G-GDOP is not treated as a direct predictor of the final error of a nonlinear localization pipeline because that error also depends on initialization, convergence, rigid alignment, and the realized noise and bias. Within a fixed topology and local linearization regime, however, the ordered normalized local RMSE values of 0.698, 1.014, and 1.980 for the Low, Medium, and High groups demonstrate that G-GDOP provides a meaningful information-quality ranking. It should therefore be interpreted as a local observability and structural-screening metric rather than as an absolute global-error predictor.

The resource experiments further show why the selected node subset and the induced active-link set must be distinguished. Raising *K* from 6 to 20 increased the median induced-link number from 109 to 214 and the median pre-selection time from 0.0578 to 0.0963 s, while reducing the median normalized objective from 7.994 to 0.777 and the median RMSE from 0.550 to 0.148 m. The ranging-slot sweep produced the same information–accuracy–resource pattern. At the full tested budget, Weighted G-GDOP approached the All-Node Reference while activating fewer links. The small-scale exhaustive comparison also showed that the absolute objective values became progressively closer as *K* increased, but this empirical result is not presented as a general approximation guarantee.

The K=3 result further identifies a boundary behavior of the two-stage heuristic. At this smallest tested budget, the observable seed consumed the entire node budget in every paired trial, so the large objective difference cannot be attributed to the post-seed A-optimal Stage II; that stage was never entered for an additional node selection iteration. Instead, the final selected node subset of size three was fixed entirely by the observability-first Stage I ordering. The diagnostic attribution showed that retained minimum eigenvalue was the dominant source of the first divergence from the exhaustive optimum, whereas deterministic node-ID tie-breaking explained a smaller number of cases. Induced-link, ranging-slot, and general resource increments did not explain the K=3 difference in this particular dense small-scale configuration because they were equal across the feasible candidate choices. Thus, K=3 should be viewed as an observable-seed boundary case rather than as a direct measure of the typical approximation behavior of the post-seed A-optimal greedy stage.

The robustness analyses identify two distinct sources of uncertainty: link-quality errors and predicted-geometry errors. Under heterogeneous ranging noise, link-dependent weighting improved the information objective and empirical RMSE relative to the tested baselines, whereas positive NLOS bias degraded both the local surrogate and nonlinear localization accuracy, confirming that covariance weighting alone does not remove deterministic bias. Online link-class and variance errors also increased the true-objective difference and reduced subset agreement relative to the oracle, although Weighted G-GDOP remained closer to the oracle objective than Unweighted G-GDOP over the tested perturbation grid.

The predicted-position experiment further shows that geometry uncertainty affects the information-based pre-selection stage before it produces a comparable change in downstream nonlinear localization accuracy. At the default $\sigma_{p}=2\;m$ per coordinate, the median predicted-versus-true objective mismatch was only 0.840%, the median subset Jaccard index remained 1.000, and both the median true-geometry selection gap and paired nonlinear-RMSE difference were zero. With increasing uncertainty, subset agreement decreased and the objective mismatch increased; at $\sigma_{p}=40\;m$, the median objective mismatch reached 22.93%, the median Jaccard index was 0.538, and the median true-geometry selection gap reached 16.63%. The corresponding median paired nonlinear-RMSE difference remained 8.77mm. No predicted-versus-true observability-status mismatch was observed, and no additional geometry-induced failure occurred among the 97 true-geometry-reference-success trials. These results support the use of predicted geometry under the tested default and moderate uncertainty levels, but they do not imply invariance to large position errors.

The implementation results likewise separate numerical correctness from runtime benefit. Direct Inversion and Woodbury Updating were numerically equivalent within the predefined tolerance in all 24 tested network-size and graph-regime combinations, while a Woodbury runtime advantage was supported in 20 conditions. The benefit was weaker in several small or sparse cases and reached its largest median speedup of 1.947 at N=100 in the fixed-radius regime. The recursive update should therefore be interpreted as a numerically consistent acceleration mechanism whose practical benefit depends on network size, graph structure, and update width rather than as an unconditional real-time guarantee.

From an engineering perspective, the proposed framework is a snapshot-level cooperative sensor node pre-selection layer intended to precede a downstream nonlinear or factor-graph estimator. The present experiments do not validate a specific GTSAM, iSAM2, or sliding-window implementation, and the normalized resource cost is not a hardware-calibrated energy measurement. Moreover, the controlled geometry experiment deliberately holds the candidate graph fixed and uses snapshot-wise Gaussian position perturbations; it therefore does not represent topology-discovery errors, temporally correlated state-prediction drift, latency, packet loss, or closed-loop dynamic state propagation. Link-quality uncertainty is represented by controlled class-confusion and variance-error models rather than by a measured classifier or a fully non-Gaussian UWB error model. The validation is also Monte Carlo based and does not include a public measured UWB dataset, hardware-in-the-loop testing, or UAV flight experiments. Accordingly, the practical claims are restricted to the information quality, subset selection, observability behavior, nonlinear RMSE, resource use, and computational behavior observed under the reported simulation conditions. Future work should investigate jointly uncertain geometry and topology, temporally correlated prediction errors, robust bias and outlier treatment, factor-graph integration, temporal scheduling, and validation using UWB-equipped UAV platforms or hardware-in-the-loop systems.

## 5. Conclusions

This paper developed a Fisher-information-based cooperative sensor node pre-selection framework for UWB-aided GNSS-denied UAV swarm localization. All mobile nodes remain in the localization state, while nodes in the resource-limited selected node subset are scheduled as active UWB ranging initiators, thereby inducing the active ranging-link set. The formulation separately represents the selected-node budget, induced-link number, ranging-slot occupation, residual-energy eligibility, and a normalized general resource cost.

A gauge-free projected Fisher information matrix was used to remove the rigid-body ambiguity of anchor-free range-only localization, and a trace-based G-GDOP/A-optimal criterion was constructed from link geometry, heterogeneous ranging reliability, and cooperative topology. A two-stage greedy heuristic first builds an observable seed and then uses marginal information gain with recursive Woodbury updates. The complexity analysis explicitly separates preprocessing, resource-feasibility screening, observable-seed construction, and post-seed candidate evaluation.

The nine formal experiments establish both the usefulness and the applicability boundary of the proposed method. G-GDOP did not serve as a direct predictor of global nonlinear recovery error, but it produced clear same-topology local-error ordering. Increasing node or slot resources improved the information objective and localization RMSE at the cost of more induced links and computation. Small-scale exhaustive enumeration provided an absolute optimality benchmark without implying a general approximation ratio. Gaussian-noise, heterogeneous-noise, NLOS-bias, online weight-error, and predicted-position-uncertainty experiments showed that link-dependent weighting is beneficial when its reliability estimates are informative, while deterministic bias, weight-estimation mismatch, and large predicted-position errors degrade information quality, localization performance, or subset agreement.

Direct Inversion and Woodbury Updating remained numerically consistent, while Woodbury provided a runtime advantage in most tested conditions. Its benefit nevertheless depended on network size and graph regime; the framework should therefore be viewed as an interpretable structural-screening module rather than an unconditional real-time or bias-robustness guarantee.

The present evidence remains limited to snapshot-level simulations with predicted-state and parameterized link-quality inputs. Future work will address dynamic and jointly uncertain topology/geometry, robust bias and outlier handling, factor-graph integration, hardware-calibrated resource models, and UWB-equipped UAV or hardware-in-the-loop validation.

## Figures and Tables

**Table 1:** Comparison of representative studies on cooperative localization and resource-aware sensor node and measurement selection.

Research Category	Main Focus	Considered Factors	Selection	Remaining Gap
GNSS-denied and collaborative positioning studies [1,2,3]	Collaborative positioning and range-based localization under degraded GNSS conditions	Relative ranging, IMU information, and cooperative constraints	Limited	Mainly focus on localization feasibility and sensor fusion rather than resource-constrained node/link selection
Multi-sensor fusion and factor-graph-based localization [4,5,6,7]	Fusion of UWB, IMU, VIO, and SLAM measurements	Measurement uncertainty and inter-platform ranging constraints	Limited	Focus mainly on estimation and fusion accuracy rather than selecting informative cooperative measurements
UAV swarm and multi-robot cooperative localization [10,11,12]	Cooperative localization for UAV or multi-agent systems in GNSS-denied environments	Formation constraints, clustering, and multi-condition positioning	Partial	Node optimization under limited ranging and communication resources is not fully addressed
GDOP- and geometry-based localization analysis [16,17]	Geometric quality evaluation and configuration optimization	Mainly observation geometry	Partial	Difficult to characterize heterogeneous ranging noise and multi-node topological coupling
FIM/EFIM- and CRLB-based system analysis [14,15,18]	System-level accuracy analysis and theoretical error bounds	Geometry, noise statistics, and network topology	Partial	Direct information-theoretic evaluation may involve high computational cost in dense networks
Existing sensor node and link selection methods [19,20,21,22,23]	Cooperative node activation, link selection, or resource allocation	Partial consideration of accuracy, noise, and resource constraints	Explicit	Accuracy–complexity balance and interpretability remain challenging
Proposed method	Fisher-information-based cooperative sensor node pre-selection	Gauge-free geometry, heterogeneous ranging noise, induced active links, and resource constraints	Explicit	Dynamic scenarios, factor-graph integration, and hardware validation

**Table 2:** Complexity comparison of exhaustive and greedy cooperative sensor node pre-selection procedures.

Procedure	Time Complexity	Main Dependence
Preprocessing	$O\left(N^{2}+Nd_{o}d_{g}+M_{c}d_{o}\right)$	Graph, gauge basis, and per-link vectors
Fixed-cardinality exhaustive search	ONKMc+Mcdo2+do3	Combinatorial subset enumeration
Resource-feasibility screening	$O\left[K_{\mathrm{out}}\left(N+M_{c}\right)\right]$	Candidate graph and resource budgets
Observable-seed construction	$O\left[\sum_{t<k_{\mathrm{seed}}}\sum_{i\in F_{t}}\left(d_{o}^{2}r_{i,t}+d_{o}^{3}\right)\right]$	Rank and eigenvalue tests
Post-seed direct-inversion greedy	$O\left[\sum_{t\geq k_{\mathrm{seed}}}\sum_{i\in F_{t}}\left(d_{o}^{2}r_{i,t}+d_{o}^{3}\right)\right]$	Full candidate inversion
Post-seed recursive greedy	$O\left[\sum_{t\geq k_{\mathrm{seed}}}\sum_{i\in F_{t}}\left(d_{o}^{2}r_{i,t}+d_{o}r_{i,t}^{2}+r_{i,t}^{3}\right)\right]$	Woodbury update width
Sparse bounded-degree special case	$O\left[\left(K_{\mathrm{out}}-k_{\mathrm{seed}}\right)Nd_{o}^{2}\right]$	Post-seed stage only

**Table 3:** General simulation parameter settings.

Parameter	Description	Default Value
Simulation region	Three-dimensional cooperative localization space	1000m×1000m×300m
*N*	Total number of UAV-borne mobile sensor nodes	30
*K*	Number of selected nodes	10
$R_{c}$	Communication/ranging radius	450m
σ	Standard deviation of ranging noise	0.1m
Node distribution	Spatial distribution of UAV nodes	Three-dimensional random distribution
Localization scenario	Cooperative localization mode	Anchor-free full-state inter-node ranging localization
RMSE calculation	Error calculation method	Calculated for all *N* mobile nodes after rigid alignment
Information metric calculation	Treatment of rigid-body gauge directions	Gauge-free projection with a numerical full-rank test
Gauge-rank threshold	Relative singular-value threshold for G	$\tau_{g}=10^{-10}$
Projected-FIM rank threshold	Relative eigenvalue threshold for $J_{o}$	$\tau_{J}=10^{-8}$
Active-link budget	Maximum number of induced active links	Experiment dependent
Ranging-slot model	Slot occupation of one active ranging link	$s_{e}=1$ by default
Ranging-slot budget	Maximum total ranging-slot occupation	Experiment dependent
Selected-node cost	Normalized cost of one selected node	$c_{i}^{\mathrm{node}}=1$ in the illustrative cost model
Active-link cost	Normalized cost of one induced ranging link	$c_{e}^{\mathrm{link}}=1$ in the illustrative cost model
General resource budget	Maximum normalized resource cost	Experiment dependent
Monte Carlo trials	Number of paired trials per operating condition	$R_{\mathrm{MC}}=100$
Bootstrap repetitions	Number of bootstrap resamples	$B_{\mathrm{boot}}=10,000$
Runtime repetitions	Repeated executions after one warm-up run	10
Statistical summary	Primary summary for continuous metrics	Median and pointwise 95% bootstrap CI
Binary-rate interval	Confidence interval for feasibility and success rates	95% Wilson CI

**Table 4:** True UWB link-quality classes and ranging-error parameters.

Link Class	Proportion	Random-Error Standard Deviation	Systematic Bias
LOS	50%	0.05m	0
wLOS	30%	0.10m	0
NLOS/degraded	20%	0.30m	0 or 0.30–1.00m

**Table 5:** Comparison methods and G-GDOP variants.

Method	Description
Random Selection	Randomly constructs one size-*K* selected node subset in each paired Monte Carlo trial using a pre-generated random seed and the same common resource-feasibility screening. An unobservable or localization-failed result is retained as a failure and is not redrawn.
Nearest Neighbor	A distance-priority local greedy node selection strategy.
Max–Min Distance	A geometric dispersion method that maximizes the minimum distance among the selected nodes.
D-Optimal	A node selection method that maximizes the determinant of the information matrix.
Feasible Traditional GDOP	A resource-feasible node selection method based on minimization of the traditional GDOP metric.
Unweighted G-GDOP	A G-GDOP-based method that considers topology-coupled multi-node information but does not use heteroscedastic link-noise weighting.
Weighted G-GDOP	The proposed G-GDOP-based method that jointly considers topology-coupled information and heteroscedastic link-noise weighting.
All-Node Reference	Activates all candidate nodes and all available induced ranging links. It is used as a resource-unconstrained reference rather than as an accuracy upper bound.

**Table 6:** Applicability-boundary and local-diagnostic summaries for G-GDOP.

**Global Applicability Diagnostics**			
**Metric**	**Valid Samples**	**Median [95% CI]**	**Unit**
G-GDOP	59	24.95 [19.97, 28.34]	–
Topology-level median RMSE	59	0.419 [0.326, 0.482]	m
**Local Diagnostic Consistency**			
**Metric**	**Valid Samples**	**Median [95% CI]**	**Unit**
Local CRLB proxy	59	0.1316 [0.0896, 0.1737]	$m^{2}$
Empirical local MSE	59	0.2723 [0.1976, 0.3419]	$m^{2}$
**Within-Topology Ranking**			
**G-GDOP Group**	**Valid Samples**	**Normalized Local RMSE [95% CI]**	
Low	20	0.698 [0.673, 0.752]	
Medium	20	1.014 [0.999, 1.068]	
High	20	1.980 [1.806, 2.180]	

*Notes:* Lower values indicate better information quality or localization accuracy. Shared non-success cases remain in the formal status accounting. The local CRLB proxy is $\sigma_{0}^{2}\Phi_{A}$ and therefore has unit $m^{2}$. Empirical local MSE is the median squared rigid-aligned RMSE over successful diagnostic repetitions and also has unit $m^{2}$. Normalized local RMSE is dimensionless. The table reports ordered groups only and does not report Pearson, Spearman, or coefficient-of-determination statistics.

**Table 7:** Information–accuracy–resource trade-offs under node and ranging-slot budgets.

**Node-Budget Trade-Off**					
*K*	Method	Links	Norm. objective	RMSE (m)	Time (s)
6	Weighted G-GDOP	109 [105.0, 115.5]	7.994 [5.636, 25.360]	0.550 [0.418, 0.814]	0.0578 [0.0571, 0.0584]
8	Weighted G-GDOP	128 [124.0, 131.0]	4.239 [3.412, 5.399]	0.321 [0.298, 0.368]	0.0687 [0.0678, 0.0693]
10	Weighted G-GDOP	145 [142.0, 149.0]	1.914 [1.729, 2.144]	0.233 [0.203, 0.249]	0.0750 [0.0742, 0.0764]
12	Weighted G-GDOP	164 [157.0, 166.0]	1.293 [1.154, 1.416]	0.187 [0.167, 0.201]	0.0807 [0.0798, 0.0819]
15	Weighted G-GDOP	186 [178.0, 188.0]	0.961 [0.877, 1.005]	0.160 [0.149, 0.170]	0.0877 [0.0866, 0.0887]
20	Weighted G-GDOP	214 [206.0, 218.0]	0.777 [0.723, 0.819]	0.148 [0.131, 0.159]	0.0963 [0.0953, 0.0975]
20	All-Node Reference	229 [224.0, 237.0]	0.745 [0.685, 0.783]	0.143 [0.131, 0.156]	0.0034 [0.0034, 0.0035]
**Ranging-Slot-Budget Trade-Off**					
0.35	Weighted G-GDOP	96 [96.0, 96.0]	99.347 [99.347, 99.347]	10.454 [10.454, 10.454]	0.0491 [0.0491, 0.0491]
0.45	Weighted G-GDOP	112 [107.0, 115.0]	6.694 [4.727, 11.572]	0.435 [0.363, 0.565]	0.0594 [0.0583, 0.0605]
0.55	Weighted G-GDOP	127 [125.0, 131.0]	3.529 [3.036, 5.098]	0.321 [0.283, 0.360]	0.0688 [0.0675, 0.0699]
0.70	Weighted G-GDOP	161 [156.0, 165.0]	1.312 [1.184, 1.451]	0.187 [0.171, 0.202]	0.0802 [0.0789, 0.0818]
1.00	Weighted G-GDOP	214 [206.0, 218.0]	0.777 [0.723, 0.820]	0.148 [0.131, 0.159]	0.0969 [0.0955, 0.0978]
1.00	All-Node Reference	229 [222.0, 237.0]	0.745 [0.685, 0.783]	0.143 [0.131, 0.156]	0.0035 [0.0034, 0.0035]

*Notes:* The All-Node Reference is included only at the matching full-budget endpoint. Shared structural non-success cases are not attributed to individual methods. The node-budget and ranging-slot-budget dimensions are kept in separate panels and are not collapsed into one sweep.

**Table 8:** Small-scale comparison of Weighted G-GDOP with exhaustive enumeration.

*K*	Method	Normalized Objective	Time (s)
3	Weighted G-GDOP	8.179 [6.520, 9.809]	0.0050 [0.0050, 0.0050]
3	Exhaustive optimum	3.901 [3.547, 4.195]	0.0308 [0.0307, 0.0309]
4	Weighted G-GDOP	2.210 [2.073, 2.442]	0.0059 [0.0059, 0.0059]
4	Exhaustive optimum	1.702 [1.590, 1.835]	0.0684 [0.0684, 0.0685]
5	Weighted G-GDOP	1.375 [1.291, 1.460]	0.0067 [0.0067, 0.0068]
5	Exhaustive optimum	1.250 [1.154, 1.344]	0.1094 [0.1093, 0.1095]
6	Weighted G-GDOP	1.060 [0.996, 1.149]	0.0074 [0.0074, 0.0075]
6	Exhaustive optimum	1.021 [0.933, 1.085]	0.1279 [0.1277, 0.1280]

*Notes:* Only absolute objective and runtime statistics are reported. The table does not state a general approximation ratio, optimality gap guarantee, or performance bound.

**Table 9:** Gaussian ranging noise robustness.

σ (m)	Method	Normalized Objective	RMSE (m)	Time (s)
0.01	All-Node Reference	0.00672 [0.00606, 0.00756]	0.0127 [0.0124, 0.0135]	0.0035 [0.0034, 0.0035]
0.01	D-Optimal	0.02109 [0.01786, 0.02298]	0.0225 [0.0207, 0.0251]	0.0752 [0.0745, 0.0763]
0.01	Feasible Traditional GDOP	3.42817 [2.74044, 3.98743]	0.0295 [0.0265, 0.0332]	0.0713 [0.0706, 0.0726]
0.01	Max–Min Distance	0.03256 [0.02311, 0.05777]	0.0274 [0.0248, 0.0390]	0.0040 [0.0039, 0.0041]
0.01	Nearest Neighbor	0.31088 [0.03210, 2.01513]	0.0627 [0.0332, 0.1441]	0.0043 [0.0040, 0.0044]
0.01	Random Selection	0.04212 [0.02986, 0.09134]	0.0326 [0.0279, 0.0586]	0.0041 [0.0038, 0.0041]
0.01	Weighted G-GDOP	0.01754 [0.01554, 0.02241]	0.0223 [0.0205, 0.0242]	0.0756 [0.0752, 0.0767]
0.50	All-Node Reference	0.00672 [0.00606, 0.00756]	0.6321 [0.6199, 0.6752]	0.0035 [0.0034, 0.0035]
0.50	D-Optimal	0.02109 [0.01789, 0.02298]	1.1204 [1.0351, 1.2555]	0.0752 [0.0745, 0.0763]
0.50	Feasible Traditional GDOP	3.42817 [2.73387, 3.98743]	1.4839 [1.3511, 1.6752]	0.0713 [0.0706, 0.0726]
0.50	Max–Min Distance	0.03256 [0.02311, 0.05777]	1.4142 [1.2119, 1.7212]	0.0040 [0.0039, 0.0041]
0.50	Nearest Neighbor	0.31088 [0.03210, 2.01513]	3.0273 [1.6688, 9.6621]	0.0043 [0.0040, 0.0044]
0.50	Random Selection	0.04212 [0.02986, 0.09134]	1.5451 [1.3018, 2.0021]	0.0041 [0.0038, 0.0041]
0.50	Weighted G-GDOP	0.01754 [0.01554, 0.02241]	1.1195 [1.0210, 1.2102]	0.0756 [0.0752, 0.0767]
1.00	All-Node Reference	0.00672 [0.00614, 0.00756]	1.2618 [1.2375, 1.3591]	0.0035 [0.0034, 0.0035]
1.00	D-Optimal	0.02109 [0.01786, 0.02410]	2.2556 [2.0280, 2.4969]	0.0752 [0.0745, 0.0764]
1.00	Feasible Traditional GDOP	3.42817 [2.73387, 4.05261]	2.9763 [2.6400, 3.2315]	0.0713 [0.0706, 0.0726]
1.00	Max–Min Distance	0.03252 [0.02311, 0.04825]	2.9006 [2.4124, 3.2275]	0.0040 [0.0039, 0.0041]
1.00	Nearest Neighbor	0.31088 [0.03210, 2.01513]	5.8956 [3.3559, 17.0095]	0.0043 [0.0040, 0.0044]
1.00	Random Selection	0.04212 [0.02986, 0.09134]	2.9996 [2.3935, 3.4209]	0.0041 [0.0038, 0.0041]
1.00	Weighted G-GDOP	0.01754 [0.01559, 0.02096]	2.2275 [2.0464, 2.4035]	0.0756 [0.0750, 0.0767]

*Notes:* The table retains representative lower, intermediate, and upper noise levels; the complete σ grid remains available in the formal results and figure.

**Table 10:** Heterogeneous-noise ablation.

Method	G-GDOP	Local RMSE Surrogate (m)	Normalized Objective	Empirical RMSE (m)	Time (s)
D-Optimal	12.634 [11.903, 15.229]	0.1379 [0.1299, 0.1662]	1.901 [1.682, 2.761]	0.2172 [0.2015, 0.2503]	0.1536 [0.1478, 0.1551]
Feasible Traditional GDOP	17.123 [16.017, 21.543]	0.1868 [0.1748, 0.2351]	3.264 [2.841, 3.927]	0.2848 [0.2550, 0.3269]	0.1481 [0.1435, 0.1513]
Oracle-Weighted G-GDOP	11.710 [10.406, 12.497]	0.1278 [0.1132, 0.1364]	1.632 [1.289, 1.859]	0.1999 [0.1844, 0.2230]	0.1528 [0.1473, 0.1551]
Unweighted G-GDOP	11.789 [10.515, 14.031]	0.1286 [0.1152, 0.1531]	1.696 [1.479, 2.017]	0.2047 [0.1885, 0.2300]	0.1526 [0.1493, 0.1544]

*Notes:* The first-order local RMSE surrogate is the square root of the corresponding local MSE surrogate and has unit m. It is a secondary local diagnostic and is not substituted for the empirical nonlinear localization RMSE.

**Table 11:** NLOS-bias sensitivity.

NLOS Bias (m)	Method	Local RMSE Surrogate (m)	Empirical RMSE (m)
0.0	D-Optimal	0.1672 [0.1500, 0.1816]	0.2430 [0.2197, 0.2670]
0.0	Weighted G-GDOP	0.1486 [0.1364, 0.1609]	0.2197 [0.2071, 0.2407]
0.0	Feasible Traditional GDOP	0.2399 [0.2144, 0.2703]	0.3373 [0.2999, 0.3756]
0.0	Oracle-Weighted G-GDOP	0.1361 [0.1230, 0.1486]	0.2078 [0.1878, 0.2278]
0.0	Unweighted G-GDOP	0.1521 [0.1440, 0.1651]	0.2542 [0.2302, 0.2697]
0.5	D-Optimal	0.2429 [0.2119, 0.2677]	0.3536 [0.3108, 0.4291]
0.5	Weighted G-GDOP	0.2231 [0.1999, 0.2400]	0.3622 [0.3160, 0.3993]
0.5	Feasible Traditional GDOP	0.3572 [0.3081, 0.4116]	0.5508 [0.4915, 0.6794]
0.5	Oracle-Weighted G-GDOP	0.1871 [0.1594, 0.2005]	0.2661 [0.2362, 0.3205]
0.5	Unweighted G-GDOP	0.2269 [0.2091, 0.2452]	0.3696 [0.3405, 0.4183]
1.0	D-Optimal	0.3767 [0.3280, 0.4338]	0.6081 [0.4981, 0.7154]
1.0	Weighted G-GDOP	0.3475 [0.3172, 0.3949]	0.5946 [0.5278, 0.6449]
1.0	Feasible Traditional GDOP	0.5703 [0.4820, 0.6306]	0.9528 [0.8430, 1.0891]
1.0	Oracle-Weighted G-GDOP	0.2672 [0.2367, 0.3073]	0.4033 [0.3579, 0.5093]
1.0	Unweighted G-GDOP	0.3761 [0.3296, 0.3943]	0.6148 [0.5366, 0.7010]

*Notes:* The local RMSE surrogate incorporates the true random-error covariance, estimator covariance, and residual bias. It has unit m and remains distinct from the rigid-aligned nonlinear empirical RMSE.

**Table 12:** Online link-weight error sensitivity.

η	ρ	Method/Reference	Pairs	Relative True-Objective Difference	True-Objective Difference	Subset-Jaccard Difference
0.0	0.0	Weighted G-GDOP	97	0.0000 [0.0000, 0.0000]	0.0000 [0.0000, 0.0000]	0.0000 [0.0000, 0.0000]
0.2	0.2	Weighted G-GDOP	97	0.0330 [0.0000, 0.0892]	0.0408 [0.0000, 0.0860]	−0.3333 [−0.3333, −0.1818]
0.3	0.3	Weighted G-GDOP	97	0.0621 [0.0019, 0.0967]	0.0735 [0.0067, 0.1041]	−0.3333 [−0.3333, −0.3333]
–	–	Unweighted G-GDOP (full-grid median)	97	0.1400	0.2351	−0.3333

*Notes:* All differences are calculated as the tested method minus Oracle-Weighted G-GDOP. Negative subset Jaccard differences indicate lower subset overlap. The unweighted row is an explicitly derived full-grid median, not an additional trial condition.

## Data Availability

The data presented in this study are available on request from the corresponding author.

## Supplementary Materials

The following supporting information can be downloaded at: https://www.mdpi.com/article/10.3390/s26165164/s1, Table S1: Numerical equivalence of Direct Inversion and Woodbury Updating across network sizes and graph regimes; Table S2: Runtime comparison of Direct Inversion and Woodbury Updating across all tested network sizes and graph regimes.

## Abbreviations

The following abbreviations are used in this manuscript:

UAV	Unmanned Aerial Vehicle
GNSS	Global Navigation Satellite System
UWB	Ultra-Wideband
IMU	Inertial Measurement Unit
LOS	Line-of-Sight
wLOS	Weak Line-of-Sight
NLOS	Non-Line-of-Sight
VIO	Visual-Inertial Odometry
SLAM	Simultaneous Localization and Mapping
GDOP	Geometric Dilution of Precision
G-GDOP	Generalized Geometric Dilution of Precision
FIM	Fisher Information Matrix
EFIM	Equivalent Fisher Information Matrix
CRLB	Cramér–Rao Lower Bound
RMSE	Root Mean Square Error
MDS	Multidimensional Scaling

## References

[1] Pascacio P., Casteleyn S., Torres-Sospedra J., Lohan E.-S., Nurmi J. (2021). Collaborative Indoor Positioning Systems: A Systematic Review. Sensors.

[2] Gabela J., Retscher G., Goel S., Perakis H., Masiero A., Toth C., Gikas V., Kealy A., Koppanyi Z., Blaszczak-Bak W. (2019). Experimental Evaluation of a UWB-Based Cooperative Positioning System for Pedestrians in GNSS-Denied Environment. Sensors.

[3] Han Y., Wei C., Li R., Wang J., Yu H. (2020). A Novel Cooperative Localization Method Based on IMU and UWB. Sensors.

[4] Wang R., Xu C., Li R., Duan S., Zhang X. (2024). Cooperative Localization and Mapping Based on UWB/IMU Fusion Using Factor Graphs. IEEE Sens. J..

[5] Cao Y., Beltrame G. (2021). VIR-SLAM: Visual, Inertial, and Ranging SLAM for Single and Multi-Robot Systems. Auton. Robot..

[6] Xu W., Chen Y., Liu S., Nie A., Chen R. (2025). Multi-Robot Cooperative Simultaneous Localization and Mapping Algorithm Based on Sub-Graph Partitioning. Sensors.

[7] Wang C., Li B., Duan Y., Sui X., Shi Z., Gao S., Zhang Z., Chen J. (2026). An Improved Robust ESKF Fusion Positioning Method with a Novel UWB-VIO Initialization. Sensors.

[8] Nowakowski M., Idzkowski A. Ultra-Wideband Signal Transmission According to European Regulations and Typical Pulses. Proceedings of the 2020 International Conference Mechatronic Systems and Materials (MSM).

[9] Efremova E.V., Kuzmin L.V. (2024). Wireless Ranging by Evaluating Received Signal Strength of UWB Chaotic Radio Pulses: Effects of Signal Propagation Conditions. Technologies.

[10] Li Q., Wang P., Li X., Zhang J., Luo Y., Yu W., Cheng H. (2026). Formation-Constrained Cooperative Localization for UAV Swarms in GNSS-Denied Environments. Sensors.

[11] Zhang S., Zhang H., Zhan Y., Wei X., Liu Y. (2025). Cluster Networking and Cooperative Localization Based on Biogeography Optimization and Improved Super-Multidimensional Scaling for Multi-Unmanned Aerial Vehicles. Sensors.

[12] Liu H., Jiang W., Long Q., Xia Q., Chen X. (2026). A High-Precision Cooperative Localization Method for UAVs Based on Multi-Condition Constraints. Sensors.

[13] Patwari N., Ash J.N., Kyperountas S., Hero A.O., Moses R.L., Correal N.S. (2005). Locating the Nodes: Cooperative Localization in Wireless Sensor Networks. IEEE Signal Process. Mag..

[14] Shen Y., Wymeersch H., Win M.Z. (2010). Fundamental Limits of Wideband Localization—Part II: Cooperative Networks. IEEE Trans. Inf. Theory.

[15] Win M.Z., Dai W., Shen Y. (2018). A Theoretical Foundation of Network Localization and Navigation. Proc. IEEE.

[16] Quan Q. The Tightest Geometry for Lowest GDOP in Range-Based 2-D Wireless Location Systems. Proceedings of the World Congress on Computer Science and Information Engineering.

[17] Zhou R., Sun H., Li H., Li W. (2020). TDOA and Track Optimization of UAV Swarm Based on D-Optimality. J. Syst. Eng. Electron..

[18] Dellaert F., Kaess M. (2017). Factor Graphs for Robot Perception. Found. Trends Robot..

[19] Bel A., Vicario J.L., Seco-Granados G. (2011). Localization Algorithm with On-Line Path Loss Estimation and Node Selection. Sensors.

[20] Tang C., Dou L. (2020). An Improved Game Theory-Based Cooperative Localization Algorithm for Eliminating the Conflicting Information of Multi-Sensors. Sensors.

[21] Yin T., Zou D., Zhang R., Jing D., Li Y., Lu X. (2022). Evolutionary Overlapping Coalitional Game-Based Link Selection for Distributed Cooperative Localization in Mobile Networks. Wirel. Commun. Mob. Comput..

[22] Qiu F., Zhang W. (2023). Efficient Cooperative Localization Method with Node Selection Based on Position Error Bound. Signal Process..

[23] Chen G., Cheng L., Zeng Q., Shen F., Zhang Y.-D. (2025). Willingness Allocation-Assisted Cooperative Localization Algorithm Based on Competitive Game for Resource-Constrained Environment. IEEE Trans. Green Commun. Netw..

