# Supplementary material for: Fisher-Information-Based Cooperative Sensor Node Pre-Selection for UWB-Aided GNSS-Denied UAV Swarm Localization Under Heterogeneous Ranging Noise
Source: Sensors (Basel). 2026 Aug 14;26(16):5164. doi: 10.3390/s26165164 (PMC13517213; doi:10.3390/s26165164)
Supplement: Supplementary file 1 [file sensors-26-05164-s001.zip › sensors-4424046-supplementary.pdf]

Supplementary Material

Manuscript ID: sensors-4424046

Supplementary Tables

The complete condition-by-condition numerical-equivalence and runtime results supporting the computational-scalability analysis in the main manuscript are provided below. Numerical values are reproduced from the corresponding manuscript tables without modification.

Table S1: Numerical equivalence of direct inversion and Woodbury updating across network sizes and graph regimes.

N	Regime	Common successes paired groups	Maximum absolute objective difference	Maximum normalized tolerance ratio
10	Dense	30/30	0	0
10	Fixed radius	8/8	4.583×10^{-13}	2.366×10^{-6}
10	Sparse	30/30	0	0
20	Dense	30/30	0	0
20	Fixed radius	22/22	8.399×10^{-12}	1.394×10^{-5}
20	Sparse	28/28	9.415×10^{-14}	1.038×10^{-6}
30	Dense	30/30	0	0
30	Fixed radius	30/30	0	0
30	Sparse	23/23	2.451×10^{-12}	1.041×10^{-5}
40	Dense	27/27	0	0
40	Fixed radius	30/30	0	0
40	Sparse	10/10	1.376×10^{-8}	1.388×10^{-3}
50	Dense	28/28	0	0
50	Fixed radius	30/30	0	0
50	Sparse	21/21	1.393×10^{-12}	2.581×10^{-6}
60	Dense	29/29	0	0
60	Fixed radius	30/30	0	0
60	Sparse	18/18	4.000×10^{-12}	1.662×10^{-5}
80	Dense	28/28	0	0
80	Fixed radius	30/30	0	0
80	Sparse	12/12	0	0
100	Dense	24/24	0	0
100	Fixed radius	30/30	0	0
100	Sparse	11/11	4.031×10^{-9}	8.812×10^{-4}

Notes: All 24 size–regime combinations are equivalent under `np.isclose` with `rtol=1e-8` and `atol=1e-8`. This establishes numerical equivalence within the stated tolerance, not bitwise identity. Common-unobservable groups remain in status accounting and are excluded from paired runtime statistics. Speedup is direct-inversion runtime divided by Woodbury runtime.

Table S2: Runtime comparison of direct inversion and Woodbury updating across all tested network sizes and graph regimes.

N	Regime	Direct time (s)	Woodbury time (s)	Speedup	Runtime conclusion
10	Dense	0.0056 [0.0056, 0.0056]	0.0056 [0.0056, 0.0057]	1.002 [0.995, 1.010]	No clear difference
10	Fixed radius	0.0057 [0.0056, 0.0058]	0.0057 [0.0056, 0.0058]	1.002 [0.995, 1.012]	No clear difference
10	Sparse	0.0056 [0.0056, 0.0057]	0.0056 [0.0056, 0.0057]	1.005 [0.999, 1.013]	No clear difference
20	Dense	0.0222 [0.0222, 0.0223]	0.0208 [0.0207, 0.0210]	1.070 [1.062, 1.074]	Woodbury advantage
20	Fixed radius	0.0240 [0.0238, 0.0245]	0.0236 [0.0233, 0.0244]	1.014 [1.007, 1.025]	Woodbury advantage
20	Sparse	0.0239 [0.0235, 0.0245]	0.0228 [0.0226, 0.0240]	1.036 [1.026, 1.049]	Woodbury advantage
30	Dense	0.0784 [0.0762, 0.0791]	0.0735 [0.0691, 0.0742]	1.066 [1.061, 1.094]	Woodbury advantage
30	Fixed radius	0.0781 [0.0769, 0.0794]	0.0731 [0.0725, 0.0741]	1.067 [1.063, 1.086]	Woodbury advantage
30	Sparse	0.0840 [0.0816, 0.0845]	0.0807 [0.0803, 0.0839]	1.014 [1.007, 1.026]	Woodbury advantage
40	Dense	0.2188 [0.2154, 0.2219]	0.2071 [0.2007, 0.2100]	1.063 [1.053, 1.087]	Woodbury advantage
40	Fixed radius	0.2129 [0.2080, 0.2197]	0.1886 [0.1794, 0.1932]	1.148 [1.135, 1.171]	Woodbury advantage
40	Sparse	0.2238 [0.2209, 0.2258]	0.2241 [0.2190, 0.2251]	1.000 [0.988, 1.024]	No clear difference

Table S2: Runtime comparison of direct inversion and Woodbury updating across all tested network sizes and graph regimes. (continued)

N	Regime	Direct time (s)	Woodbury time (s)	Speedup	Runtime conclusion
50	Dense	0.5051 [0.4977, 0.5085]	0.4635 [0.4443, 0.4736]	1.090 [1.079, 1.111]	Woodbury advantage
50	Fixed radius	0.4743 [0.4690, 0.4818]	0.3752 [0.3669, 0.3832]	1.270 [1.249, 1.279]	Woodbury advantage
50	Sparse	0.5252 [0.5190, 0.5325]	0.5125 [0.4998, 0.5282]	1.028 [1.006, 1.066]	Woodbury advantage
60	Dense	1.0384 [1.0255, 1.0496]	0.9277 [0.9142, 0.9450]	1.113 [1.103, 1.127]	Woodbury advantage
60	Fixed radius	0.9912 [0.9871, 1.0037]	0.7542 [0.7301, 0.7662]	1.330 [1.304, 1.349]	Woodbury advantage
60	Sparse	1.0581 [1.0420, 1.0844]	1.0388 [1.0124, 1.0735]	1.042 [1.007, 1.058]	Woodbury advantage
80	Dense	3.3919 [3.3635, 3.4337]	2.8501 [2.7594, 2.9195]	1.205 [1.166, 1.237]	Woodbury advantage
80	Fixed radius	3.6380 [3.4479, 4.0743]	2.5553 [2.3857, 2.8459]	1.434 [1.402, 1.465]	Woodbury advantage
80	Sparse	3.5199 [3.4844, 3.5566]	3.3839 [3.2692, 3.4274]	1.040 [1.028, 1.068]	Woodbury advantage
100	Dense	8.7888 [8.7434, 19.6070]	7.2305 [7.0962, 15.9296]	1.224 [1.202, 1.243]	Woodbury advantage
100	Fixed radius	26.3068 [26.1856, 27.6644]	14.3888 [13.9896, 15.0866]	1.947 [1.855, 2.008]	Woodbury advantage
100	Sparse	32.3985 [32.1735, 32.5875]	31.5012 [31.2998, 32.2365]	1.029 [1.014, 1.038]	Woodbury advantage

Notes: All 24 size–regime combinations are equivalent under `np.isclose` with `rtol=1e-8` and `atol=1e-8`. This establishes numerical equivalence within the stated tolerance, not bitwise identity. Common-unobservable groups remain in status accounting and are excluded from paired runtime statistics. Speedup is direct-inversion runtime divided by Woodbury runtime.
